# Supplementary material for: PrEP (Pre-Exposure Prophylaxis) Education for Clinicians: Caring for an MSM Patient
Source: MedEdPORTAL. 2020 May 29;16:10908. doi: 10.15766/mep_2374-8265.10908 (PMC7336890; doi:10.15766/mep_2374-8265.10908)
Supplement: Supplementary file 1 — Presentation.pptxPresentation with Audio.pptxDiscussion Guide.docxPatient-Physician Video.mp4Preworkshop Evaluation.docxPostworkshop Evaluation.docx [file mep_2374-8265.10908-s001.zip › A. Presentation.pptx]

## Slide 1
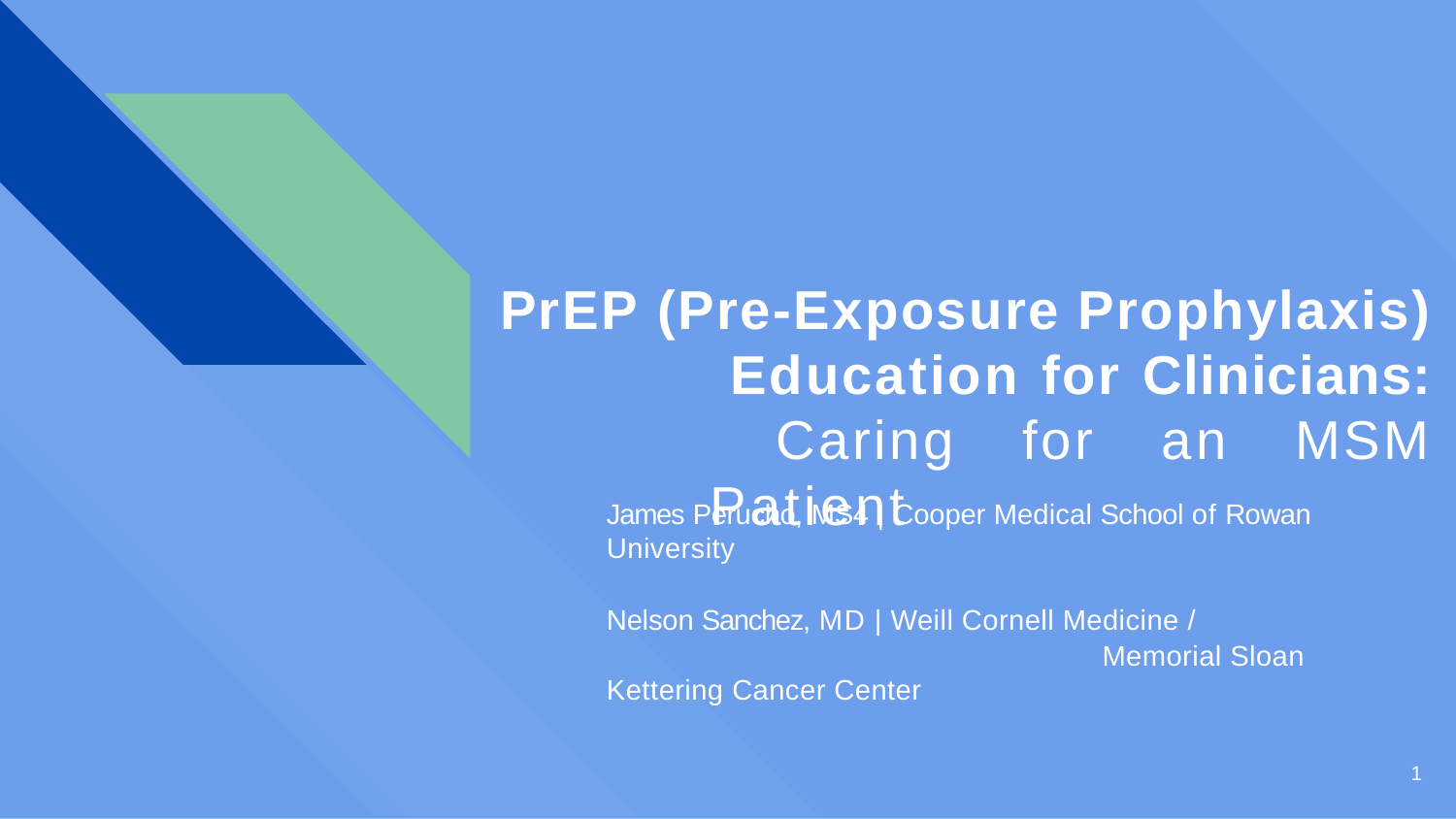

# PrEP (Pre-Exposure Prophylaxis) Education for Clinicians: Caring for an MSM Patient
James Perucho, MS4 | Cooper Medical School of Rowan University
Nelson Sanchez, MD | Weill Cornell Medicine /
			 Memorial Sloan Kettering Cancer Center
1

## Slide 2
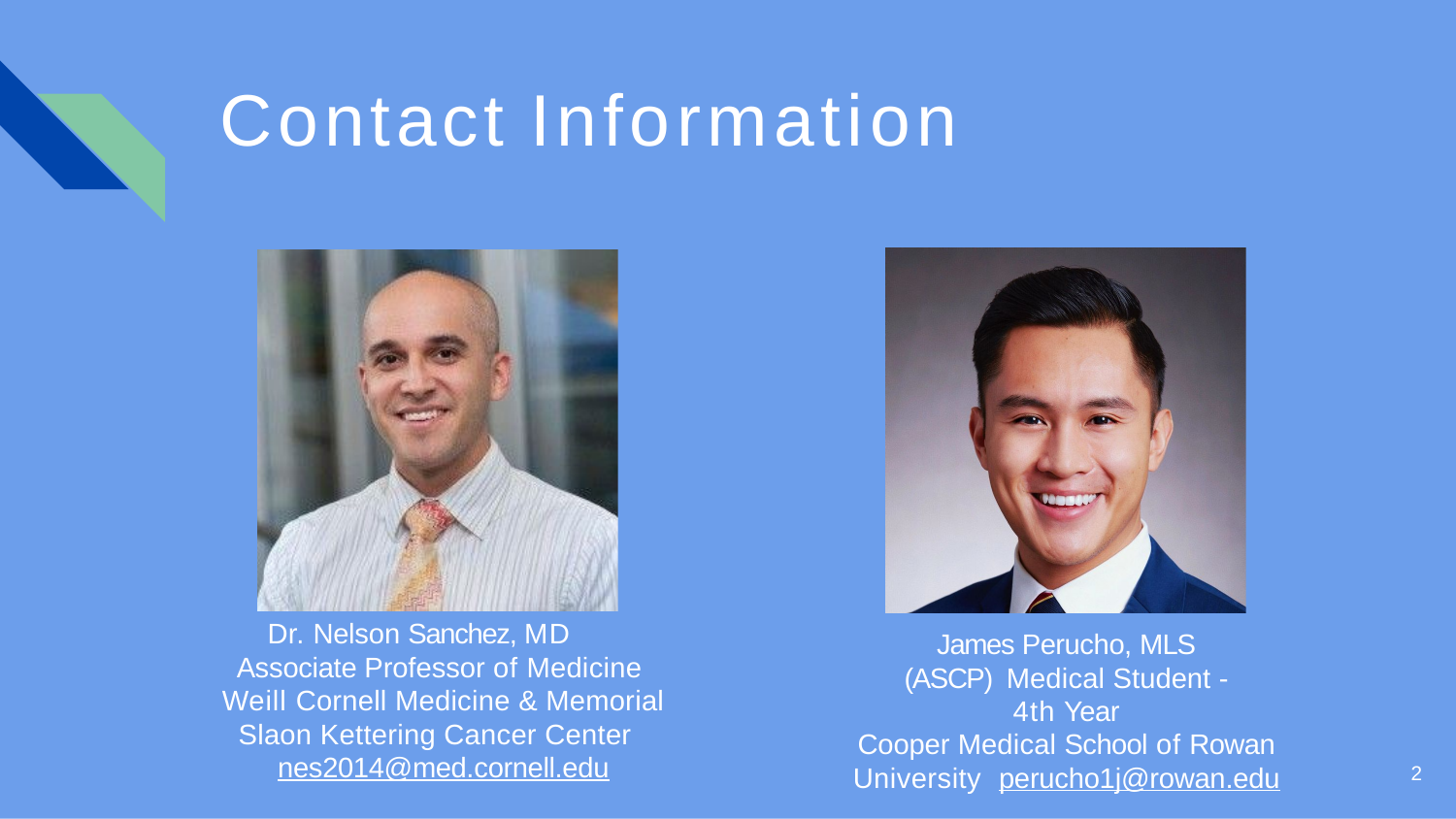

# Contact Information
Dr. Nelson Sanchez, MD Associate Professor of Medicine Weill Cornell Medicine & Memorial Slaon Kettering Cancer Center nes2014@med.cornell.edu
James Perucho, MLS (ASCP) Medical Student - 4th Year
Cooper Medical School of Rowan University perucho1j@rowan.edu
2

## Slide 3
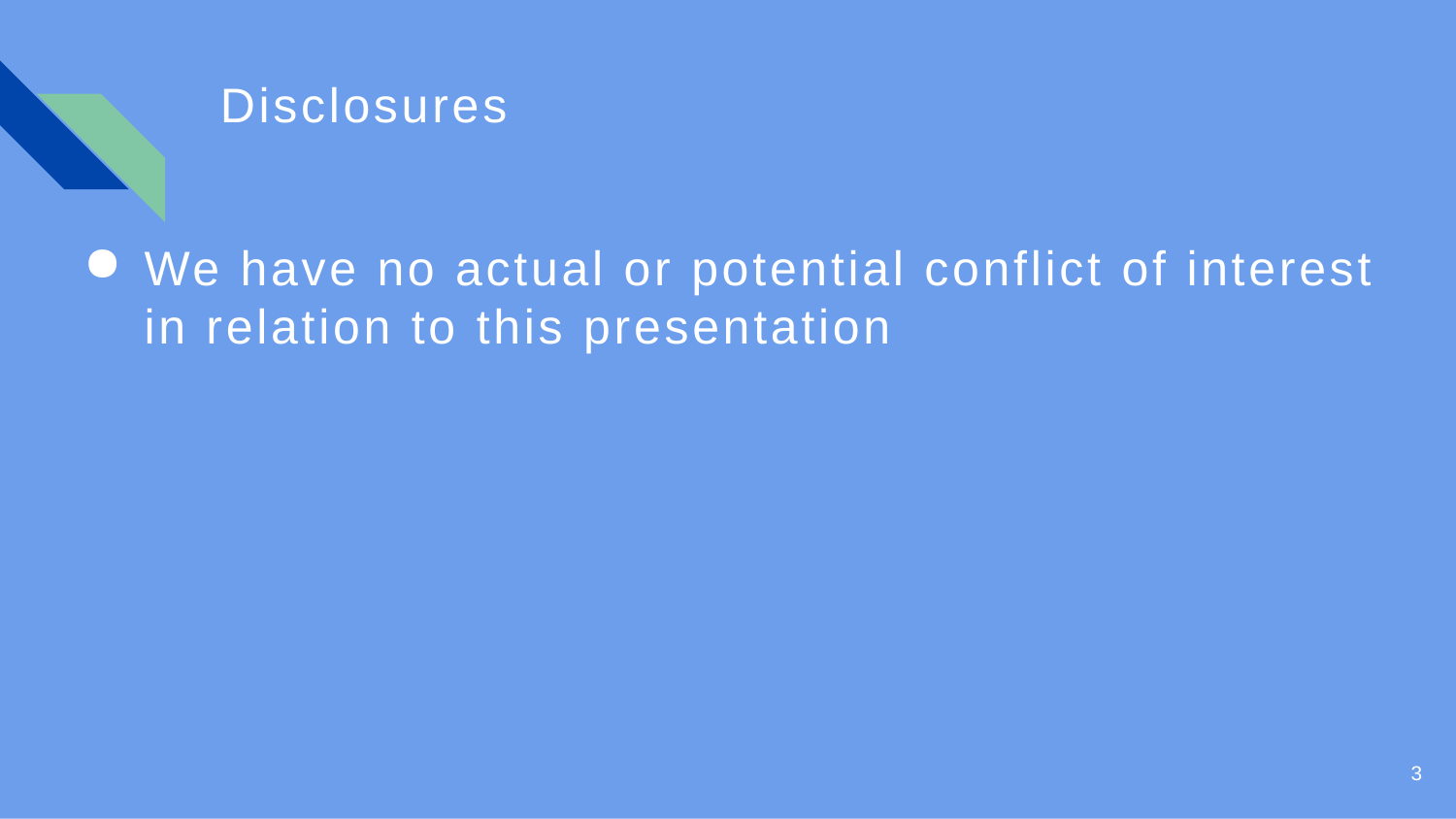

# Disclosures
We have no actual or potential conflict of interest in relation to this presentation
3

## Slide 4
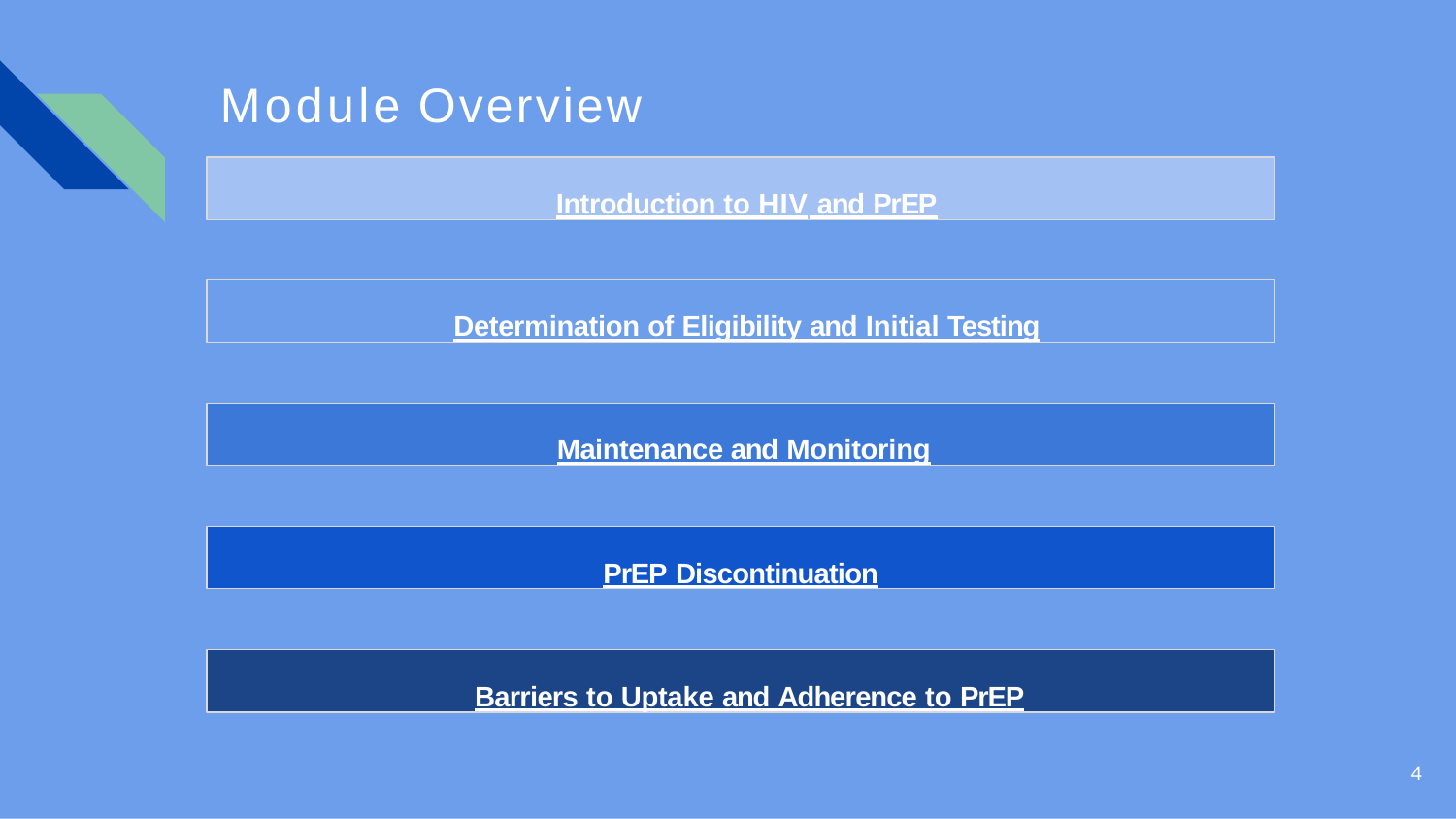

# Module Overview
Introduction to HIV and PrEP
Determination of Eligibility and Initial Testing
Maintenance and Monitoring
PrEP Discontinuation
Barriers to Uptake and Adherence to PrEP
4

## Slide 5
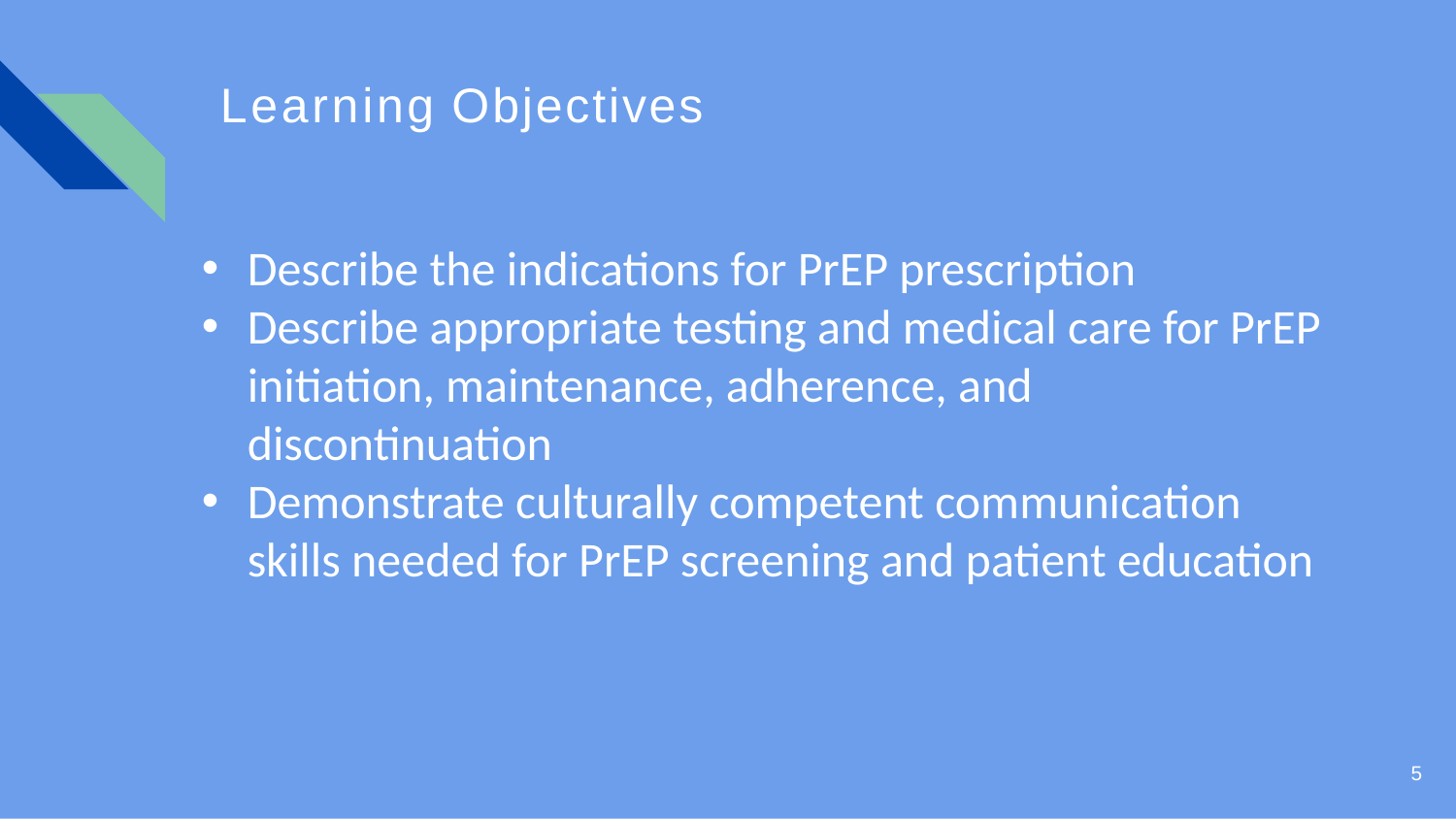

# Learning Objectives
Describe the indications for PrEP prescription
Describe appropriate testing and medical care for PrEP initiation, maintenance, adherence, and discontinuation
Demonstrate culturally competent communication skills needed for PrEP screening and patient education
5

## Slide 6
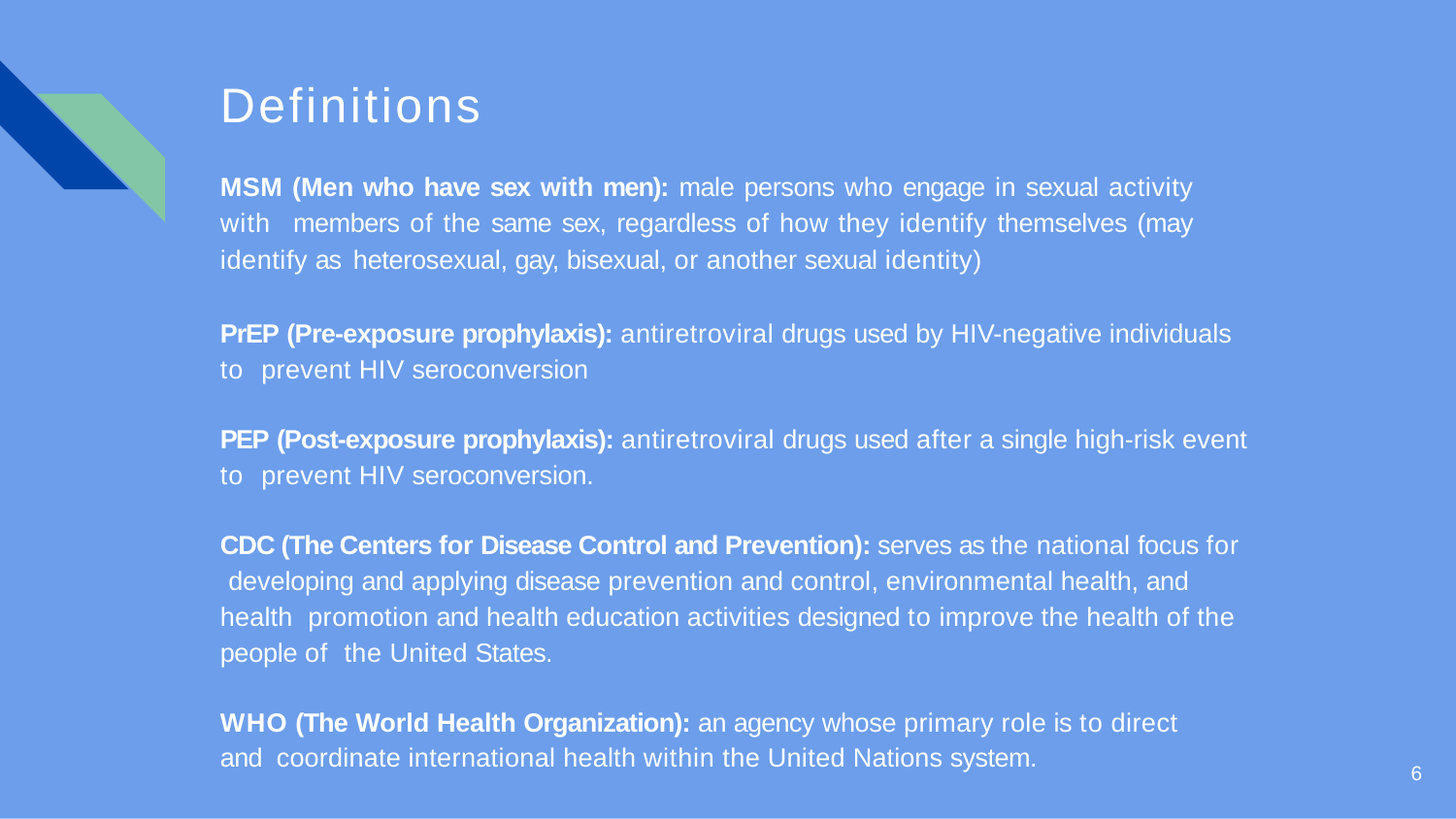

# Definitions
MSM (Men who have sex with men): male persons who engage in sexual activity with members of the same sex, regardless of how they identify themselves (may identify as heterosexual, gay, bisexual, or another sexual identity)
PrEP (Pre-exposure prophylaxis): antiretroviral drugs used by HIV-negative individuals to prevent HIV seroconversion
PEP (Post-exposure prophylaxis): antiretroviral drugs used after a single high-risk event to prevent HIV seroconversion.
CDC (The Centers for Disease Control and Prevention): serves as the national focus for developing and applying disease prevention and control, environmental health, and health promotion and health education activities designed to improve the health of the people of the United States.
WHO (The World Health Organization): an agency whose primary role is to direct and coordinate international health within the United Nations system.
6

## Slide 7
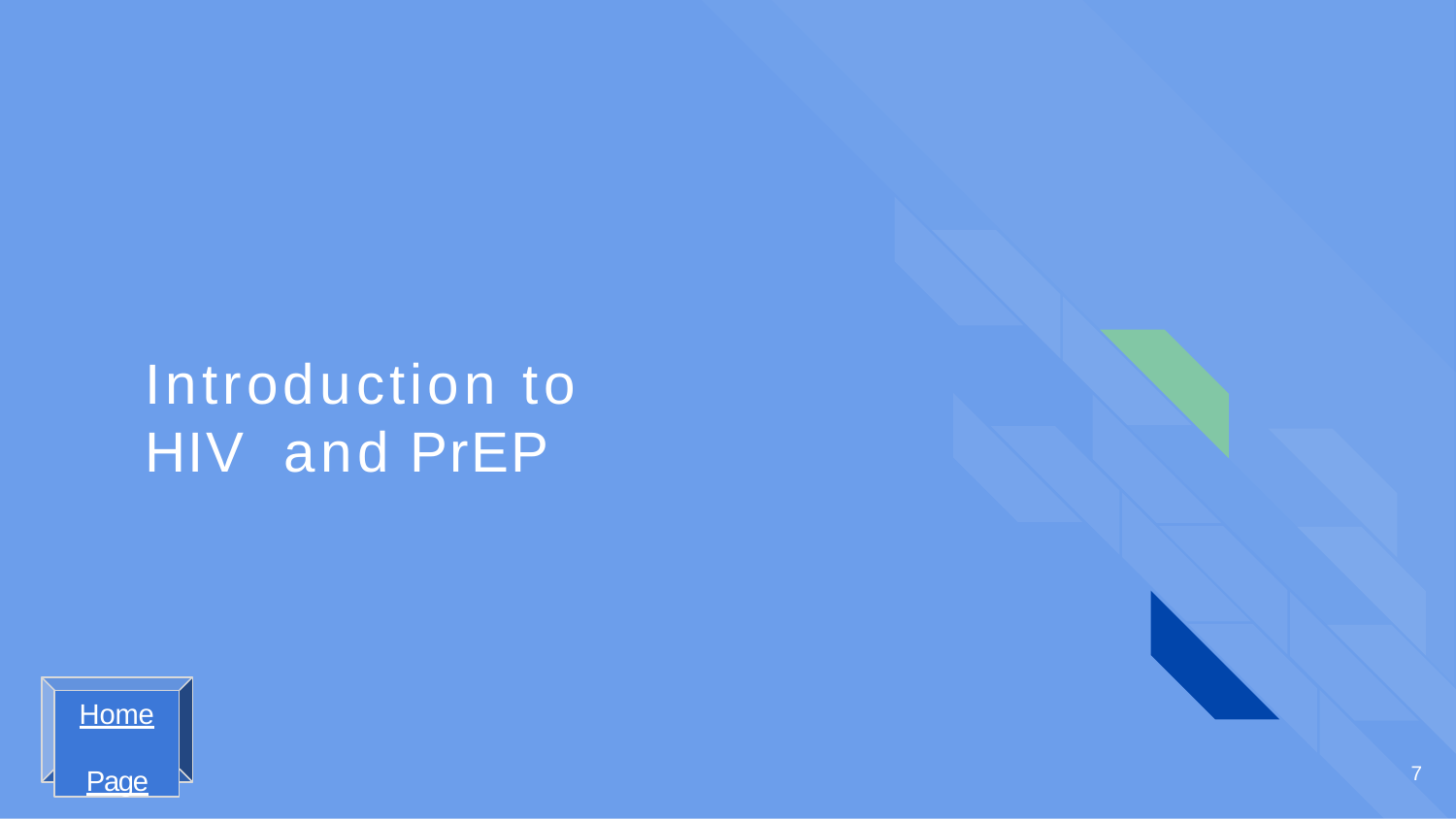

# Introduction to HIV and PrEP
Home Page
7

## Slide 8
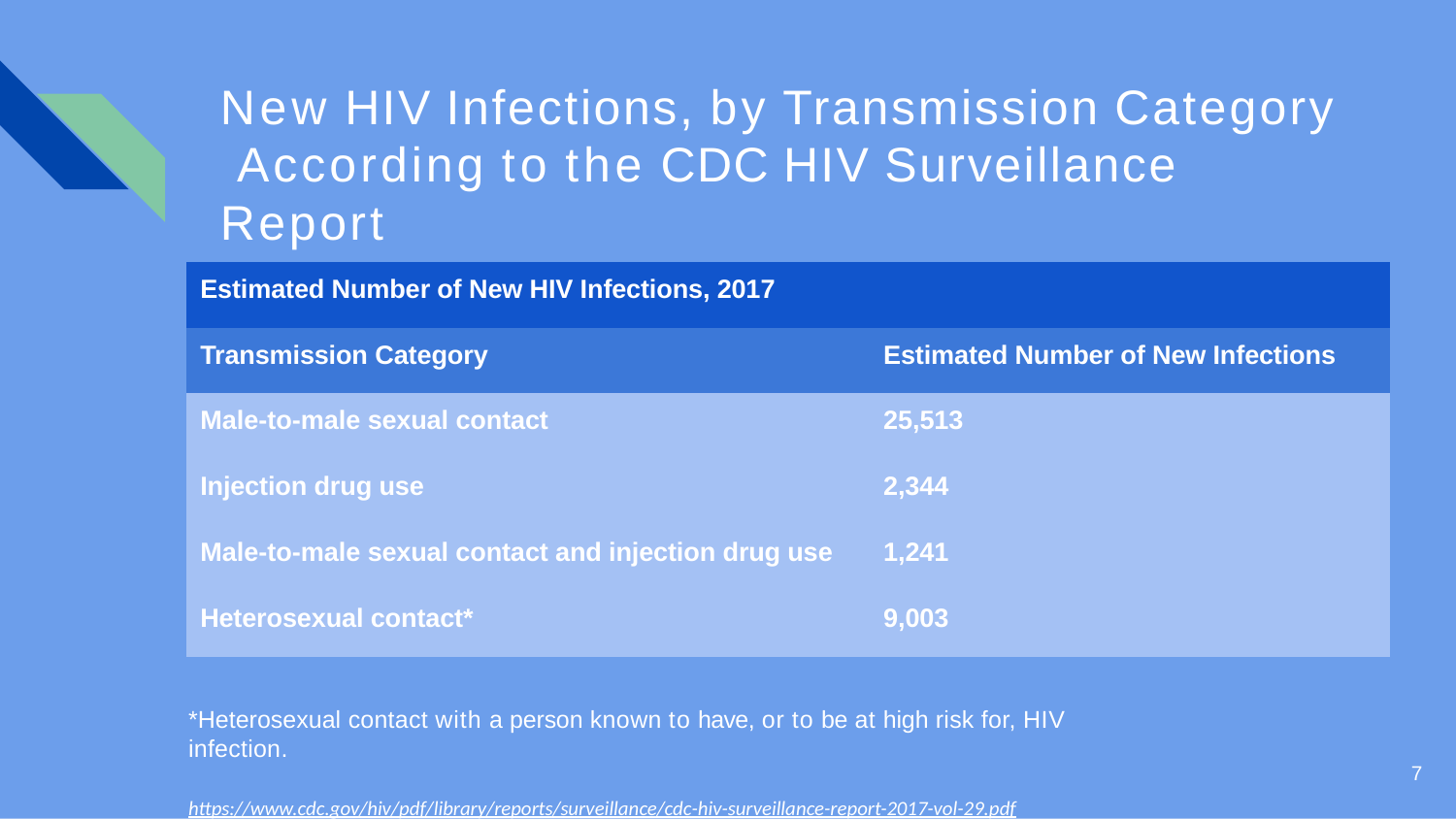

# New HIV Infections, by Transmission Category According to the CDC HIV Surveillance Report
| Estimated Number of New HIV Infections, 2017 | |
| --- | --- |
| Transmission Category | Estimated Number of New Infections |
| Male-to-male sexual contact | 25,513 |
| Injection drug use | 2,344 |
| Male-to-male sexual contact and injection drug use | 1,241 |
| Heterosexual contact\* | 9,003 |
*Heterosexual contact with a person known to have, or to be at high risk for, HIV infection.
https://www.cdc.gov/hiv/pdf/library/reports/surveillance/cdc-hiv-surveillance-report-2017-vol-29.pdf
7

## Slide 9
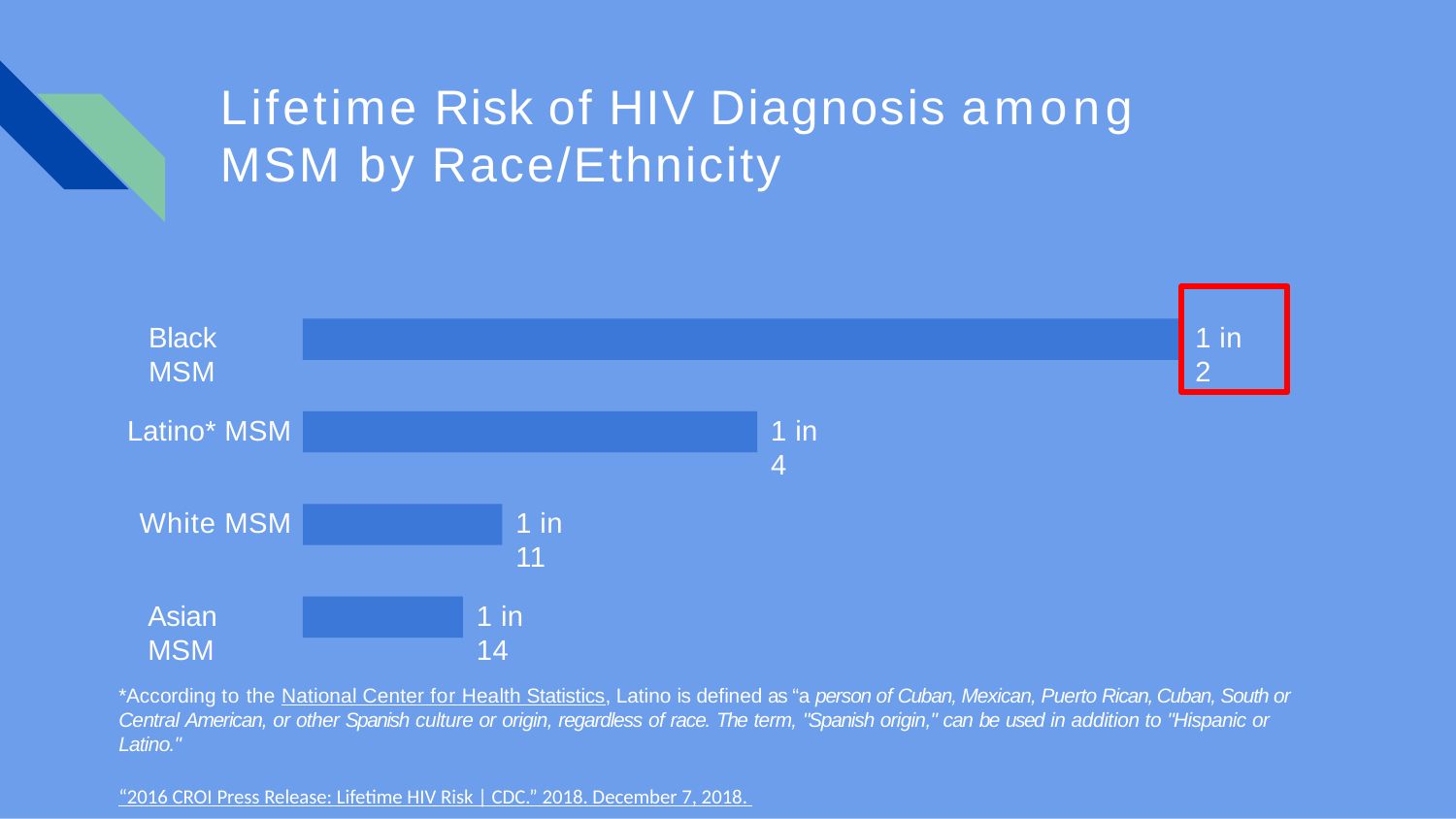

# Lifetime Risk of HIV Diagnosis among MSM by Race/Ethnicity
Black MSM
1 in 2
Latino* MSM
1 in 4
White MSM
1 in 11
Asian MSM
1 in 14
*According to the National Center for Health Statistics, Latino is defined as “a person of Cuban, Mexican, Puerto Rican, Cuban, South or Central American, or other Spanish culture or origin, regardless of race. The term, "Spanish origin," can be used in addition to "Hispanic or Latino."
“2016 CROI Press Release: Lifetime HIV Risk | CDC.” 2018. December 7, 2018.
8

## Slide 10
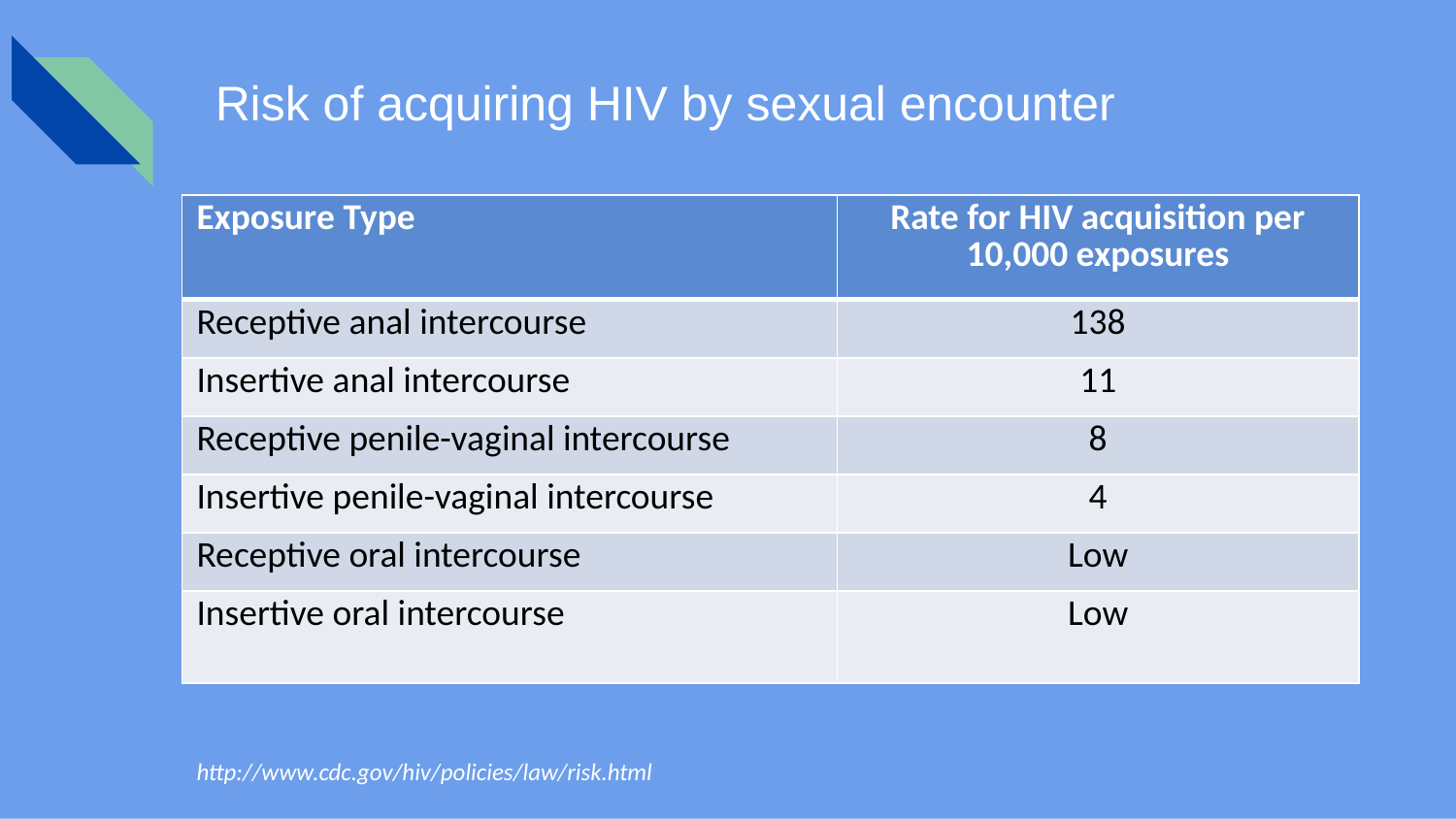

# Risk of acquiring HIV by sexual encounter
| Exposure Type | Rate for HIV acquisition per 10,000 exposures |
| --- | --- |
| Receptive anal intercourse | 138 |
| Insertive anal intercourse | 11 |
| Receptive penile-vaginal intercourse | 8 |
| Insertive penile-vaginal intercourse | 4 |
| Receptive oral intercourse | Low |
| Insertive oral intercourse | Low |
http://www.cdc.gov/hiv/policies/law/risk.html

## Slide 11
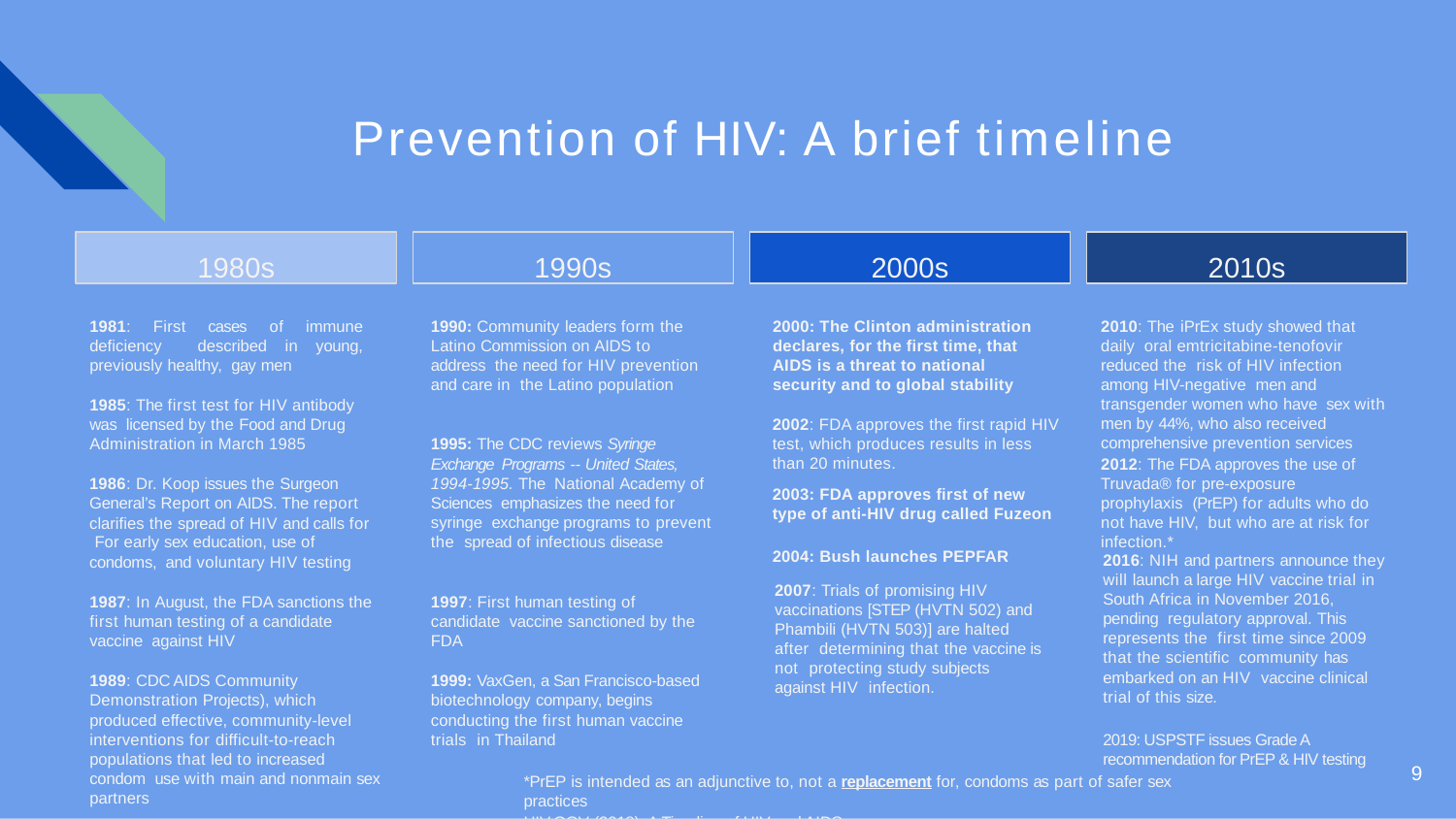

# Prevention of HIV: A brief timeline
1980s
1990s
2000s
2010s
1981: First cases of immune deficiency described in young, previously healthy, gay men
1990: Community leaders form the Latino Commission on AIDS to address the need for HIV prevention and care in the Latino population
2000: The Clinton administration declares, for the first time, that AIDS is a threat to national security and to global stability
2010: The iPrEx study showed that daily oral emtricitabine-tenofovir reduced the risk of HIV infection among HIV-negative men and transgender women who have sex with men by 44%, who also received comprehensive prevention services
1985: The first test for HIV antibody was licensed by the Food and Drug Administration in March 1985
2002: FDA approves the first rapid HIV test, which produces results in less than 20 minutes.
1995: The CDC reviews Syringe Exchange Programs -- United States, 1994-1995. The National Academy of Sciences emphasizes the need for syringe exchange programs to prevent the spread of infectious disease
2012: The FDA approves the use of Truvada® for pre-exposure prophylaxis (PrEP) for adults who do not have HIV, but who are at risk for infection.*
1986: Dr. Koop issues the Surgeon General’s Report on AIDS. The report clarifies the spread of HIV and calls for For early sex education, use of condoms, and voluntary HIV testing
2003: FDA approves first of new type of anti-HIV drug called Fuzeon
2004: Bush launches PEPFAR
2016: NIH and partners announce they will launch a large HIV vaccine trial in South Africa in November 2016, pending regulatory approval. This represents the first time since 2009 that the scientific community has embarked on an HIV vaccine clinical trial of this size.
2019: USPSTF issues Grade A recommendation for PrEP & HIV testing
2007: Trials of promising HIV vaccinations [STEP (HVTN 502) and Phambili (HVTN 503)] are halted after determining that the vaccine is not protecting study subjects against HIV infection.
1987: In August, the FDA sanctions the first human testing of a candidate vaccine against HIV
1997: First human testing of candidate vaccine sanctioned by the FDA
1989: CDC AIDS Community Demonstration Projects), which produced effective, community-level interventions for difficult-to-reach populations that led to increased condom use with main and nonmain sex partners
1999: VaxGen, a San Francisco-based biotechnology company, begins conducting the first human vaccine trials in Thailand
9
*PrEP is intended as an adjunctive to, not a replacement for, condoms as part of safer sex practices
HIV.GOV (2018). A Timeline of HIV and AIDS

## Slide 12
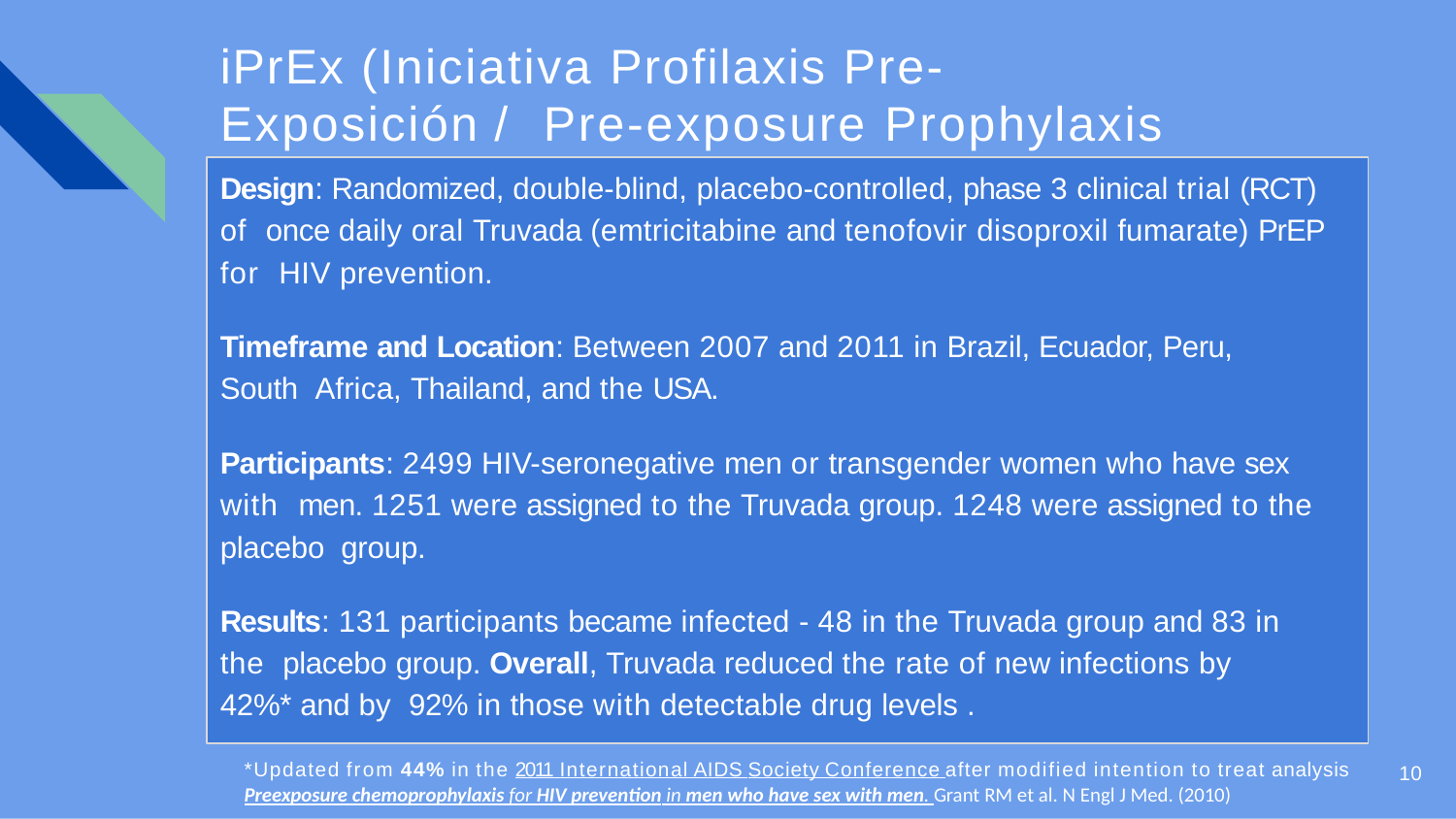

# iPrEx (Iniciativa Profilaxis Pre-Exposición / Pre-exposure Prophylaxis Initiative)
Design: Randomized, double-blind, placebo-controlled, phase 3 clinical trial (RCT) of once daily oral Truvada (emtricitabine and tenofovir disoproxil fumarate) PrEP for HIV prevention.
Timeframe and Location: Between 2007 and 2011 in Brazil, Ecuador, Peru, South Africa, Thailand, and the USA.
Participants: 2499 HIV-seronegative men or transgender women who have sex with men. 1251 were assigned to the Truvada group. 1248 were assigned to the placebo group.
Results: 131 participants became infected - 48 in the Truvada group and 83 in the placebo group. Overall, Truvada reduced the rate of new infections by 42%* and by 92% in those with detectable drug levels .
*Updated from 44% in the 2011 International AIDS Society Conference after modified intention to treat analysis
Preexposure chemoprophylaxis for HIV prevention in men who have sex with men. Grant RM et al. N Engl J Med. (2010)
10

## Slide 13
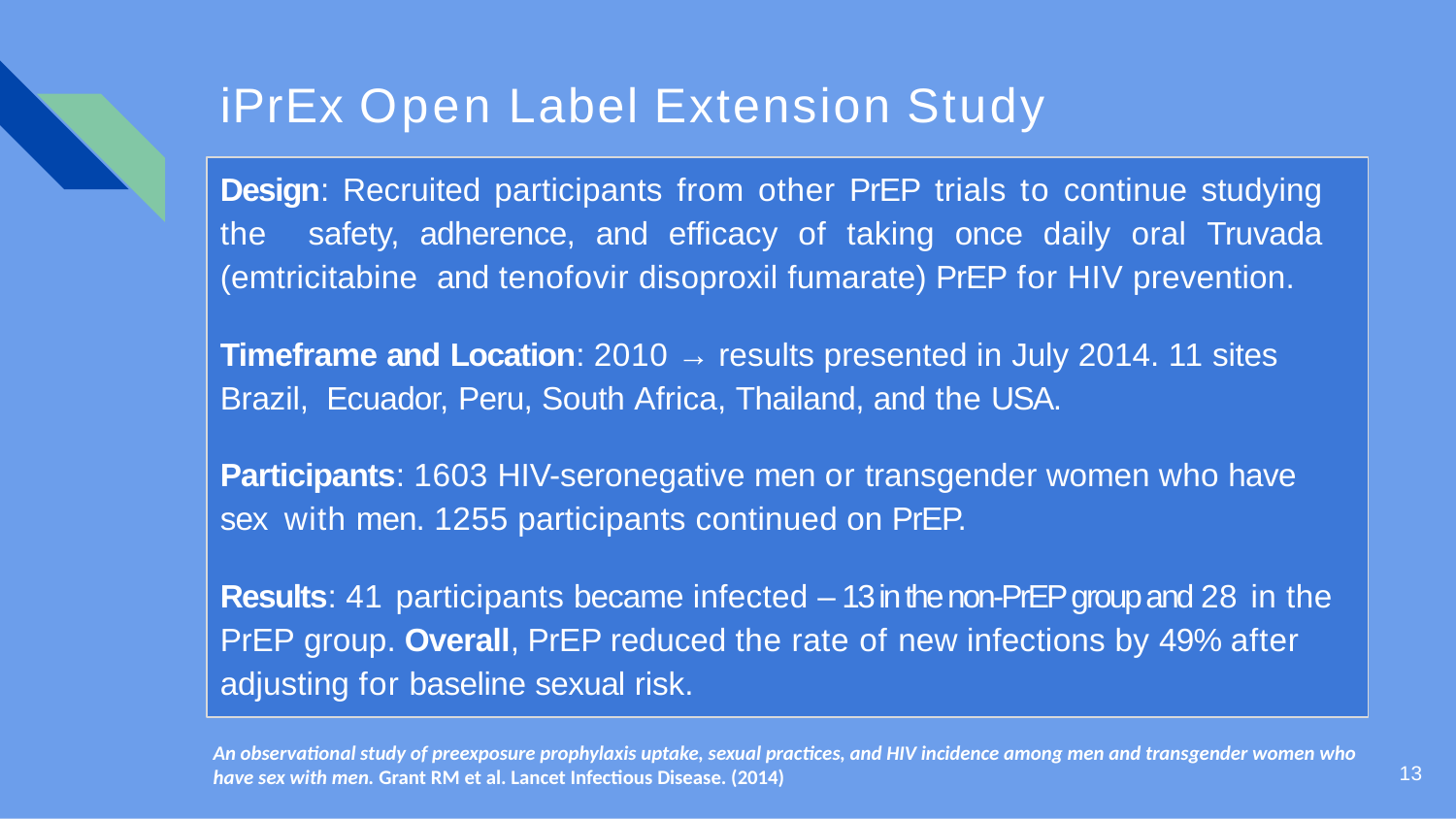

# iPrEx Open Label Extension Study
Design: Recruited participants from other PrEP trials to continue studying the safety, adherence, and efficacy of taking once daily oral Truvada (emtricitabine and tenofovir disoproxil fumarate) PrEP for HIV prevention.
Timeframe and Location: 2010 → results presented in July 2014. 11 sites Brazil, Ecuador, Peru, South Africa, Thailand, and the USA.
Participants: 1603 HIV-seronegative men or transgender women who have sex with men. 1255 participants continued on PrEP.
Results: 41 participants became infected – 13 in the non-PrEP group and 28 in the PrEP group. Overall, PrEP reduced the rate of new infections by 49% after adjusting for baseline sexual risk.
An observational study of preexposure prophylaxis uptake, sexual practices, and HIV incidence among men and transgender women who
have sex with men. Grant RM et al. Lancet Infectious Disease. (2014)
13

## Slide 14
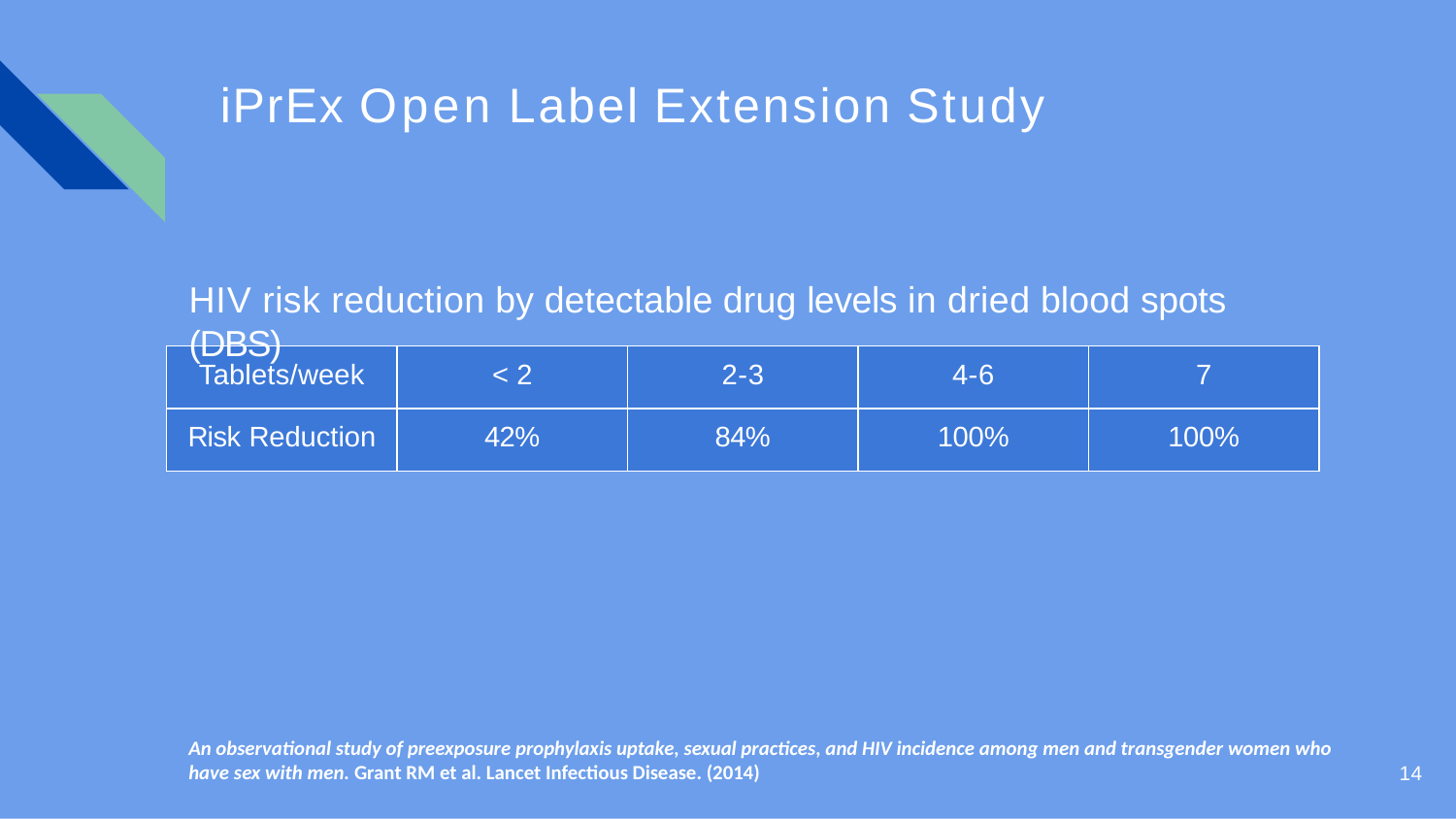

# iPrEx Open Label Extension Study
HIV risk reduction by detectable drug levels in dried blood spots (DBS)
| Tablets/week | < 2 | 2-3 | 4-6 | 7 |
| --- | --- | --- | --- | --- |
| Risk Reduction | 42% | 84% | 100% | 100% |
An observational study of preexposure prophylaxis uptake, sexual practices, and HIV incidence among men and transgender women who
have sex with men. Grant RM et al. Lancet Infectious Disease. (2014)
14

## Slide 15
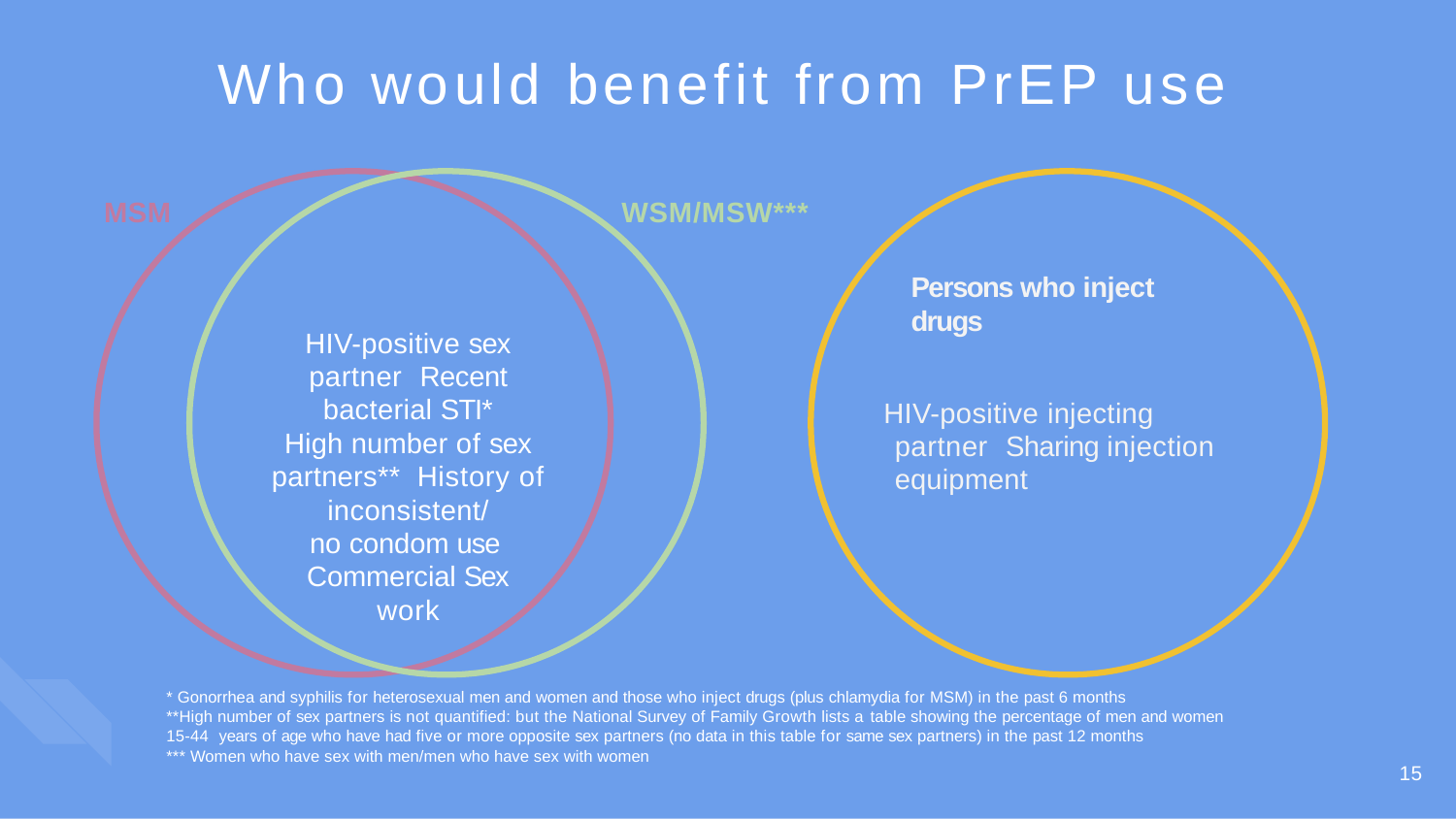

# Who would benefit from PrEP use
MSM
WSM/MSW***
Persons who inject drugs
HIV-positive sex partner Recent bacterial STI*
High number of sex partners** History of inconsistent/
no condom use Commercial Sex work
HIV-positive injecting partner Sharing injection equipment
* Gonorrhea and syphilis for heterosexual men and women and those who inject drugs (plus chlamydia for MSM) in the past 6 months
**High number of sex partners is not quantified: but the National Survey of Family Growth lists a table showing the percentage of men and women 15-44 years of age who have had five or more opposite sex partners (no data in this table for same sex partners) in the past 12 months
*** Women who have sex with men/men who have sex with women
15

## Slide 16
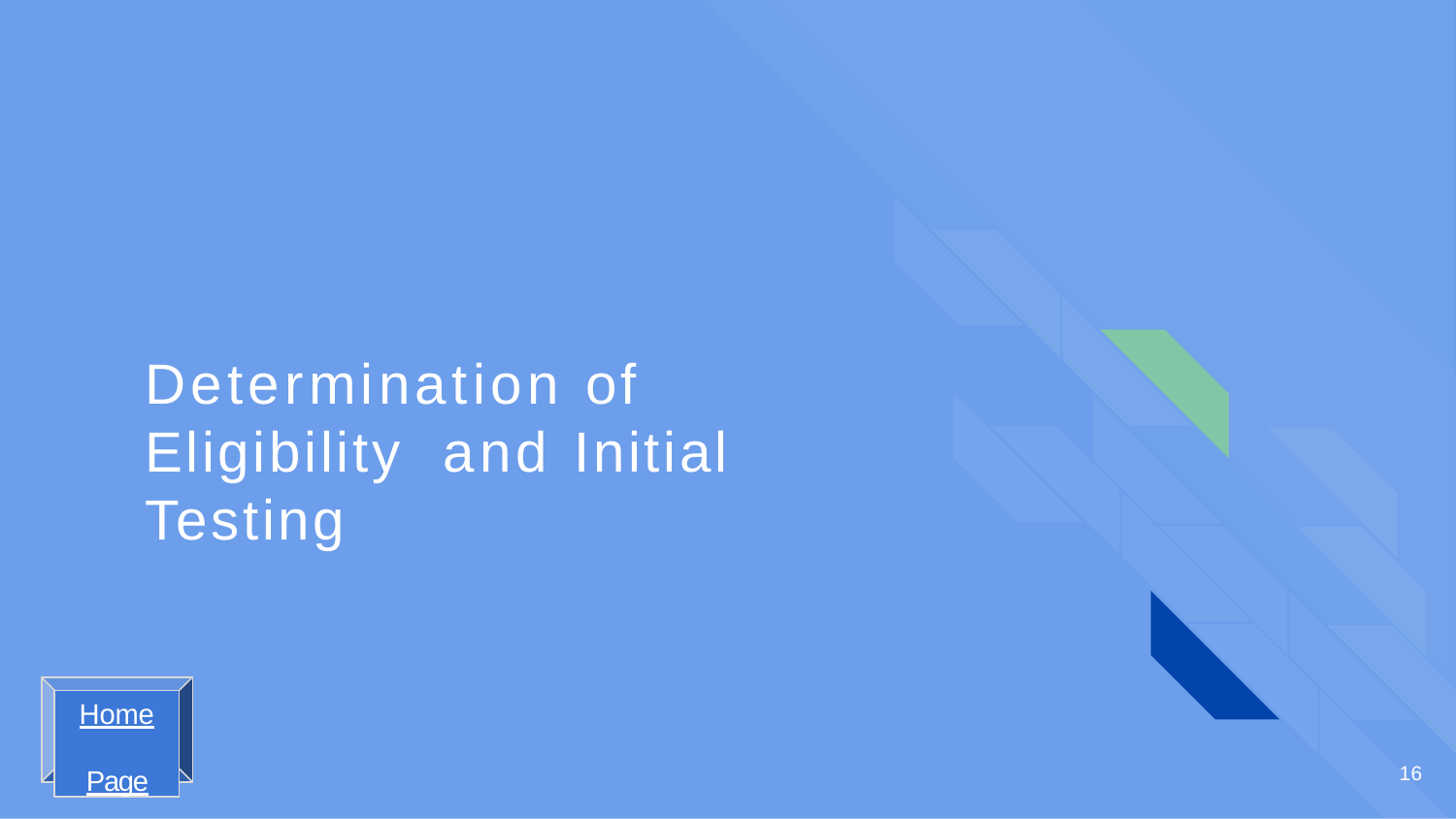

# Determination of Eligibility and Initial Testing
Home Page
16

## Slide 17
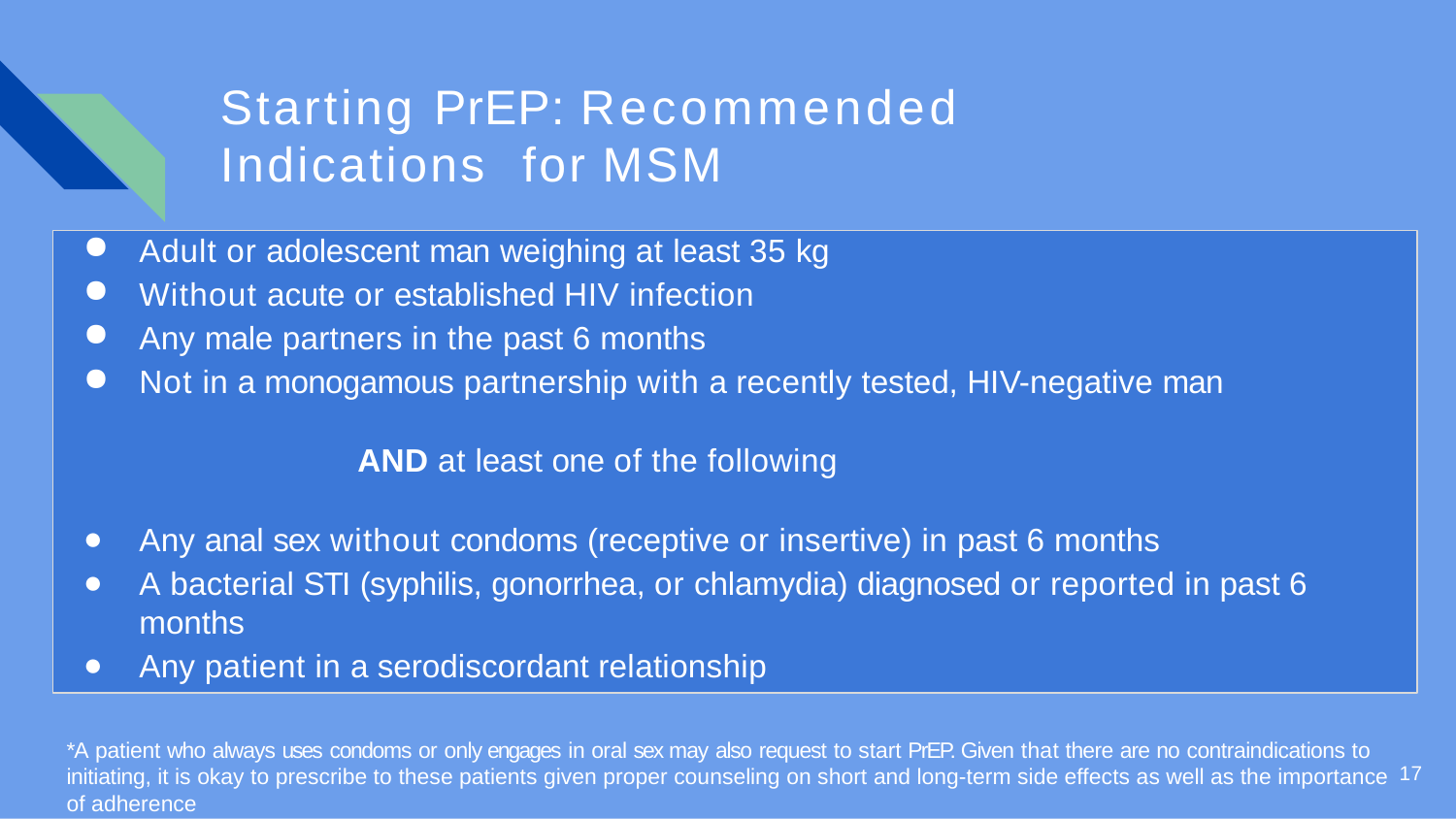

# Starting PrEP: Recommended Indications for MSM
Adult or adolescent man weighing at least 35 kg
Without acute or established HIV infection
Any male partners in the past 6 months
Not in a monogamous partnership with a recently tested, HIV-negative man
AND at least one of the following
Any anal sex without condoms (receptive or insertive) in past 6 months
A bacterial STI (syphilis, gonorrhea, or chlamydia) diagnosed or reported in past 6 months
Any patient in a serodiscordant relationship
*A patient who always uses condoms or only engages in oral sex may also request to start PrEP. Given that there are no contraindications to initiating, it is okay to prescribe to these patients given proper counseling on short and long-term side effects as well as the importance of adherence
https://www.cdc.gov/hiv/basics/prep.html
17

## Slide 18
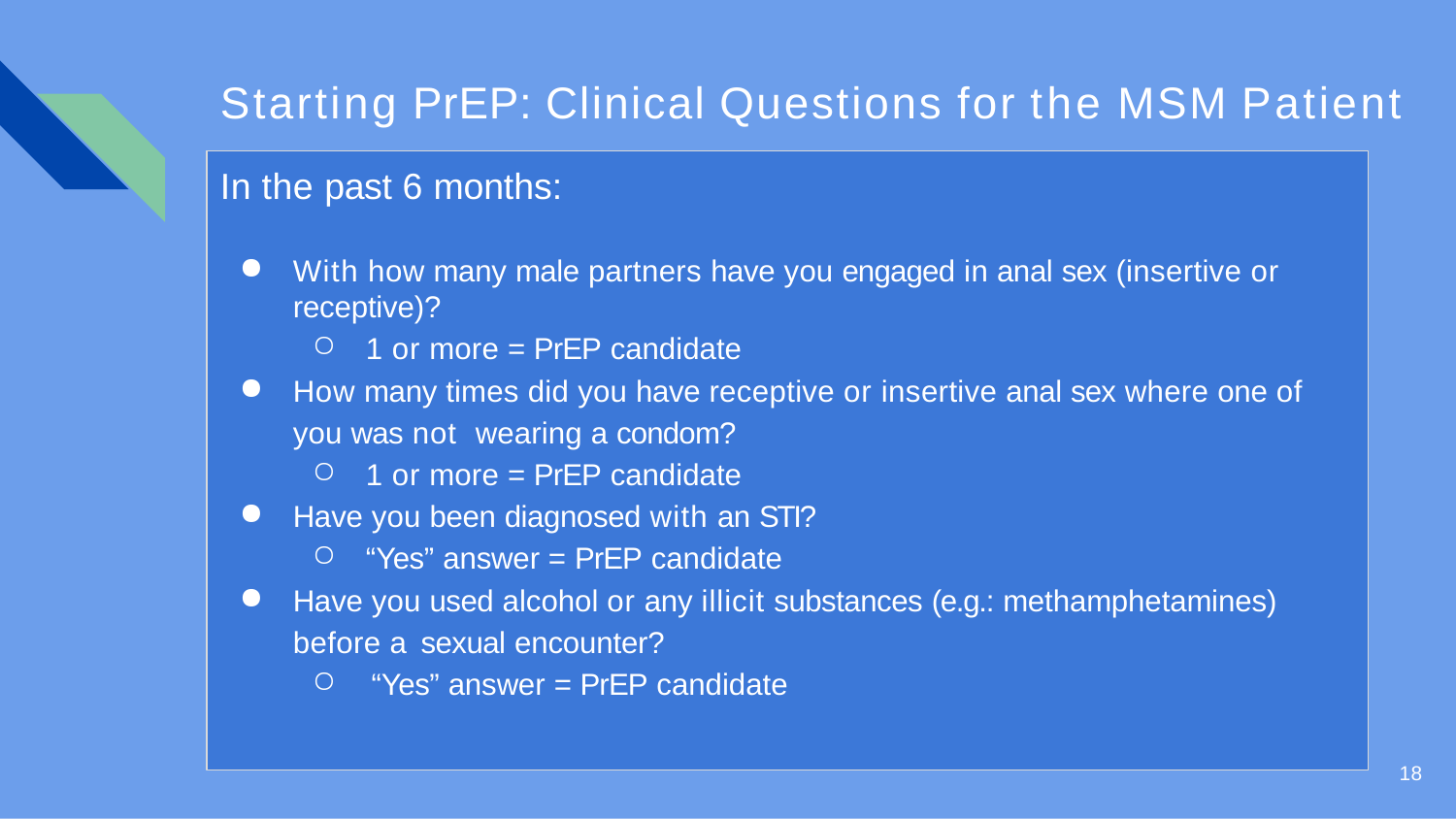

# Starting PrEP: Clinical Questions for the MSM Patient
In the past 6 months:
With how many male partners have you engaged in anal sex (insertive or receptive)?
1 or more = PrEP candidate
How many times did you have receptive or insertive anal sex where one of you was not wearing a condom?
1 or more = PrEP candidate
Have you been diagnosed with an STI?
“Yes” answer = PrEP candidate
Have you used alcohol or any illicit substances (e.g.: methamphetamines) before a sexual encounter?
“Yes” answer = PrEP candidate
18

## Slide 19
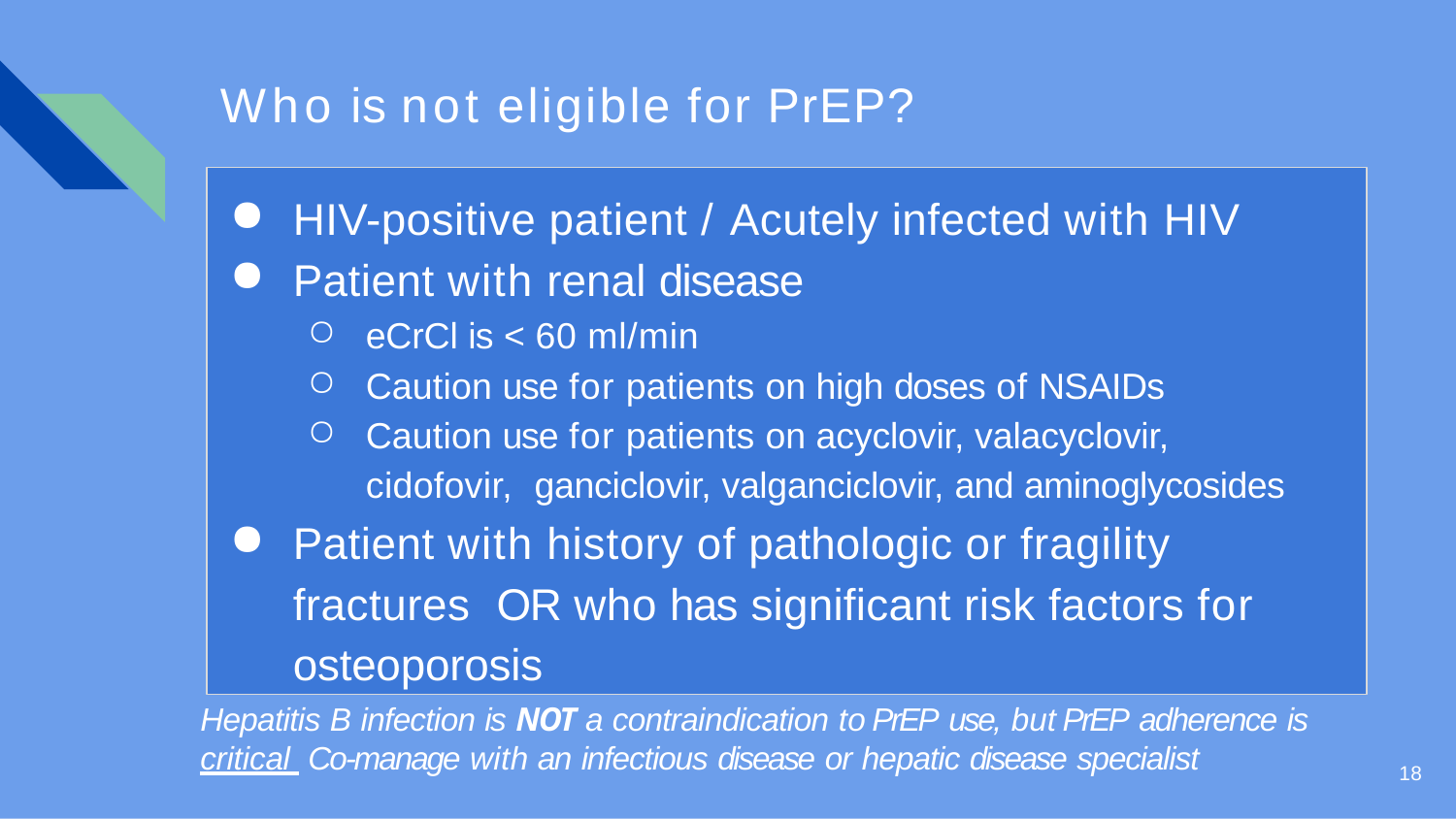

# Who is not eligible for PrEP?
HIV-positive patient / Acutely infected with HIV
Patient with renal disease
eCrCl is < 60 ml/min
Caution use for patients on high doses of NSAIDs
Caution use for patients on acyclovir, valacyclovir, cidofovir, ganciclovir, valganciclovir, and aminoglycosides
Patient with history of pathologic or fragility fractures OR who has significant risk factors for osteoporosis
Hepatitis B infection is NOT a contraindication to PrEP use, but PrEP adherence is critical Co-manage with an infectious disease or hepatic disease specialist
18

## Slide 20
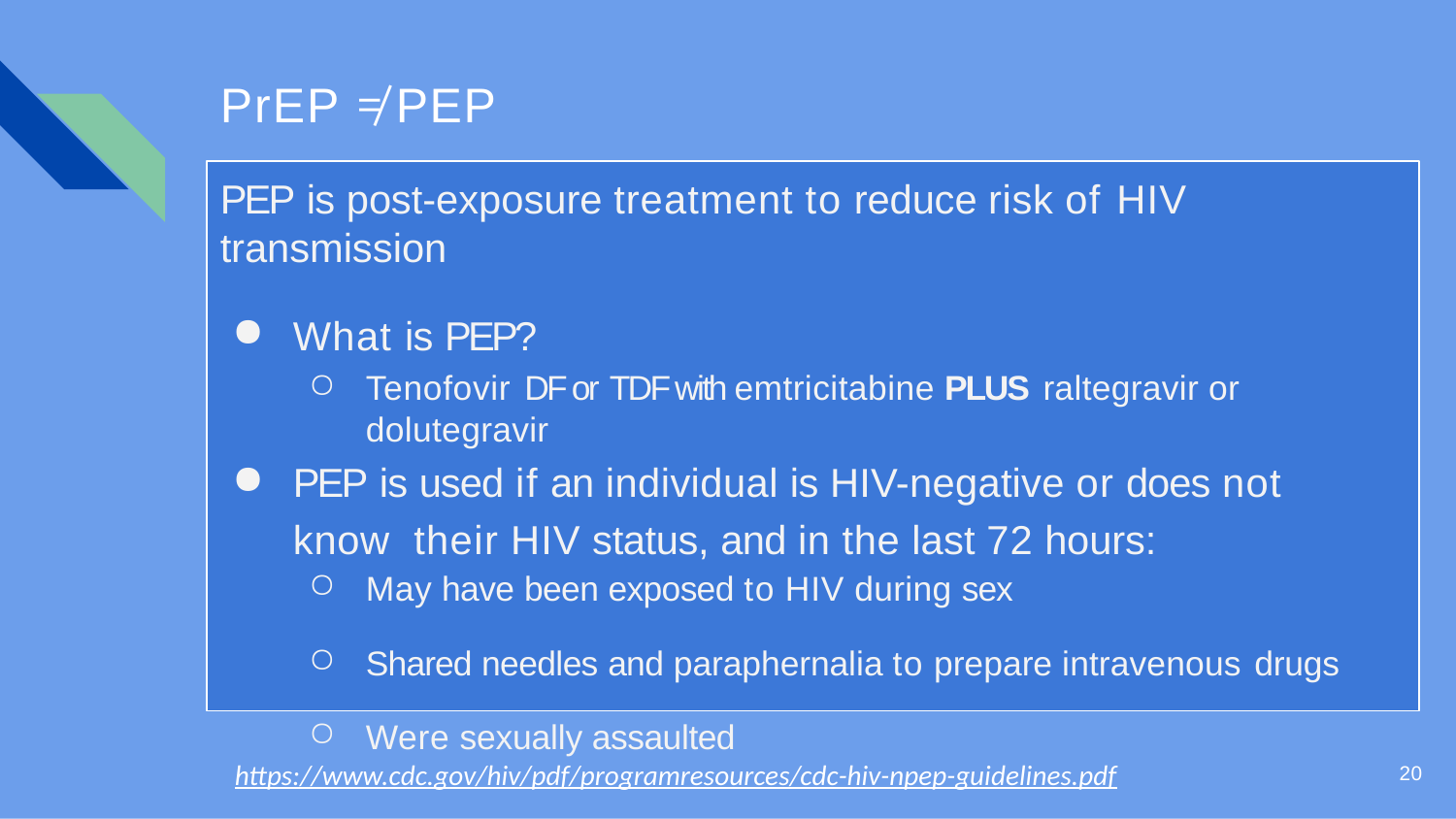

# PrEP ≠ PEP
PEP is post-exposure treatment to reduce risk of HIV transmission
What is PEP?
Tenofovir DF or TDF with emtricitabine PLUS raltegravir or dolutegravir
PEP is used if an individual is HIV-negative or does not know their HIV status, and in the last 72 hours:
May have been exposed to HIV during sex
Shared needles and paraphernalia to prepare intravenous drugs
Were sexually assaulted
https://www.cdc.gov/hiv/pdf/programresources/cdc-hiv-npep-guidelines.pdf
20

## Slide 21
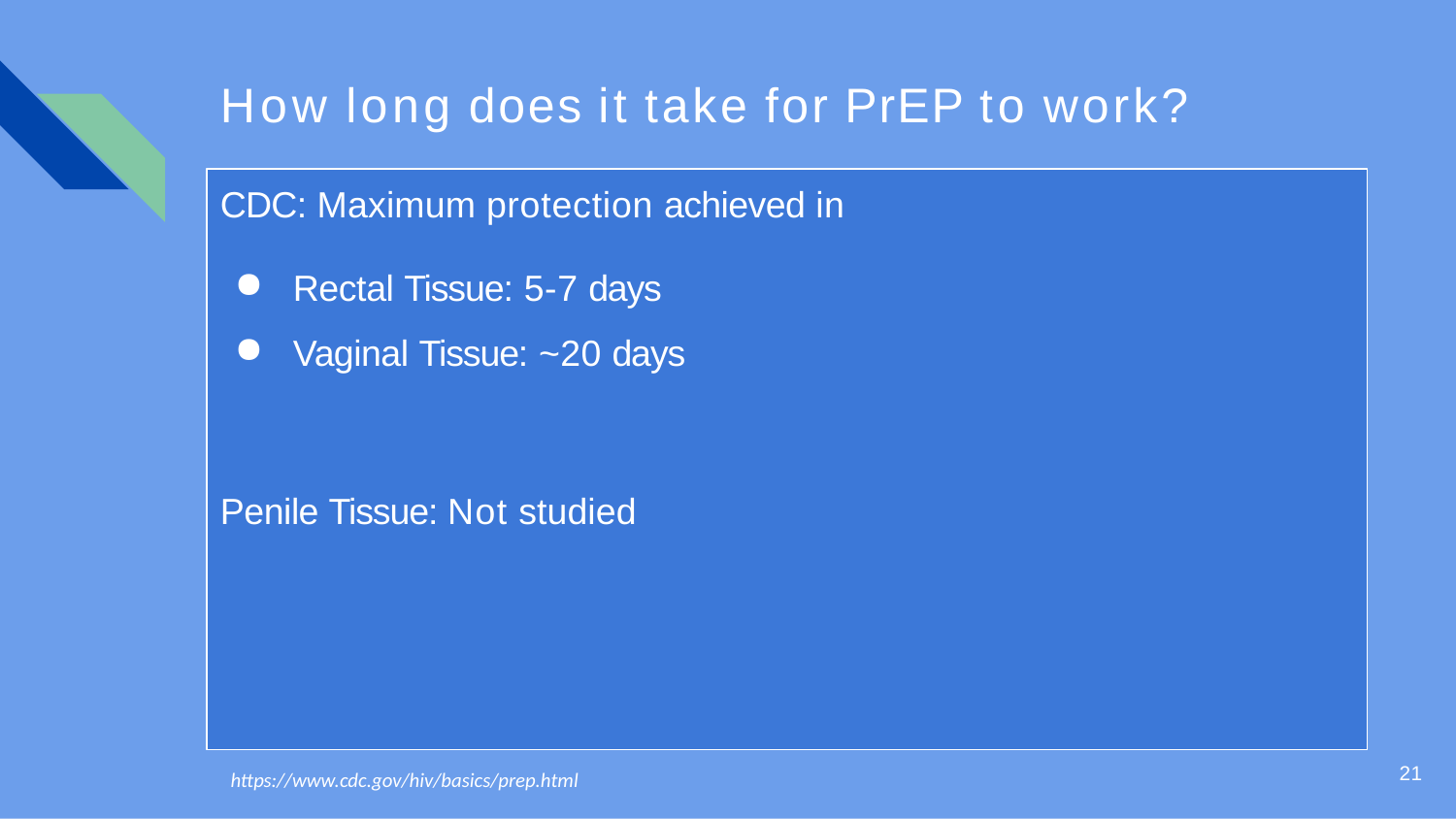

# How long does it take for PrEP to work?
CDC: Maximum protection achieved in
Rectal Tissue: 5-7 days
Vaginal Tissue: ~20 days
Penile Tissue: Not studied
21
https://www.cdc.gov/hiv/basics/prep.html

## Slide 22
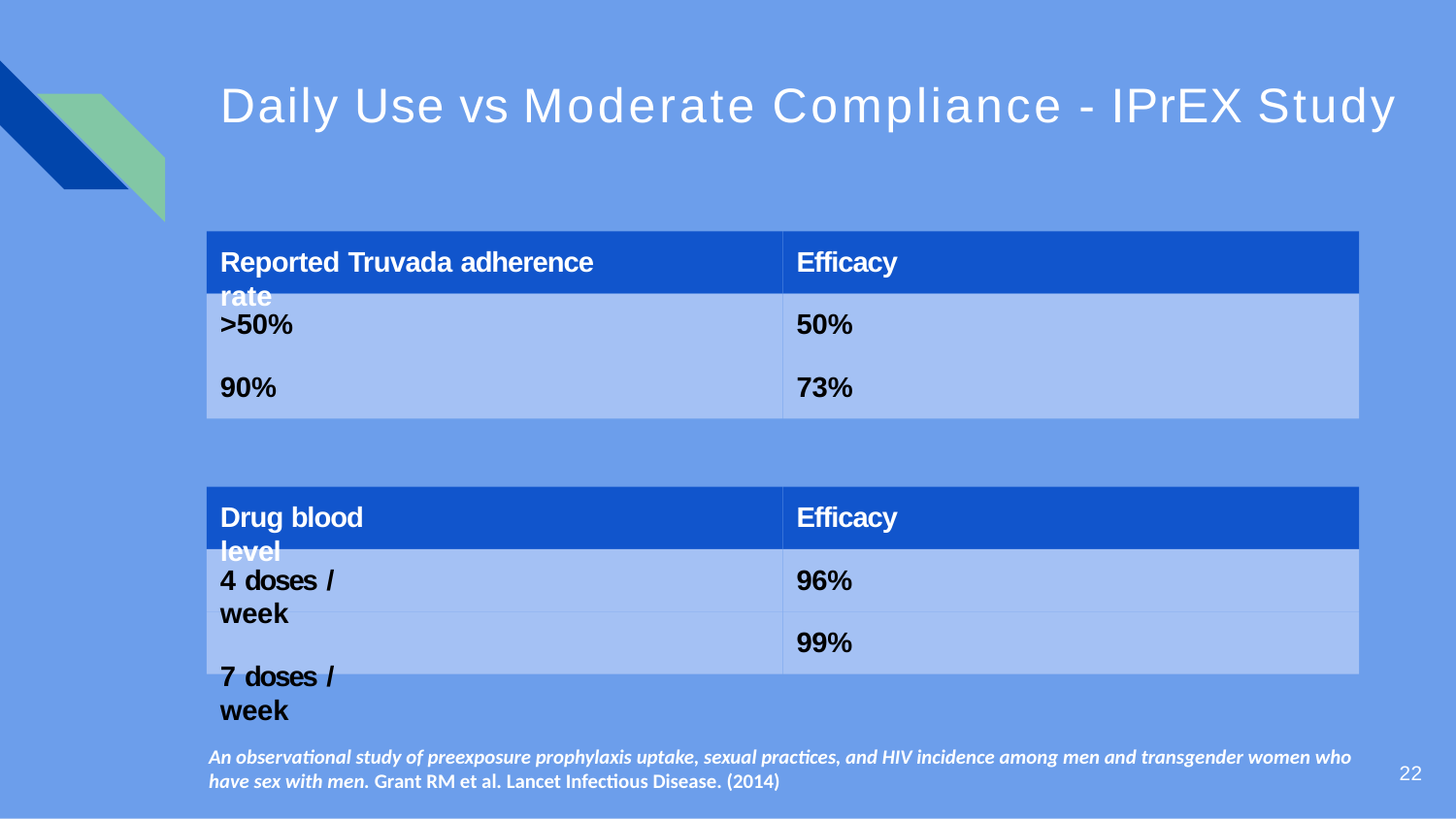

# Daily Use vs Moderate Compliance - IPrEX Study
Reported Truvada adherence rate
Efficacy
>50%
50%
90%
73%
Drug blood level
Efficacy
4 doses / week
7 doses / week
96%
99%
An observational study of preexposure prophylaxis uptake, sexual practices, and HIV incidence among men and transgender women who
have sex with men. Grant RM et al. Lancet Infectious Disease. (2014)
22

## Slide 23
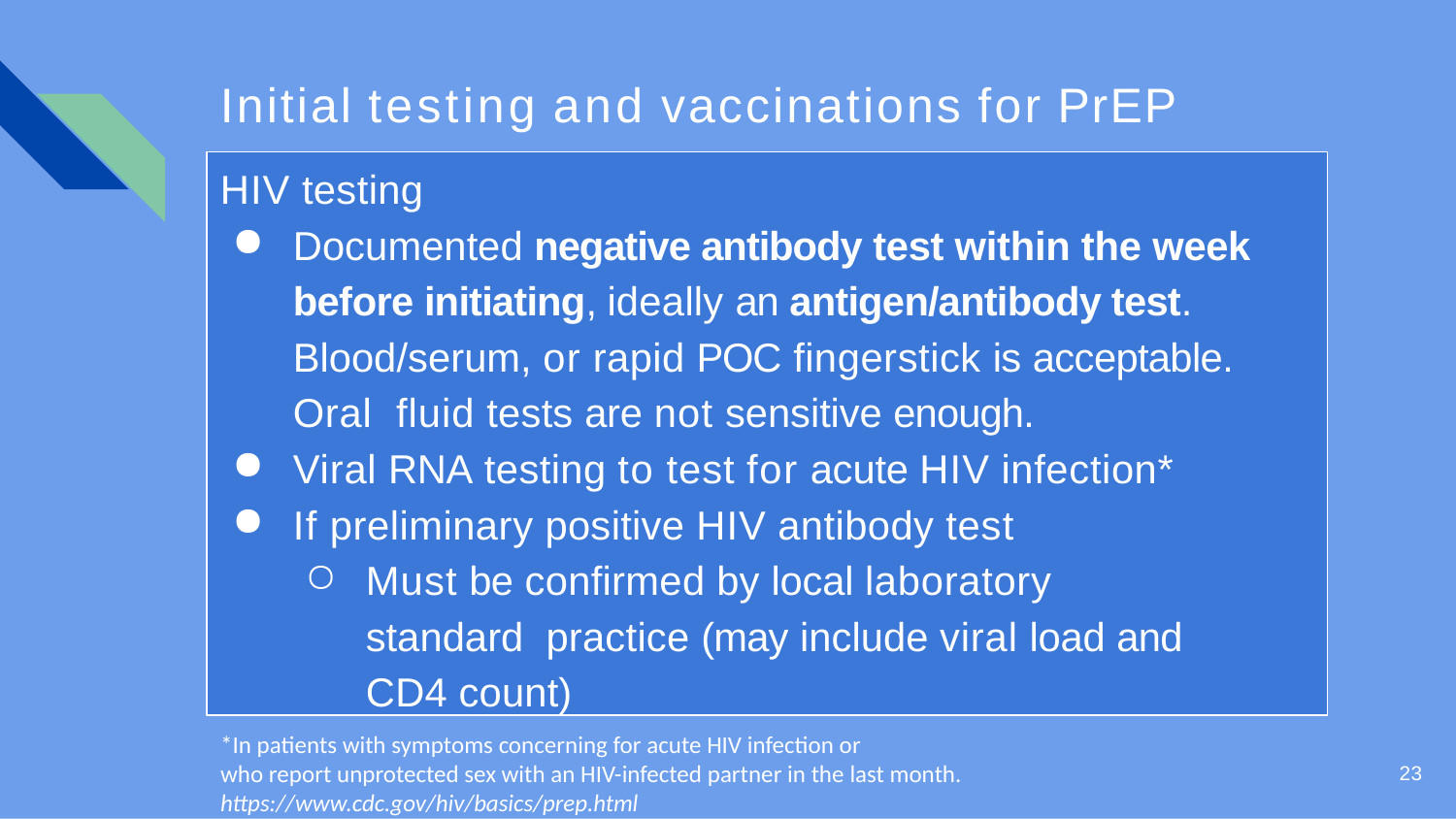

# Initial testing and vaccinations for PrEP
HIV testing
Documented negative antibody test within the week before initiating, ideally an antigen/antibody test. Blood/serum, or rapid POC fingerstick is acceptable. Oral fluid tests are not sensitive enough.
Viral RNA testing to test for acute HIV infection*
If preliminary positive HIV antibody test
Must be confirmed by local laboratory standard practice (may include viral load and CD4 count)
*In patients with symptoms concerning for acute HIV infection or
who report unprotected sex with an HIV-infected partner in the last month.
https://www.cdc.gov/hiv/basics/prep.html
23

## Slide 24
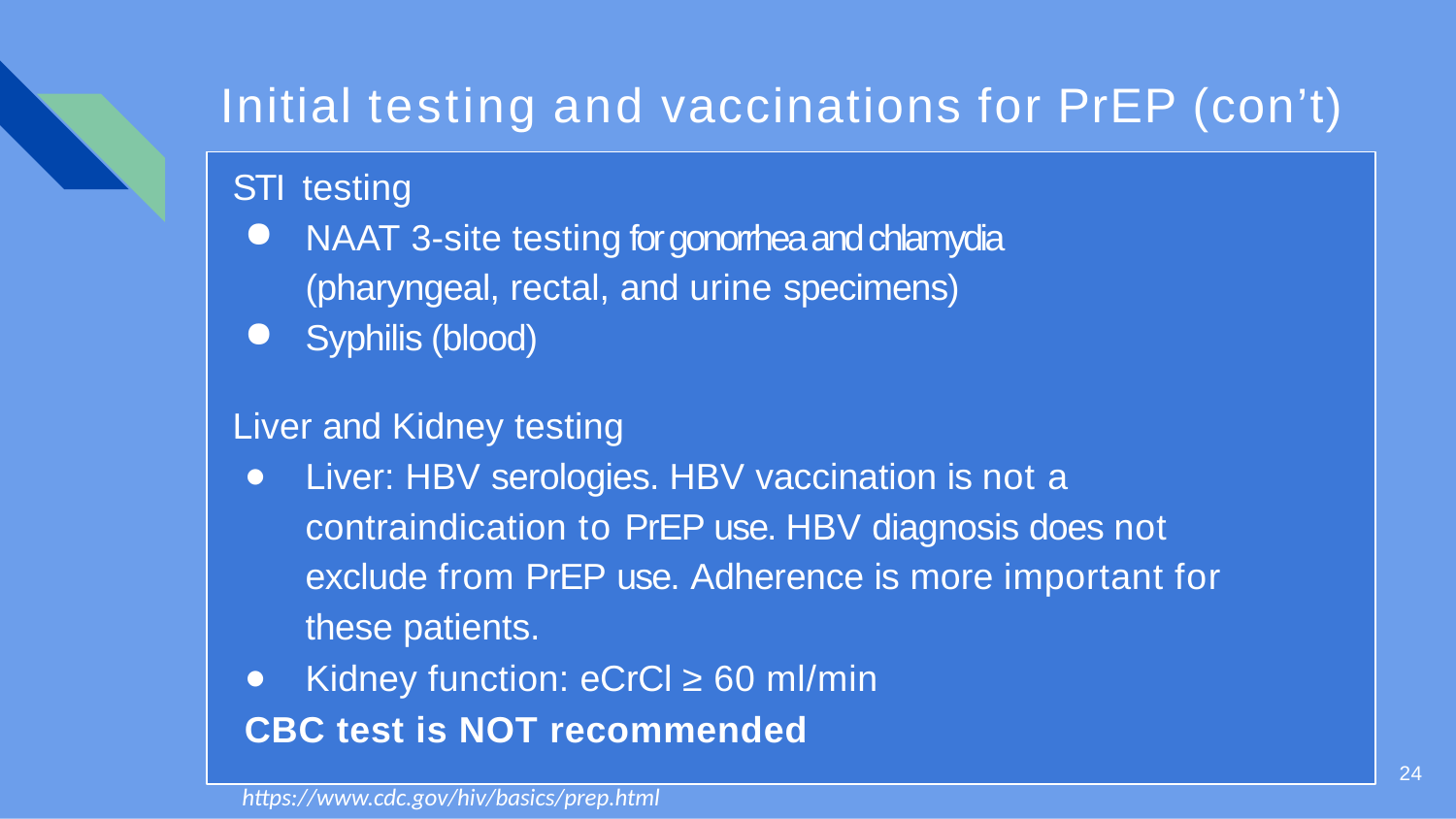

# Initial testing and vaccinations for PrEP (con’t)
STI testing
NAAT 3-site testing for gonorrhea and chlamydia (pharyngeal, rectal, and urine specimens)
Syphilis (blood)
Liver and Kidney testing
Liver: HBV serologies. HBV vaccination is not a contraindication to PrEP use. HBV diagnosis does not exclude from PrEP use. Adherence is more important for these patients.
Kidney function: eCrCl ≥ 60 ml/min
CBC test is NOT recommended
24
https://www.cdc.gov/hiv/basics/prep.html

## Slide 25
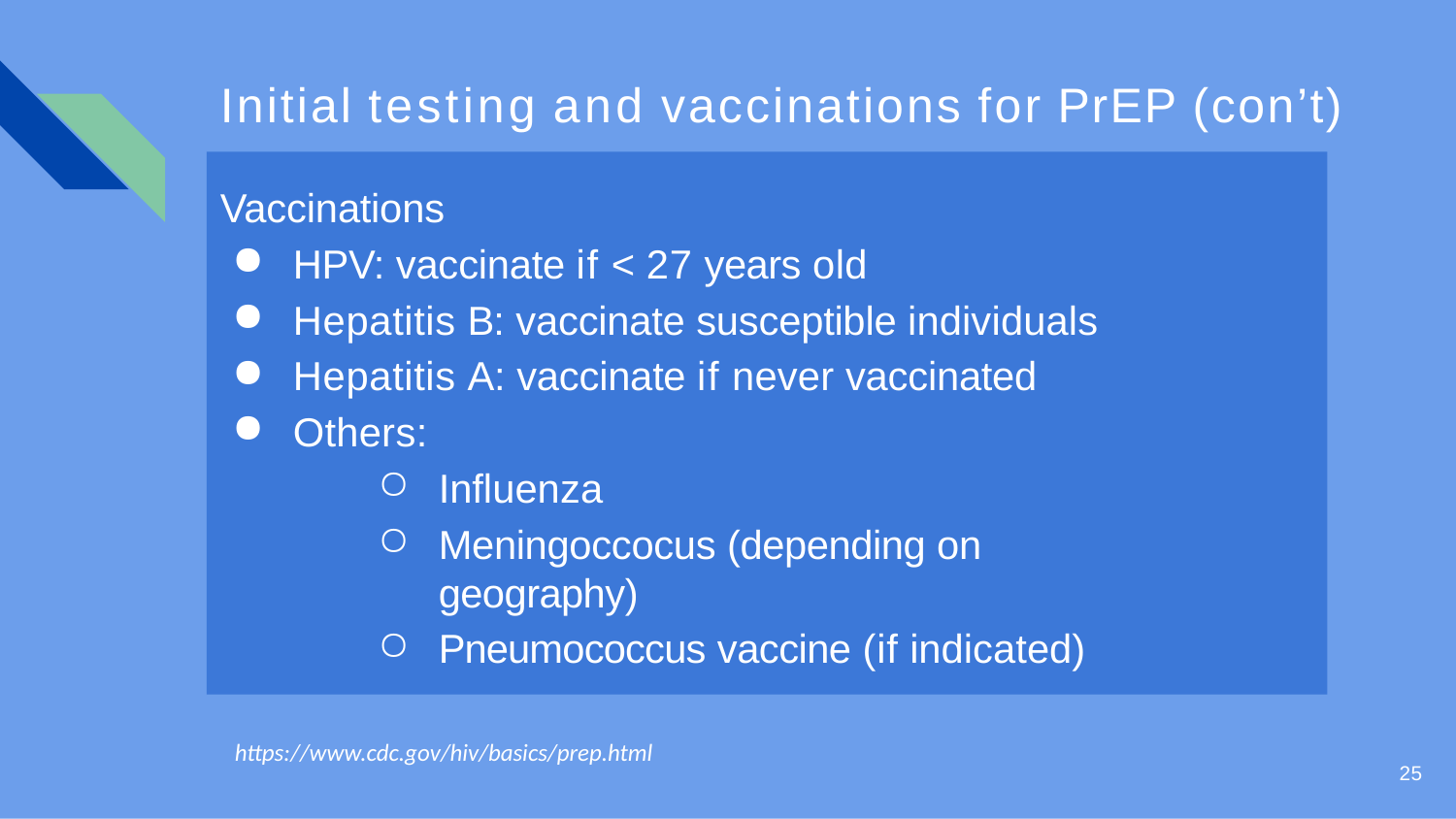

# Initial testing and vaccinations for PrEP (con’t)
Vaccinations
HPV: vaccinate if < 27 years old
Hepatitis B: vaccinate susceptible individuals
Hepatitis A: vaccinate if never vaccinated
Others:
Influenza
Meningoccocus (depending on geography)
Pneumococcus vaccine (if indicated)
https://www.cdc.gov/hiv/basics/prep.html
25

## Slide 26
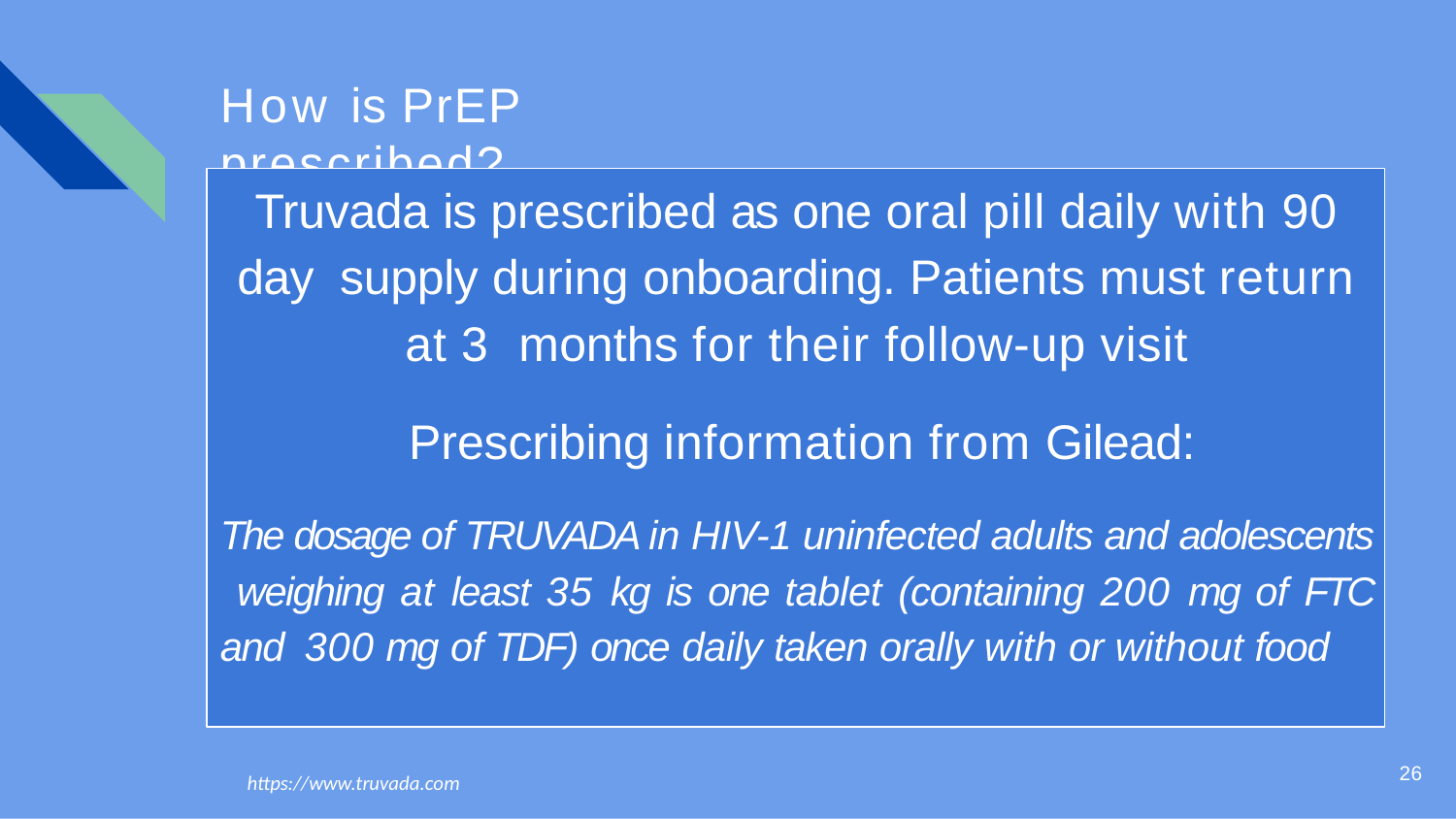

# How is PrEP prescribed?
Truvada is prescribed as one oral pill daily with 90 day supply during onboarding. Patients must return at 3 months for their follow-up visit
Prescribing information from Gilead:
The dosage of TRUVADA in HIV-1 uninfected adults and adolescents weighing at least 35 kg is one tablet (containing 200 mg of FTC and 300 mg of TDF) once daily taken orally with or without food
26
https://www.truvada.com

## Slide 27
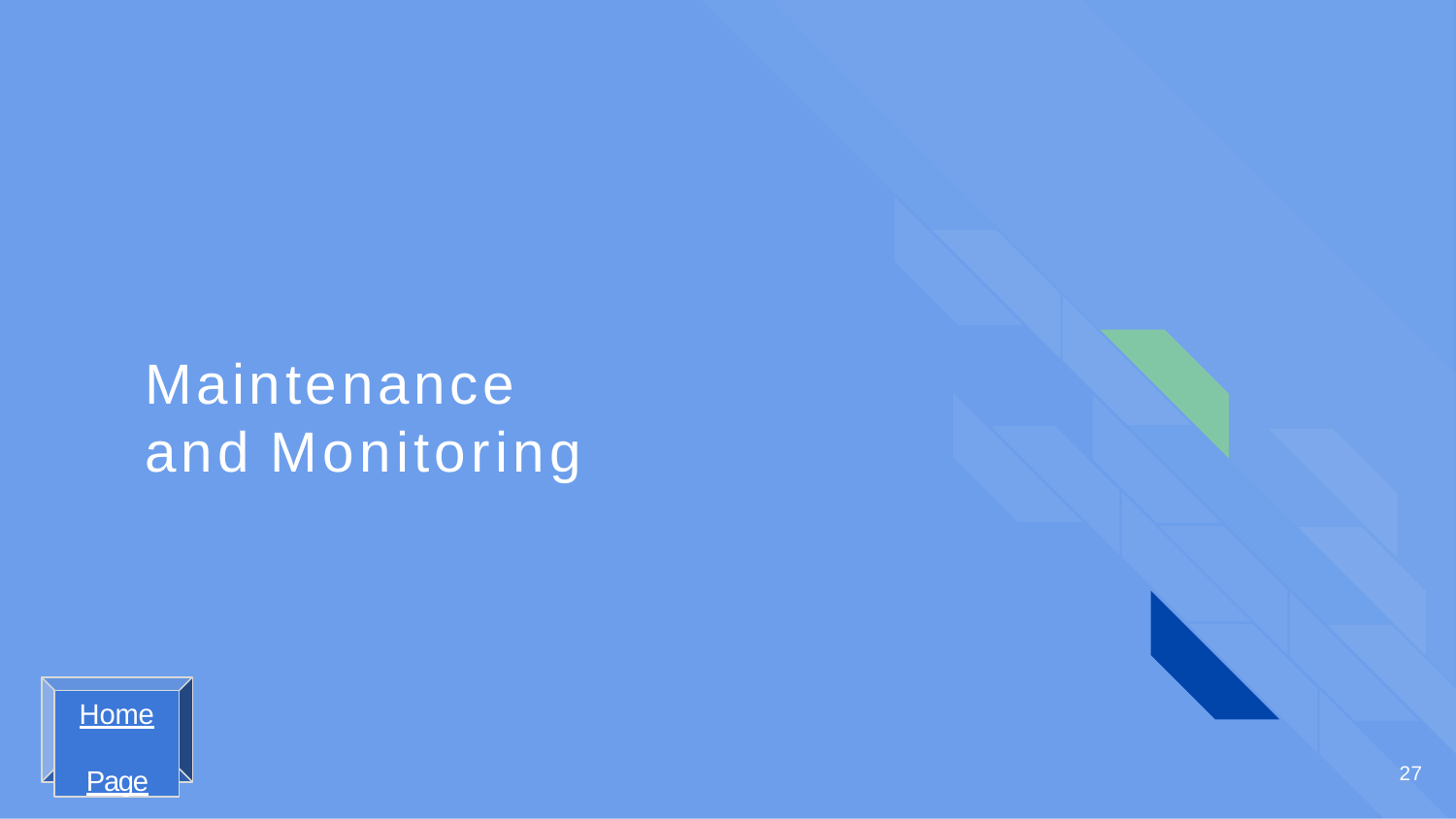

# Maintenance and Monitoring
Home Page
27

## Slide 28
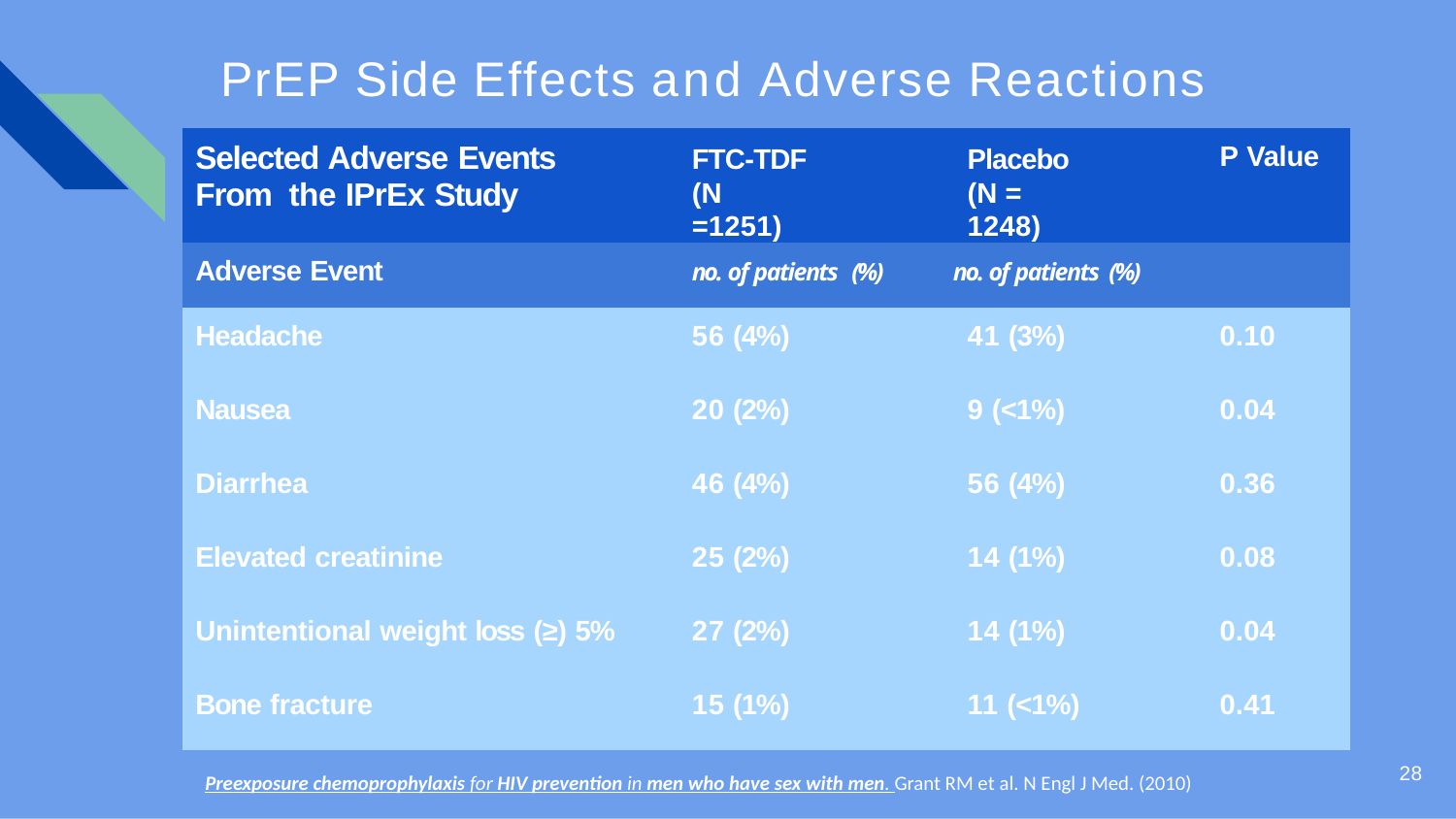

# PrEP Side Effects and Adverse Reactions
| Selected Adverse Events From the IPrEx Study | FTC-TDF (N =1251) | Placebo (N = 1248) | P Value |
| --- | --- | --- | --- |
| Adverse Event | no. of patients (%) no. of patients (%) | | |
| Headache | 56 (4%) | 41 (3%) | 0.10 |
| Nausea | 20 (2%) | 9 (<1%) | 0.04 |
| Diarrhea | 46 (4%) | 56 (4%) | 0.36 |
| Elevated creatinine | 25 (2%) | 14 (1%) | 0.08 |
| Unintentional weight loss (≥) 5% | 27 (2%) | 14 (1%) | 0.04 |
| Bone fracture | 15 (1%) | 11 (<1%) | 0.41 |
28
Preexposure chemoprophylaxis for HIV prevention in men who have sex with men. Grant RM et al. N Engl J Med. (2010)

## Slide 29
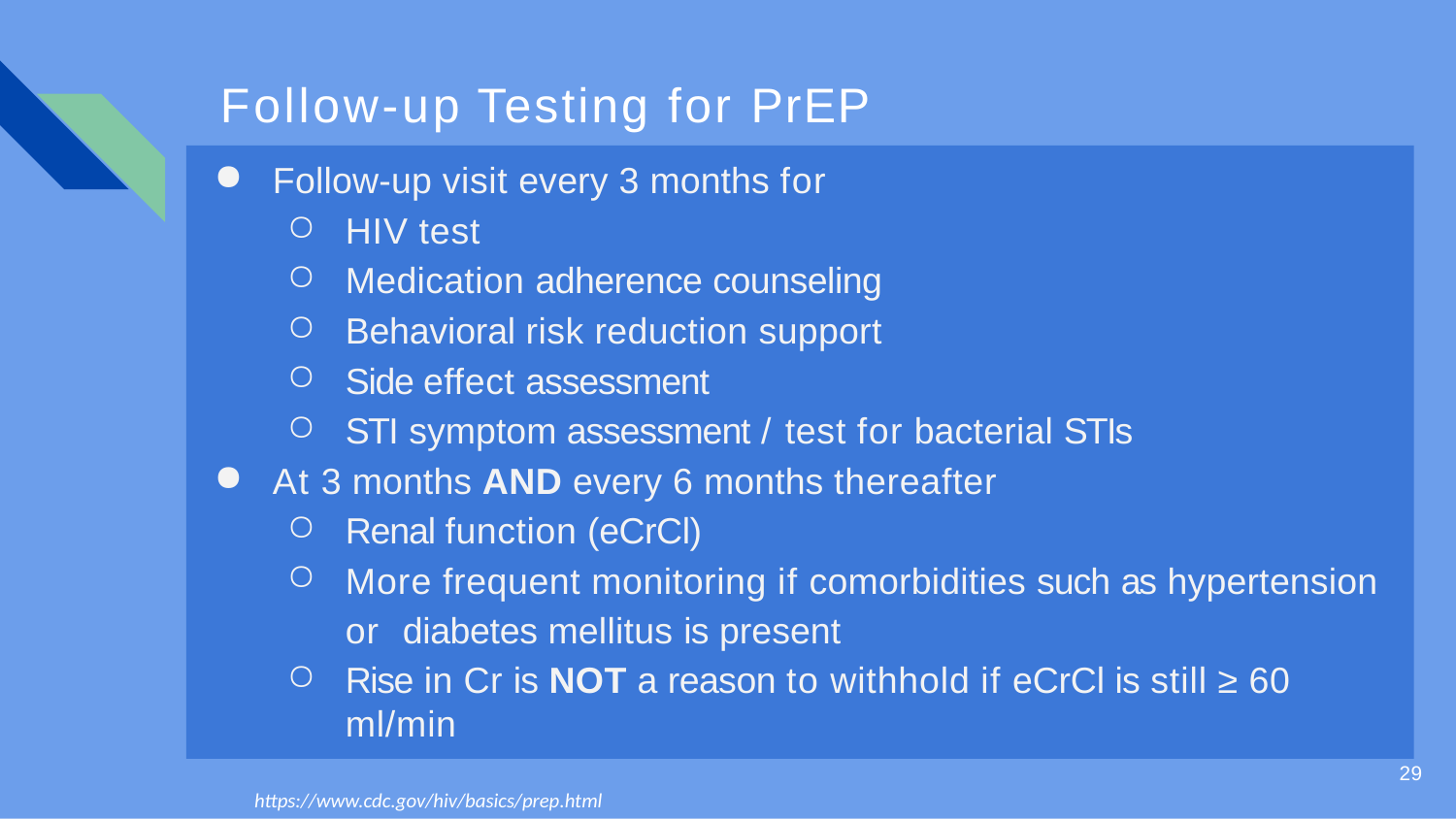

# Follow-up Testing for PrEP
Follow-up visit every 3 months for
HIV test
Medication adherence counseling
Behavioral risk reduction support
Side effect assessment
STI symptom assessment / test for bacterial STIs
At 3 months AND every 6 months thereafter
Renal function (eCrCl)
More frequent monitoring if comorbidities such as hypertension or diabetes mellitus is present
Rise in Cr is NOT a reason to withhold if eCrCl is still ≥ 60 ml/min
29
https://www.cdc.gov/hiv/basics/prep.html

## Slide 30
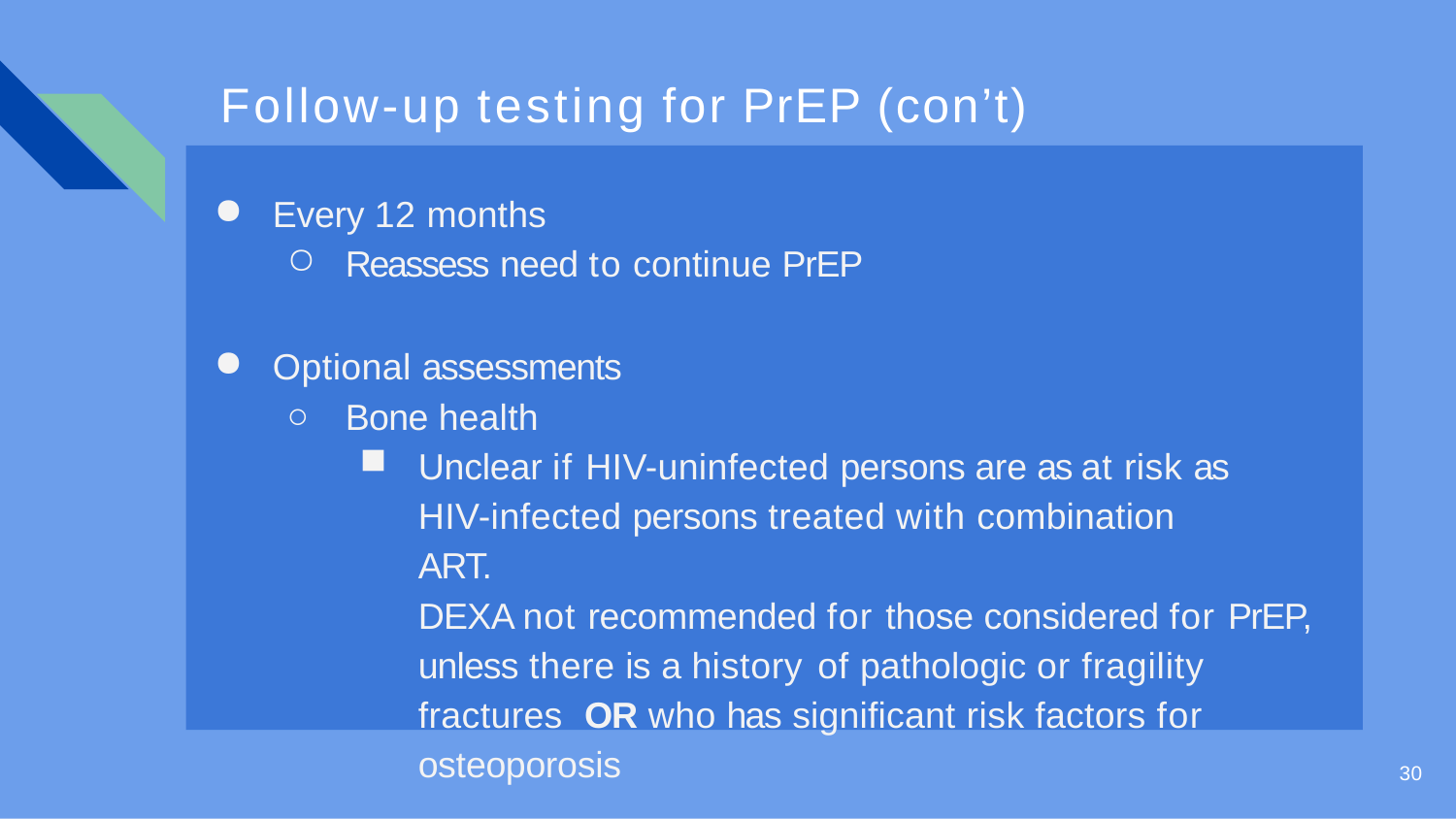

# Follow-up testing for PrEP (con’t)
Every 12 months
Reassess need to continue PrEP
Optional assessments
Bone health
Unclear if HIV-uninfected persons are as at risk as HIV-infected persons treated with combination ART.
DEXA not recommended for those considered for PrEP, unless there is a history of pathologic or fragility fractures OR who has significant risk factors for osteoporosis
30

## Slide 31
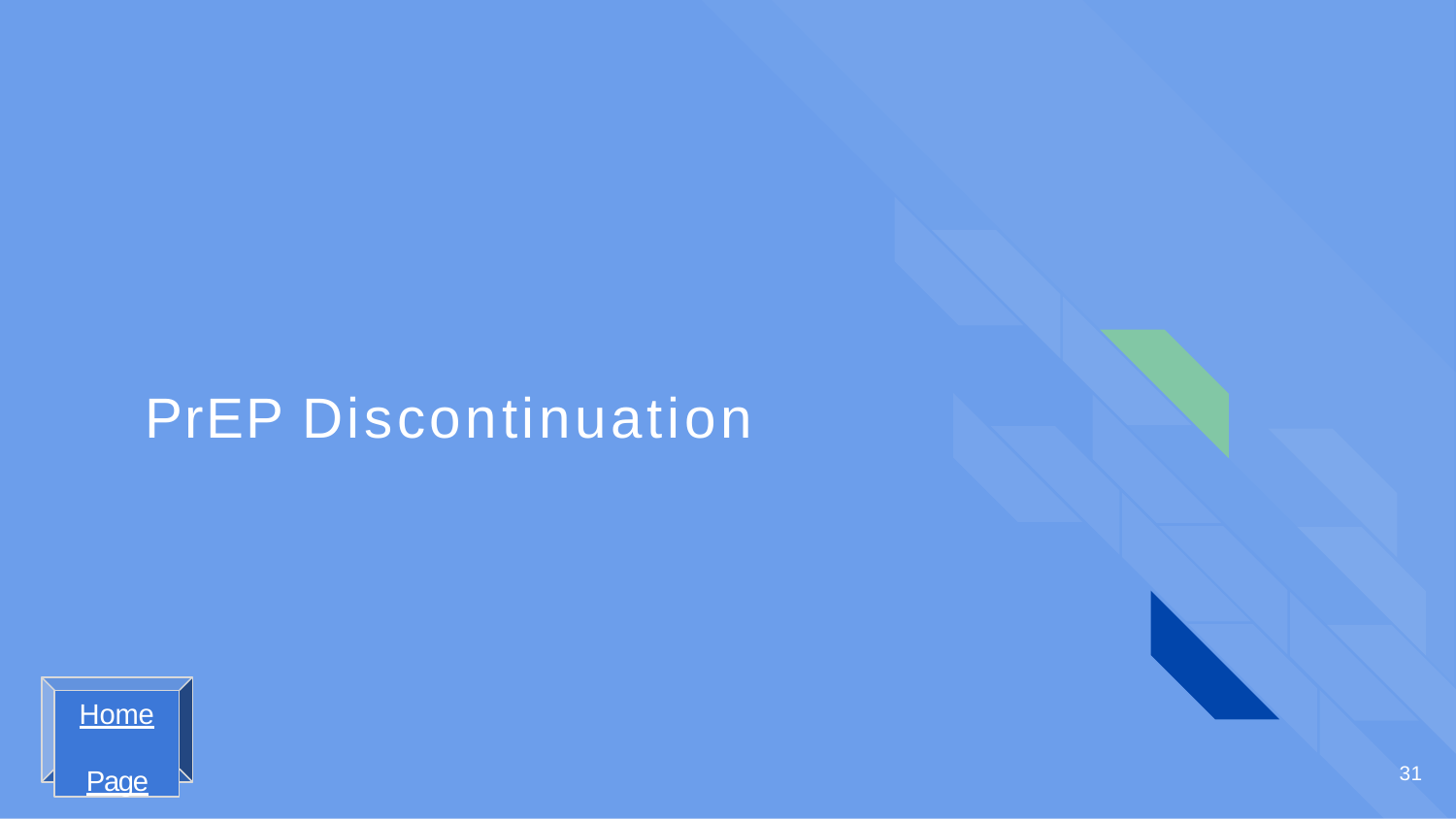

PrEP Discontinuation
Home Page
31

## Slide 32
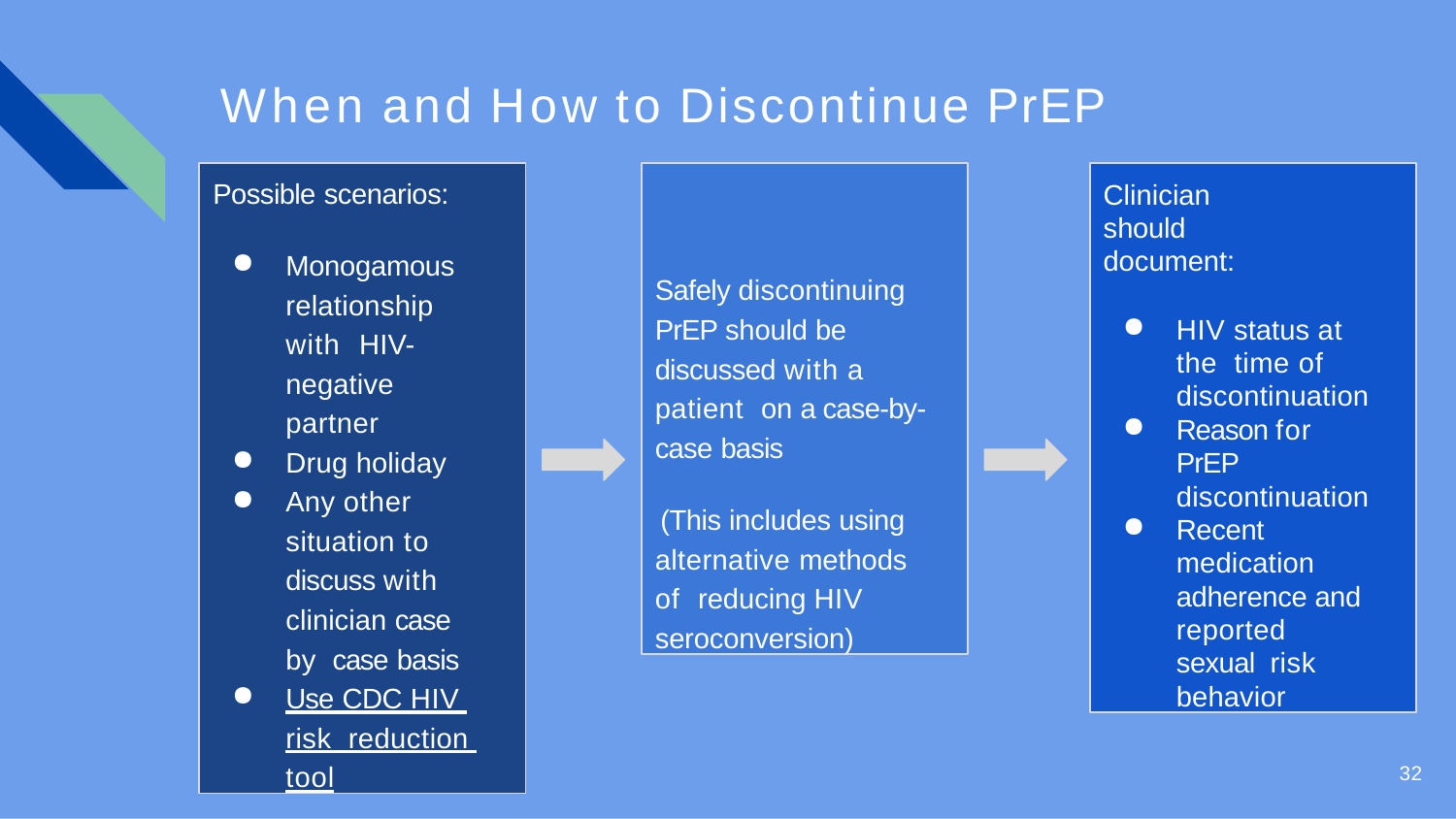

# When and How to Discontinue PrEP
Possible scenarios:
Monogamous relationship with HIV-negative partner
Drug holiday
Any other situation to discuss with clinician case by case basis
Use CDC HIV risk reduction tool
Safely discontinuing PrEP should be discussed with a patient on a case-by-case basis
(This includes using alternative methods of reducing HIV seroconversion)
Clinician should document:
HIV status at the time of discontinuation
Reason for PrEP discontinuation
Recent medication adherence and reported sexual risk behavior
32

## Slide 33
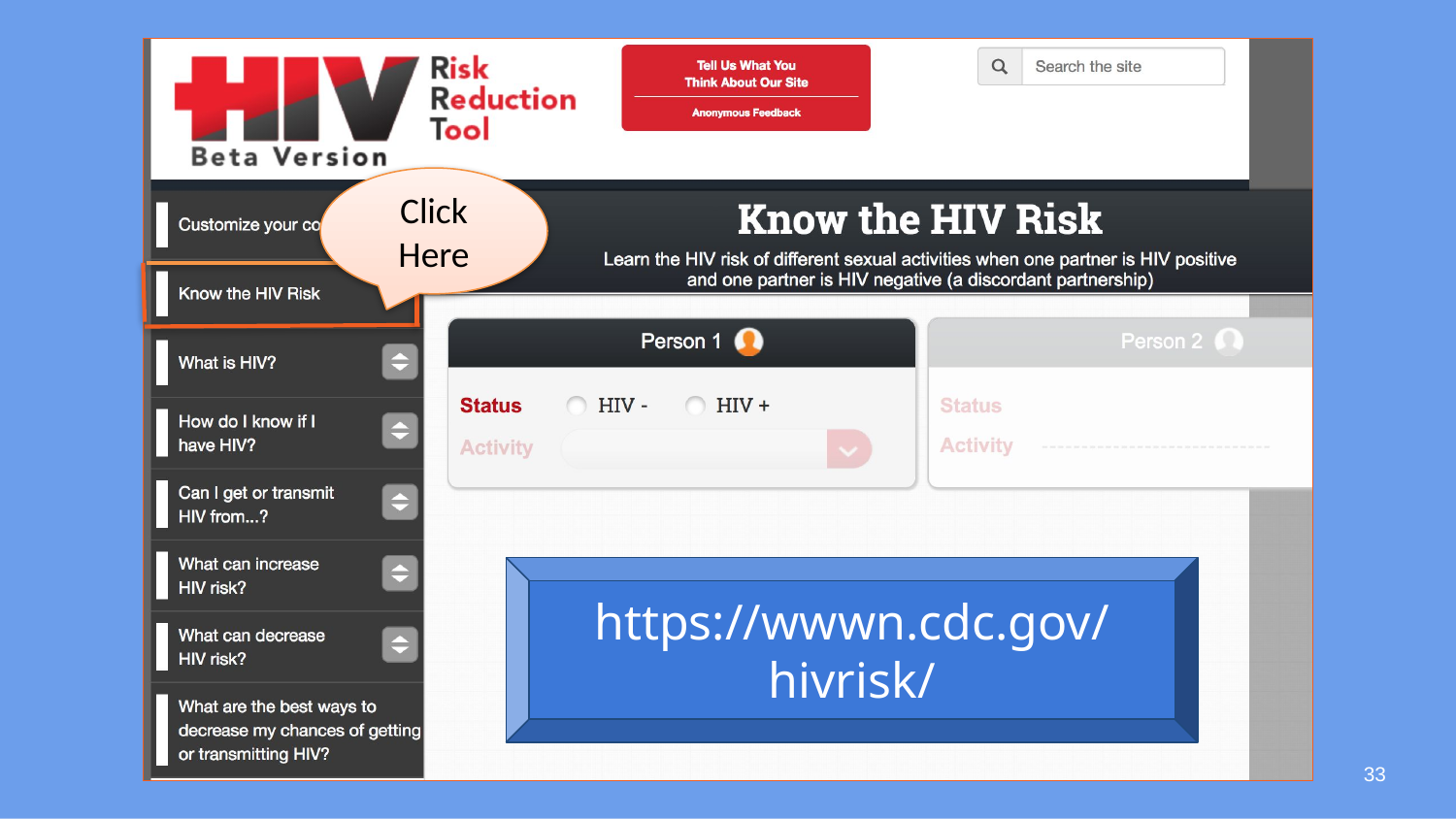

Click Here
https://wwwn.cdc.gov/hivrisk/
33

## Slide 34
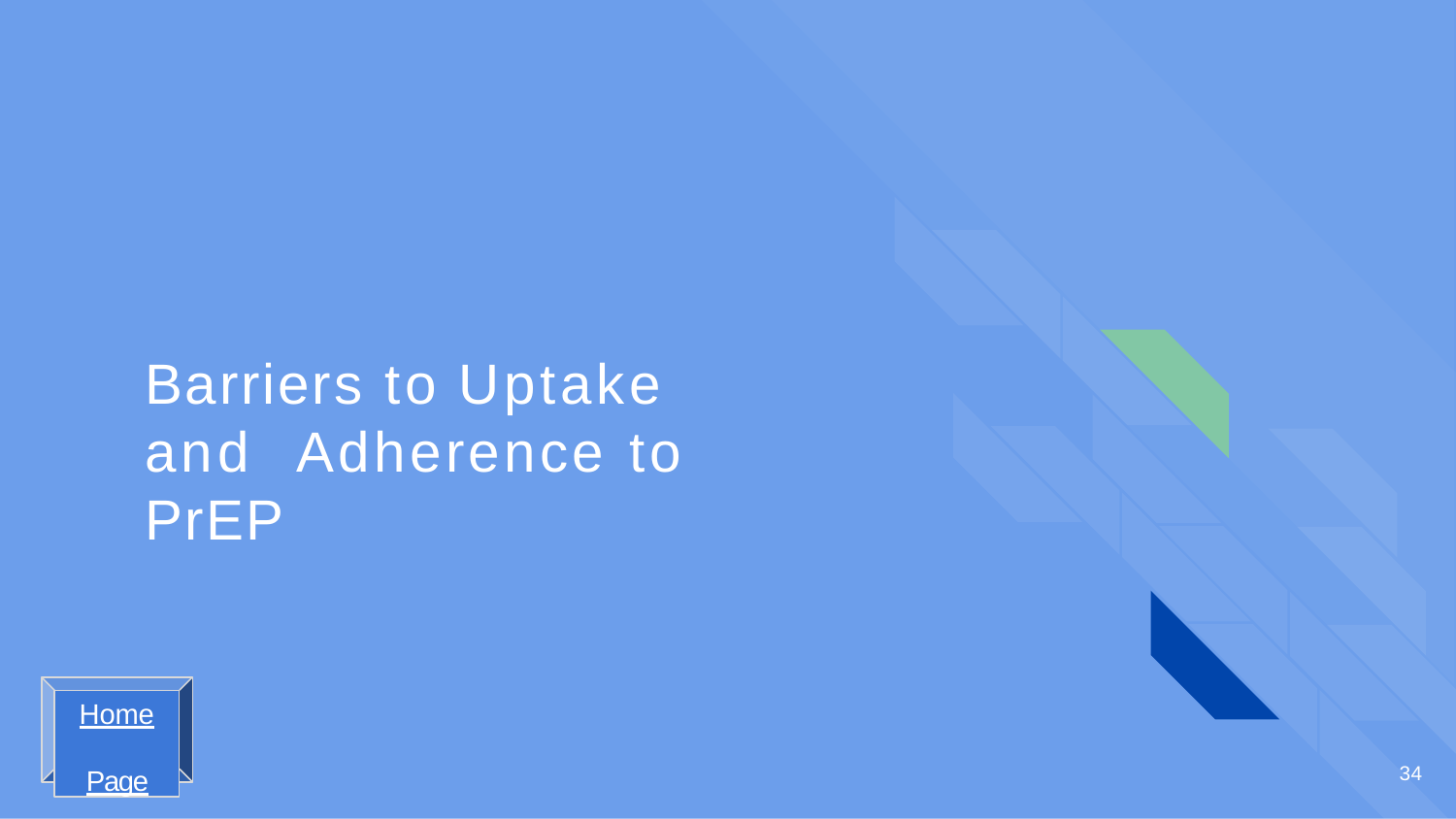

# Barriers to Uptake and Adherence to PrEP
Home Page
34

## Slide 35
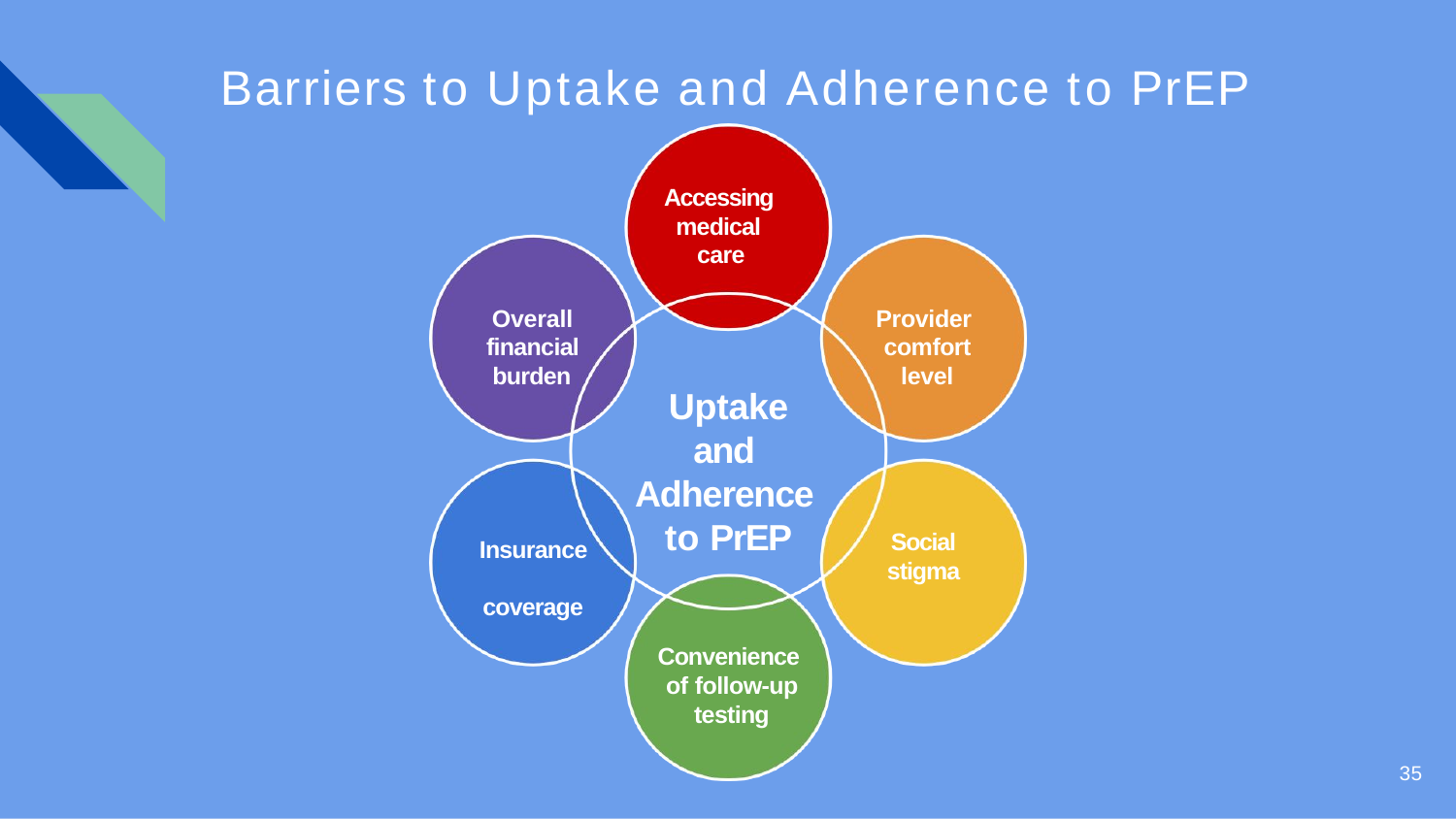

# Barriers to Uptake and Adherence to PrEP
Accessing medical care
Overall financial burden
Provider comfort level
Uptake and Adherence to PrEP
Social stigma
Insurance coverage
Convenience of follow-up testing
35

## Slide 36
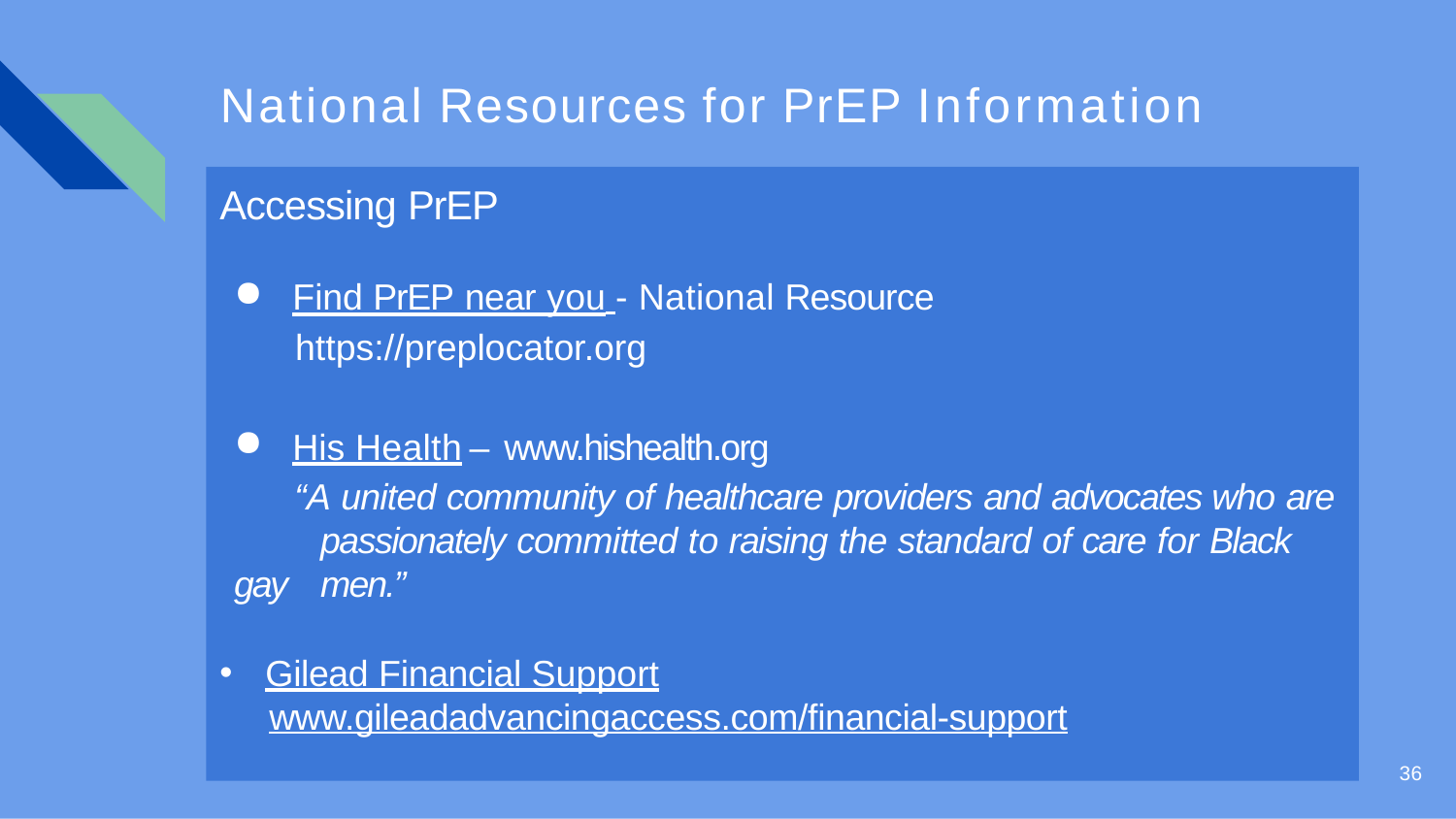

# National Resources for PrEP Information
Accessing PrEP
Find PrEP near you - National Resource
 https://preplocator.org
His Health – www.hishealth.org
 “A united community of healthcare providers and advocates who are 	passionately committed to raising the standard of care for Black gay 	men.”
Gilead Financial Support
 www.gileadadvancingaccess.com/financial-support
36

## Slide 37
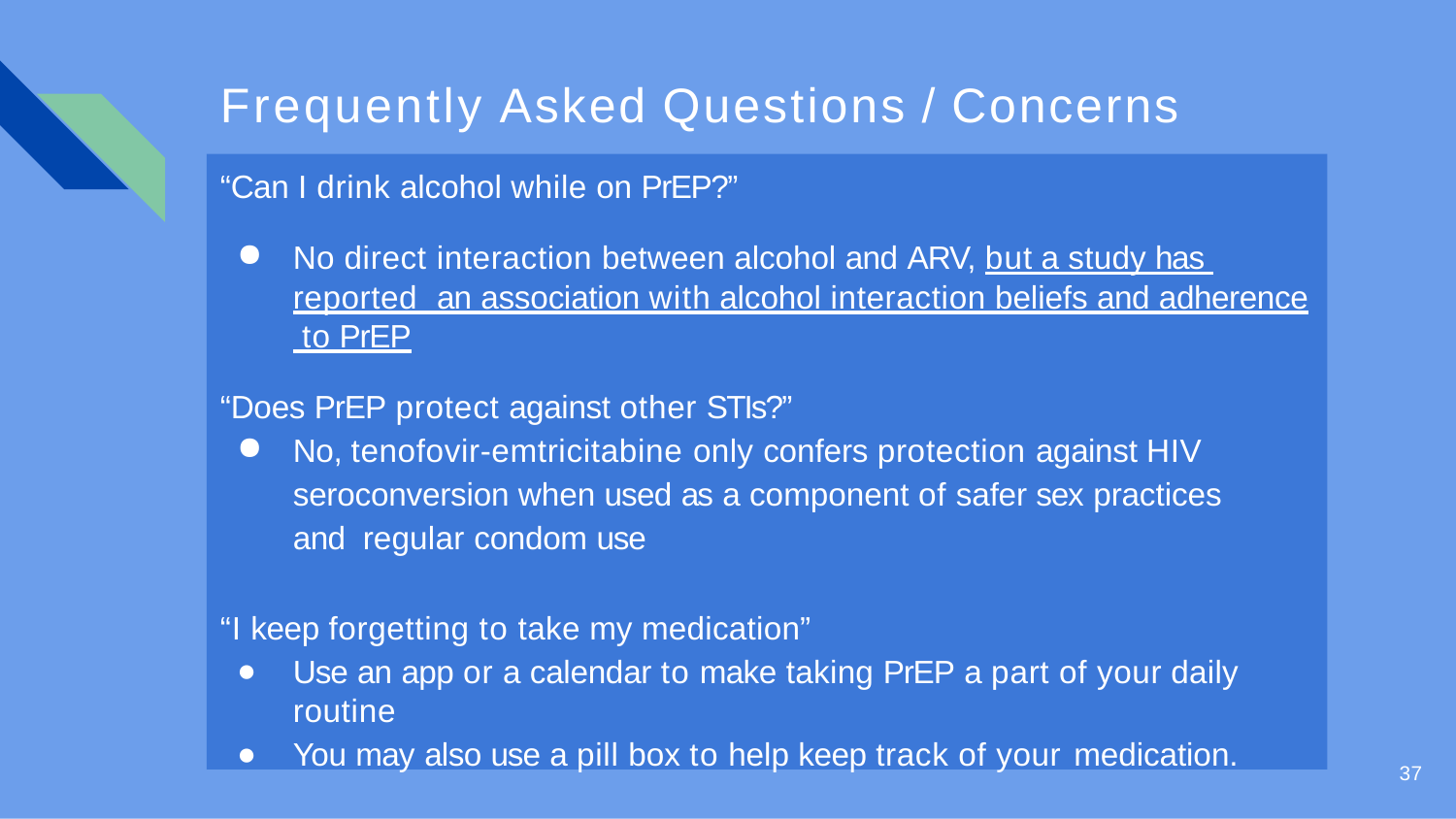

# Frequently Asked Questions / Concerns
“Can I drink alcohol while on PrEP?”
No direct interaction between alcohol and ARV, but a study has reported an association with alcohol interaction beliefs and adherence to PrEP
“Does PrEP protect against other STIs?”
No, tenofovir-emtricitabine only confers protection against HIV seroconversion when used as a component of safer sex practices and regular condom use
“I keep forgetting to take my medication”
Use an app or a calendar to make taking PrEP a part of your daily routine
You may also use a pill box to help keep track of your medication.
37

## Slide 38
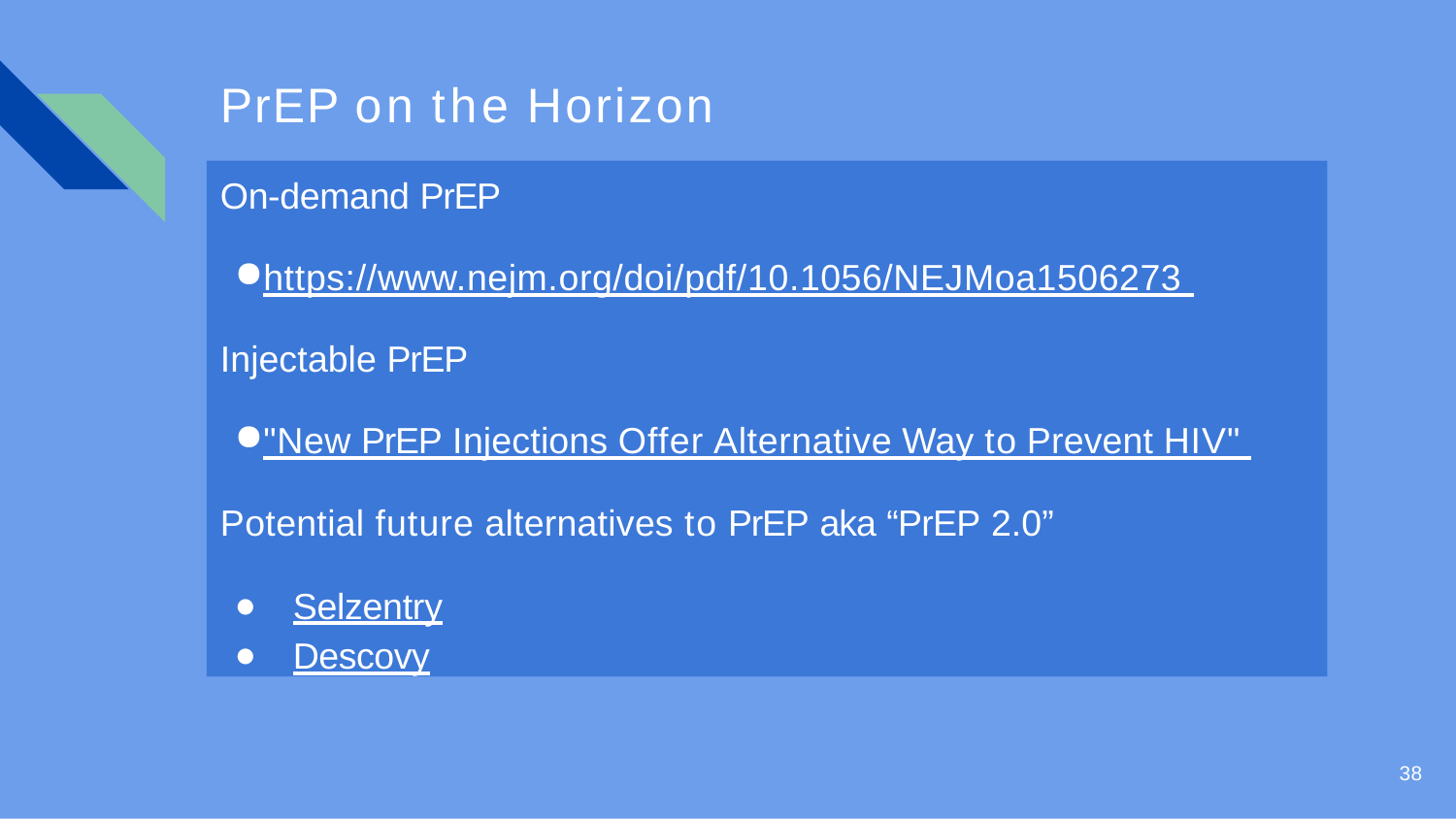

# PrEP on the Horizon
On-demand PrEP
https://www.nejm.org/doi/pdf/10.1056/NEJMoa1506273 Injectable PrEP
"New PrEP Injections Offer Alternative Way to Prevent HIV" Potential future alternatives to PrEP aka “PrEP 2.0”
Selzentry
Descovy
38

## Slide 39
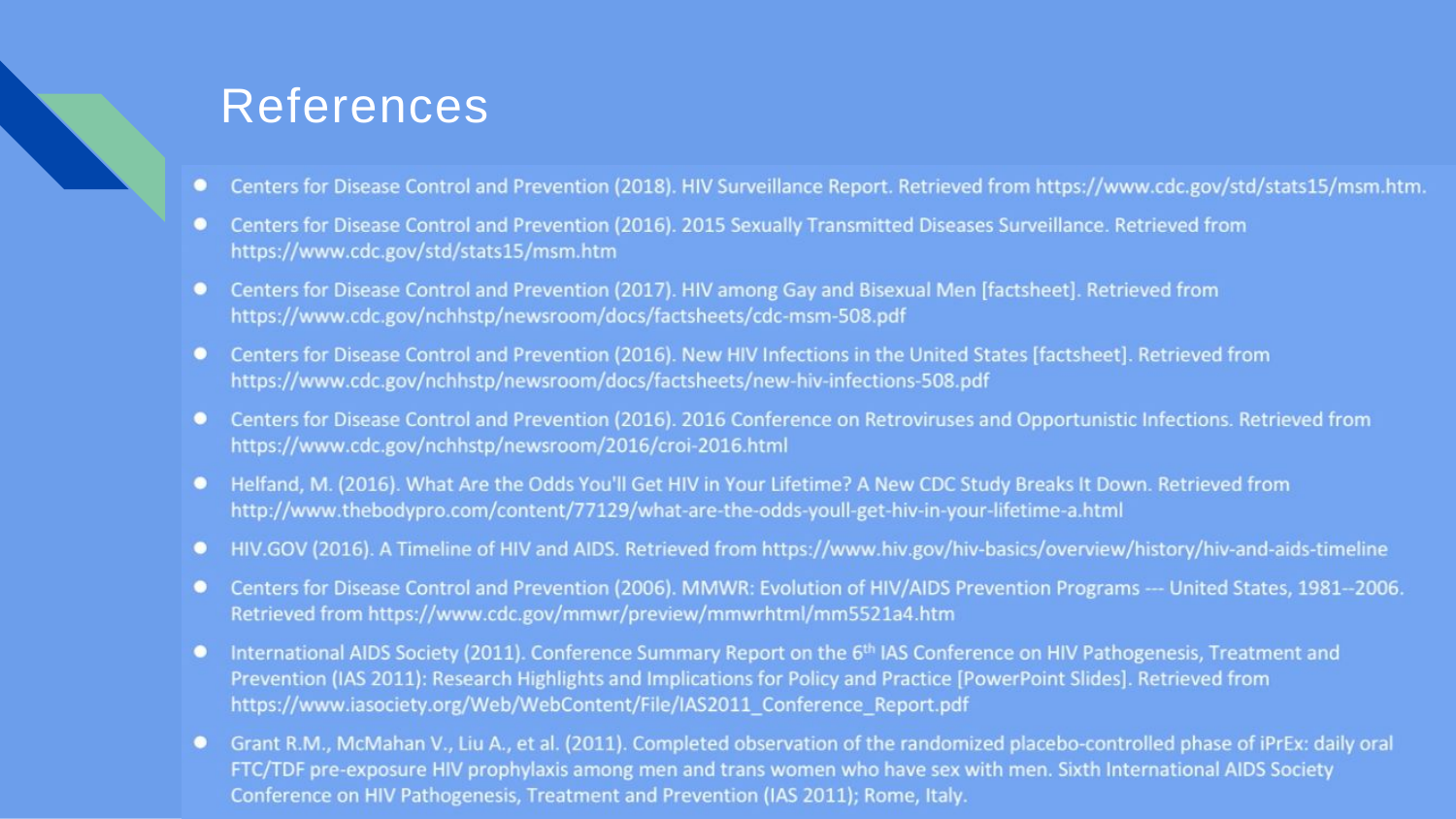

# References
39

## Slide 40
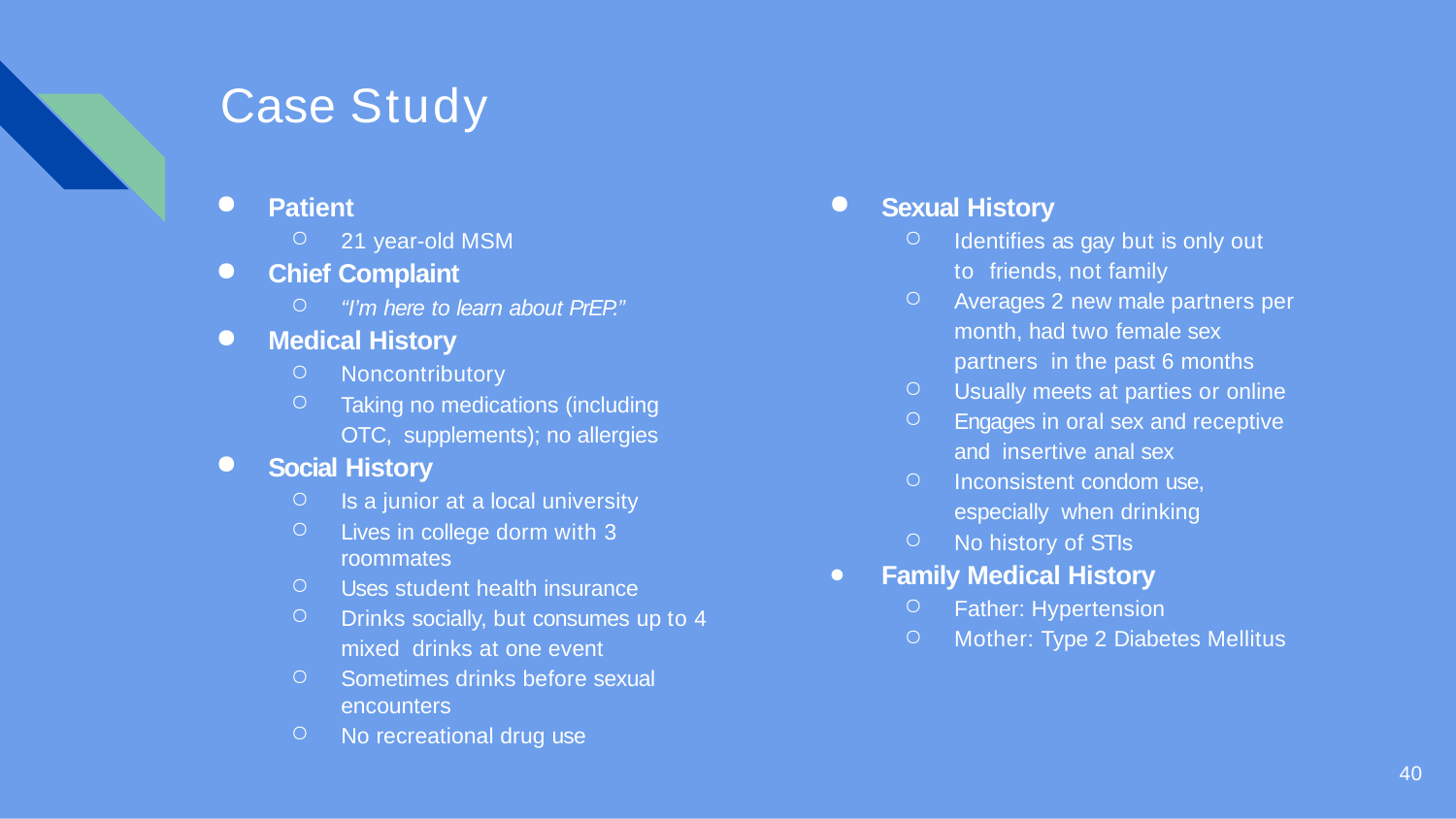

# Case Study
Patient
21 year-old MSM
Chief Complaint
“I’m here to learn about PrEP.”
Medical History
Noncontributory
Taking no medications (including OTC, supplements); no allergies
Social History
Is a junior at a local university
Lives in college dorm with 3 roommates
Uses student health insurance
Drinks socially, but consumes up to 4 mixed drinks at one event
Sometimes drinks before sexual encounters
No recreational drug use
Sexual History
Identifies as gay but is only out to friends, not family
Averages 2 new male partners per month, had two female sex partners in the past 6 months
Usually meets at parties or online
Engages in oral sex and receptive and insertive anal sex
Inconsistent condom use, especially when drinking
No history of STIs
Family Medical History
Father: Hypertension
Mother: Type 2 Diabetes Mellitus
40
