# Supplementary material for: PrEP (Pre-Exposure Prophylaxis) Education for Clinicians: Caring for an MSM Patient
Source: MedEdPORTAL. 2020 May 29;16:10908. doi: 10.15766/mep_2374-8265.10908 (PMC7336890; doi:10.15766/mep_2374-8265.10908)
Supplement: Supplementary file 1 — Presentation.pptxPresentation with Audio.pptxDiscussion Guide.docxPatient-Physician Video.mp4Preworkshop Evaluation.docxPostworkshop Evaluation.docx [file mep_2374-8265.10908-s001.zip › C. Discussion Guide.docx]

PrEP Discussion Guide (English)

**Slide 1**

Title Slide

**Slide 2**

Hello everyone, thank you for joining us for today’s presentation for pre-exposure prophylaxis education for clinicians. My name is _________________ (credentials). Today we will be taking you through an overview of pre-exposure prophylaxis or PrEP, with a specific emphasis in the MSM population.

**Slide 3**

This slide provides a quick overview of today’s topics. First we will go through a quick introduction of HIV and PrEP, including a history of HIV prevention. Next, we will examine the determination for eligibility, and initial testing in patients who may benefit from PrEP. We will then move on to discuss appropriate steps in maintenance, on monitoring of health of patients on PrEP. Then we will discuss potential scenarios in which a patient may discontinue PrEP, plus the appropriate steps that you, as the clinician, must take in order to safely discontinue medication. Lastly we will explore potential barriers to the use of PrEP, including barriers to uptake and adherence, as well as some resources that may help your patients access and pay for PrEP more easily.

**Slide 4**

**Learning Objectives**

Here are the learning objectives for today’s session. Objectives include, to describe the history of HIV prevention, learn the indications for PrEP prescription, describe appropriate testing and medical care for patients starting Prep, describe common adverse reactions of PrEP medication, explain appropriate follow-up and testing for PrEP maintenance, describe indications for PrEP discontinuation, and describe the barriers for PrEP adherence.

**Slide 5**

**Definitions**

It is important to review definitions prior to continuing with the presentation. (review definitions)

**Slide 6**

**Introduction to HIV and PrEP**

**Slide 7**

Here we review HIV transmission rates from 2015, by transmission category according to the CDC HIV Surveillance Report. It is important to highlight that male-to-male sexual contact is responsible for the highest number of new HIV infections compared to other transmission types. Despite public health initiatives targeting the MSM communities, this group remains a high incidence cohort for HIV infections. Therefore, continued education and efforts are needed to reduce HIV transmission in this high risk group.

**Slide 8**

This slide shows the disparities that continue to exist within the MSM community. Despite effort, we have failed to broach the gap within racial and ethnic groups, most notably within the black and Latino community. Particularly poignant is the fact that the lifetime risk of HIV diagnosis for black MSM is 1 in 2 and for Latino MSM is 1 in 4.

**Slide 9**

**Timeline**

Depicted here, is a brief timeline of HIV, as well as the result in prevention strategies. These strategies were implemented almost immediately, starting in 1981, when the first cases of immune deficiency were described. During the mid-1980’s, the regular use of condoms was promoted as a means of reducing HIV transmission. This was followed by needle exchange programs in the 1990’s as well as concerted grass-roots efforts from community leaders in minority communities to address HIV prevention. Since the 1980’s, efforts to develop vaccines against HIV have failed to produce an effective immunization strategy. It wasn’t until 2012, that the FDA approved an oral medication for HIV pre-exposure prophylaxis, in large part supported by a multinational clinical trial known as IPREX.

**Slide 10**

**IPREX trial**

The IPREX trial, also known as Iniciativa prophylaxis pre-exposicion, was a randomized, double-blind, placebo controlled phase 3 clinical trial, of once daily oral truvada, also known as emtricitabine and tenofivir, for HIV prevention. It occurred between 2007 and 2011 in Brazil, Ecuador, Peru, South Africa, Thailand, and the EEUU.

Participants included 2499 HIV-seronegative men or transgender women who have sex with men. 1251 were assigned to the truvada group. 1248 were assigned to the placebo group. Results included 131 participants who became HIV+, 48 in the truvada group and 83 in the placebo group. Overall, truvada reduced the rate of new infections by 42%* and by 92% in those with detectable drug levels. Just for clarification, after a modified intention to treat analysis, truvada reduced the rate of new infections by 42 percent, in the overall truvada group. However, in those with detectable drug levels in their blood, the rate of new infections was reduced by 92% (to be discussed in a later slide).

**Slide 11**

**IPrEx open label extension study**

The design included participants from other PrEP trials to continue studying the safety, adherence and efficacy of taking once daily oral truvada for HIV prevention. The study occurred from 2010 onward, after which results were presented in July 2014. The 11 sites still included Brazil, Ecuador, Peru, South Africa, Thailand, and USA. Participants in the open label extension study included 1603 HIV-seronegative men or transgender women who have sex with men. 1255 participants continued on PrEP. 41 participants became infected. 28 in the truvada group and 13 in control group. Overall, truvada reduced the rate of new infections by 49% after adjusting for baseline sexual risk.

**Slide 12**

**Adherence table, truvada**

When looking more closely at risk reduction based on adherence to truvada, which was confirmed by detectable drug levels in patient blood samples, we can see that poor to little compliance with medication explains low risk reduction of transmission for certain participants. However, we see that in participants with moderate to excellent compliance, optimal HIV transmission risk reduction was achieved. Although it is not recommended that a patient miss doses, even missing a few doses can confer some protective ability. If your patient misses one dose periodically, you can reassure them they are still receiving a protective benefit from their PrEP regimen.

**Slide 13**

**Summary of Guidance**

Depicted here, is a summary of guidance for PrEP use for the MSM and HSW/M population (Heterosexual women and men). Patients who satisfy any of the following criteria may be eligible for PrEP. It should be noted that a high number of sex partners is loosely defined but has been described in some studies as around 5 or more in the past 12 months. However, each individual’s behaviors and practices should be addressed on a case by case basis. Another population who may benefit is injectable drug users, however, for the purposes of this presentation we will be focusing on MSMs who do not inject drugs.

**Slide 14**

**Determination of Eligibility and Initial Testing**

**Slide 15**

**Starting PrEP**

Shown here, are recommended indications for MSMs. First being, an adult or adolescent man weighing at least 35kg, without any acute or established HIV infection, who has had any male partners in the past 6 months, who is not in a monogamous relationship with a recently tested HIV negative man AND at least one of the following:

Engages in anal sex without condoms, either receptive or insertive, in the past 6 months

Has a history of bacterial STI, including syphilis, gonorrhea, or chlamydia, diagnosed or reported in the past 6 months

Or any patient in a serodiscordant relationship

A patient who always uses condoms or only engages in oral sex may also request to start on PrEP, given that there are no contraindications to initiating therapy. It is ok to prescribe to these patients giving proper counseling on the short and long-term side effects as well as the importance of adherence.

**Slide 16**

**Starting PrEP: Clinical Questions for the MSM Patient**

When starting PrEP, it is important to ask the following screening questions to see if your patient is a candidate:

In the past 6 months, Have you been in an exclusively monogamous relationship with an HIV negative male partner? If no, the patient may be a candidate

With how many male partners have you engaged in anal sex, either receptive or insertive? If the answer is 1 or more times, the patient may be a candidate

How many times did you have receptive or insertive anal sex where one of you was not wearing a condom? If the answer is 1 or more times, the patient may be a candidate

Have you been diagnosed with an STI? If the patient answers yes, they may be a PrEP candidate.

Have you used alcohol or any illicit substances, such as methamphetamines, before a sexual encounter? If the patient answers yes, they may also be a PrEP candidate.

**Slide 17**

**Who is not eligible for PrEP**

Who is not eligible for PrEP, an HIV positive patient, or one who is acutely infected with HIV. A patient with renal disease which includes an estimated creatinine clearance rate of less than 60ml/min, a patient, who for any reason, is on high doses of NSAIDs, or a patient who is on any nephrotoxic agents, including: acyclovir, valacyclovir, cidofovir, ganciclovir, valganciclovir, and aminoglycosides. Or a patient with a history of pathologic or fragility fractures OR who has significant risk factors for osteoporosis. Of note, Hepatitis B infection is not a contraindication to PrEP use, but PrEP adherence is critical for these patients. It is important that these patients are also co-managed with an infectious disease or hepatic disease specialist.

**Slide 18**

**PrEP is not PEP**

PEP is post exposure prophylaxis; it is a treatment to reduce risk of HIV transmission. What is PEP? Essentially, it is PrEP but it includes Isentress or raltegravir. PEP is used if an individual is HIV-negative or does not know their HIV status and in the last 72 hours:

May have been exposed to HIV during sex

Shared needles and paraphernalia to prepare injectable drugs

Or were sexually assaulted

If a patient is on PrEP during known exposure, you may add raltegravir to their tenofovir and emtricitabine regimen.

**Slide 19**

**How long does it take to work?**

According to the CDC, maximum protection is achieved in rectal tissue in 5 to 7 days and in vaginal tissue in about 20 days. According to the world health organization, maximum protection is achieved in, 4 doses for anal sex, and 7 doses for vaginal sex. Penile tissue has not been studied. For the purposes of patient education, we will follow the CDC guidelines.

**Slide 20**

**Daily Use Vs Moderate Compliance – IprEX Study**

Here is a graphic representation of PrEP efficacy by adherence to Truvada and by detectable drug blood level. Patience with at least moderate compliance showed great benefit against HIV seroconversion. Again, this highlights that if a patient misses 1-2 doses periodically, you can assure them they still receive protective benefit from their PrEP regimen.

**Slide 21**

**Initial Testing and vaccinations for PrEP**

HIV testing: HIV testing includes a documented negative anti-body test within the week before initiating, ideal an anti-body antigen test. Blood, serum or rapid point of care fingerstick is acceptable. Oral fluid tests are not sensitive enough. Viral RNA testing to test for acute HIV infection. If the patient tests preliminarily positive on the HIV antibody test, this must be confirmed by a local laboratory standardized practice, which may include viral load and CD4 count.

**Slide 22**

**Continued…**

STI testing, most notably for gonorrhea and chlamydia, will include a nucleic acid amplification test in 3 sites, pharyngeal, rectal and urine specimens.

Liver and kidney testing includes Hepatitis B virus serology, and as noted earlier, hepatitis B vaccination is not a contraindication for PrEP use. Hepatitis B diagnosis does not exclude from PrEP use either. Adherence, again, is more important for these patients. As for kidney function, an estimated creatinine clearance of greater than 60ml/min is ideal.

**Slide 23**

**Vaccinations**

HPV: vaccinate if your patient is under 46 years old

For hepatitis B, vaccinate if they are susceptible

For hepatitis A, vaccinate if your patient had never been vaccinated before

Other vaccines include, influenza, meningococcal depending on geography and pneumococcus if indicated

**Slide 24**

**How is PrEP prescribed**

How is PrEP prescribed. Truvada is prescribed as one oral pill daily with a 90-day supply during onboarding. Patients must return at 3 months for their follow-up visit. The following information is given from Gilead: The dosage of TRUVADA in HIV-1 uninfected adults and adolescents weighing at least 35kg is one tablet, containing 200mg of Tenofivir and 300mg of emtricitibine, taken once daily orally with or without food.

**Slide 25**

**Maintenance and Monitoring**

**Slide 26**

**Side Effects and Adverse Reactions**

Shown here are selected adverse reactions from the IPREX study as well as the statistical significance of each. Headaches, nausea and diarrhea usually resolve within the first 2 weeks and you should encourage your patient to continue with the medicine. Again if creatinine is above 60, they can still continue on with the drug. Nausea and unintentional weight loss were the only 2 adverse events that were found to be statistically significant.

**Slide 27**

**Follow up testing for PrEP**

Follow up testing at every 3 months should include an HIV test, medication adherence counseling, behavioral risk reduction support, side effect assessment, STI symptom assessment and test for bacterial STIs. At 3 months and every 6 months thereafter, you should also test for renal function. More frequent monitoring is recommended if comorbidities such as hypertension or diabetes mellitus are present. As a reminder, a rise in creatinine is not a reason to withhold if the estimated creatinine clearance rate remains above 60ml/min

**Slide 28**

**Follow up testing**

Every 12 months, you should reassess whether or not your patient should continue on PrEP. Optional assessments include bone health, although it is unclear if HIV uninfected are as at risk as HIV infected persons treated with the combination ART. DEXA is not recommended for those patients on PrEP, unless there is a history of pathologic or fragility fractures OR the patient also has other risk factors for osteoporosis.

**Slide 29**

**Prep Discontinuation**

**Slide 30**

**When and How to discontinue PrEP?**

Possible scenarios that your patient may offer you include, starting a monogamous relationship with an HIV-negative partner, a drug holiday during which the patient no longer wishes to be on medication, or any other situation to discuss with clinician on a case by case basis. In any case, it is important that you use the CDC’s HIV risk reduction tool to help your patient understand their risk. Safely discontinuing PrEP should be discussed with the patient on a case by case basis. This also includes using alternative methods of reducing HIV seroconversion, including sexual risk behavior modification and barrier protection. At the time, clinicians should document the HIV status, the reason for PrEP discontinuation, and also the patient’s recent medication adherence and reported sexual risk behavior.

**Slide 31**

**HIV tool**

Show here is a screen shot of the HIV’s risk reduction tool. You can customize your patient’s risk including their HIV status as well as their sexual activity. If you’d like more information, please refer to the CDCs HIV risk reduction tool.

**Slide 32**

**Barriers to uptake and adherence to PrEP**

Uptake and adherence to PrEP is often confounded by many barriers. The first being accessing medical care.

Access, especially in underserved communities, poor access to insurance and to culturally competent clinicians, is often the first barrier to care.

Provider comfort level. It is important that you as a provider, be well versed not only in the indications, mechanisms and side effects of PrEP, but also in how to talk to your patient and to provide them a safe and non-judgmental atmosphere.

Social Stigma. The social stigma against LGBTQ individuals still exists. Embarrassment sometimes contributes to patients not seeking care.

Convenience of follow up testing. Multiple rounds of follow up testing are needed for PrEP maintenance. This barrier is important for patients who work or who need child care services.

Insurance coverage. Varying levels of coverage exist, so it is important that you talk to your care coordination and social work teams to figure out what is best for your patient and how to cover gaps that may be left if not all of cost is covered by insurance.

Overall financial burden. This can be comprised of other financial barriers, which include, childcare services, missed time from work, transportation issues, parking, etc.

**Slide 33**

**National Resources for PrEP Information**

Provided are national resources for PrEP information, may need to be modified based on local geography.

**Slide 34**

**Frequently asked questions**

Frequently asked questions or concerns.

Can I drink alcohol while on PrEP?

While there is no direct interaction between and antiretroviral drugs, there has been a study that reported an association with alcohol interaction beliefs and adherence to PrEP.

Does PrEP protect against other STIs?

Truvada only confers protection against HIV seroconversion when used as a component of safer sex practices and regular condom use.

“I keep forgetting to take my medication”

It may be useful to use an app or a calendar to make taking PrEP a part of a patient’s daily routine. It may also be advisable to use a pill box to help keep track of medication.

**Slide 35**

**PrEP on the horizon**

PrEP on the horizon, links

**Slide 36**

**References**

Included here, are references.

**Slide 37**

**Case Study**

We will now show you a scripted clinical encounter for best practices in PrEP prescribing. The patient here is a 21-year-old male; whose chief complaint is “I am here to learn more about PrEP.” His Medical history is non-contributory. He takes no medications, including no over-the-counter medications and no supplements. He has no allergies.

The social history includes, education as a junior at a local university, who lives in a college dorm with 3 roommates. He uses student health insurance. He drinks socially, but consumes up to 4 mixed drinks at one event. Sometimes drinks before sexual encounters. He does not engage in recreational drug use.

The sexual history includes, identification as gay, but is only out to friends, not family. Averages 2 new male partners per month but has had 2 female sex partners in the past 6 months. Usually meets his partners at parties or online. He engages in oral sex, as well as insertive and receptive anal sex. His condom use is inconsistent, especially when drinking. He has no history of STIs.

His family medical history includes, a father with hypertension and a mother with type II diabetes mellitus.

During this portion of the presentation, the facilitator can select from the following options:

1. Real time demonstration: The facilitator acts out the described clinical encounter with a volunteer. The facilitator can play the role of the provider and demonstrate appropriate clinical communication skills used in assessing risk factors for HIV acquisition and PrEP candidacy. A co-facilitator or participant volunteer can play the role of the patient. Upon completion of the demonstration, the facilitator should ask for audience feedback on recommendations for communication strategies used in assessing risk factors for HIV acquisition and PrEP candidacy. It is important to share with participants that communication skills can vary from clinician to clinician, and it is important to listen to and receive feedback from their professional colleagues and patients.
2. Play videotaped clinical encounter: The facilitator plays the videotaped clinical encounter for the audience (see Appendix D). After watching the video, the facilitator should ask for audience feedback on recommendations for communication strategies used in assessing risk factors for HIV acquisition and PrEP candidacy. They should comment on what they liked or did not like about the communication skills used, and reflect on how the skills demonstrated influence their own clinical practices. It is also important to share with participants that communication skills can vary from clinician to clinician, and it is important to listen to and receive feedback from their professional colleagues and patients. *For example, during the videotape, the provider asks the patient if he is in any romantic relationships. It is important to point out to the learners that sexual encounters may or may not be secondary to romantic relationships and some patients may not disclose sexual encounters if they are limited to romantic relationships.*
