# Supplementary material for: PrEP (Pre-Exposure Prophylaxis) Education for Clinicians: Caring for an MSM Patient
Source: MedEdPORTAL. 2020 May 29;16:10908. doi: 10.15766/mep_2374-8265.10908 (PMC7336890; doi:10.15766/mep_2374-8265.10908)
Supplement: Supplementary file 1 — Presentation.pptxPresentation with Audio.pptxDiscussion Guide.docxPatient-Physician Video.mp4Preworkshop Evaluation.docxPostworkshop Evaluation.docx [file mep_2374-8265.10908-s001.zip › F. Postworkshop Evaluation.docx]

**PrEP Education for Clinicians Post Presentation Survey**

- Required
  1. **After watching the education video presentation on PrEP, please answer the following questions. Please write your unique ID: ***
  2. **I feel comfortable discussing sexual behaviors with MSM**

*Mark only one oval.*

1 2 3 4 5

Strongly Disagree Strongly Agree

- 1. **I feel comfortable identifying at risk MSM who can benefit from PrEP**

*Mark only one oval.*

1 2 3 4 5

Strongly Disagree Strongly Agree

- 1. **I feel comfortable knowing when to prescribe PrEP to MSM**

*Mark only one oval.*

1 2 3 4 5

Strongly Disagree Strongly Agree

- 1. **I am knowledgeable about the indications for when to prescribe PrEP to MSM**

*Mark only one oval.*

1 2 3 4 5

Strongly Disagree Strongly Agree

- 1. **I am knowledgeable about the side effects of PrEP**

*Mark only one oval.*

1 2 3 4 5

Strongly Disagree Strongly Agree

- 1. **I am knowledgeable about the recommended screening tests for MSM interested in starting PrEP**

*Mark only one oval.*

1 2 3 4 5

Strongly Disagree Strongly Agree

- 1. **I am knowledgeable about indications for PrEP discontinuation for MSM**

*Mark only one oval.*

1 2 3 4 5

Strongly Disagree Strongly Agree

- 1. **Which of the following test is unnecessary prior to prescribing PrEP?**

*Mark only one oval.*

Basic Metabolic panel CBC

Hepatitis B serologies

HIV antibody

- 1. **The lifetime risk of HIV diagnosis among black MSM is**

*Mark only one oval.*

1 in 2

1 in 4

1 in 5

1 in 10

- 1. **Who is NOT eligible for PrEP?**

*Mark only one oval.*

Patient with creatinine clearance of 65 ml/min Active Hepatitis B infection

Acute Hepatitis C infection

Patient history of pathologic fractures Active herpes infection on acyclovir

- 1. **How is PrEP prescribed?**

*Mark only one oval.*

1 tablet daily

1 tablet twice a day

Intramuscular injection once a week Intramuscular injection once a month

- 1. **After initiating PrEP, when should patients see their doctor for repeat blood work?**

*Mark only one oval.*

- - 1. month
    2. months
    3. months

6 months

- 1. **What is the most common side effects of PrEP?**

*Mark only one oval.*

Headaches Vomiting Weight loss

Elevated creatinine

- 1. **When discontinuing PrEP the clinician should document all of the following EXCEPT**

*Mark only one oval.*

HIV status at the time of discontinuation Reason for PrEP discontinuation Recent travel outside the United States Reported sexual risk behavior

- 1.
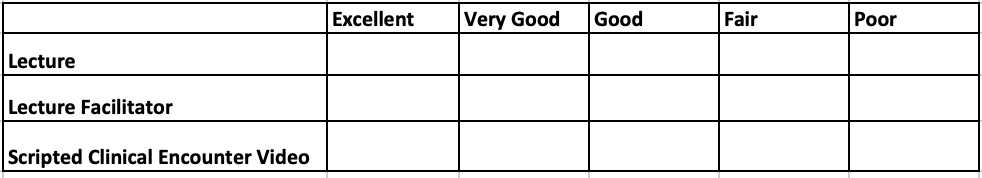
**Please rate the following workshop elements:**
  2. **Please comment on the strengths of the workshop**
  3. **Please include your suggestions for the workshop**
