# Supplementary material for: PrEP (Pre-Exposure Prophylaxis) Education for Clinicians: Caring for an MSM Patient
Source: MedEdPORTAL. 2020 May 29;16:10908. doi: 10.15766/mep_2374-8265.10908 (PMC7336890; doi:10.15766/mep_2374-8265.10908)
Supplement: Supplementary file 1 — Presentation.pptxPresentation with Audio.pptxDiscussion Guide.docxPatient-Physician Video.mp4Preworkshop Evaluation.docxPostworkshop Evaluation.docx [file mep_2374-8265.10908-s001.zip › _Educational_Summary_Report_10908.pdf]

MedEdPORTAL® | The AAMC Journal of Teaching and Learning Resources

Original Publication

Open Access

PrEP (Pre-Exposure Prophylaxis) Education for Clinicians: Caring for an MSM Patient

James Perucho, MLS, Luis Alzate-Duque, MD, Amir Bhuiyan, John Paul Sánchez, MD, MPH, Nelson Felix Sánchez, MD*

*Corresponding Author: nfs1978@yahoo.com

Abstract

Introduction: Gaps exist in educational materials addressing LGBTQ patient care and LGBTQ health. One such area is prescribing HIV pre-exposure prophylaxis (PrEP) for men who have sex with men (MSM). PrEP awareness, familiarity, and comfort in prescribing are very important in the rollout and success of PrEP as a preventative measure. Our needs assessments showed a lack of familiarity and comfort among clinicians/medical students in prescribing PrEP. Furthermore, studies have shown that since its launch as an effective prevention method of HIV transmission, PrEP has not been widely prescribed to at-risk populations. Educating clinicians about PrEP may increase its use among high-risk MSM populations and reduce the incidence of HIV infections. **Methods:** For medical students, we developed a didactic presentation and video recording discussing (1) a brief history of HIV prevention, (2) indications for PrEP prescription, (3) medical testing for PrEP onboarding, (4) common PrEP side effects, and (5) appropriate follow-up and testing for PrEP maintenance and discontinuation. We also developed a videotaped clinical encounter demonstrating communication skills used in PrEP counseling. Pre- and postworkshop surveys assessed participants' PrEP attitudes and knowledge. **Results:** All 43 survey respondents were second- through fourth-year medical students. Pre- and postpresentation evaluation of questions assessing comfort demonstrated a statistically significant improvement in level of comfort with understanding when to prescribe PrEP and in level of knowledge in prescribing PrEP. **Discussion:** Workshop participants acknowledged their training gaps in PrEP prescribing and acquired knowledge and comfort with prescribing PrEP for at-risk populations.

Keywords
LGBTQ+, HIV, Pre-Exposure Prophylaxis, PrEP, Men Who Have Sex With Men, MSM, Sexual and Gender Minorities, Communication Skills, Cultural Competence, Diversity, Inclusion, Health Equity

Educational Objectives

By the end of this activity, learners will be able to:

1. Describe the indications for pre-exposure prophylaxis (PrEP) prescription.
2. Describe appropriate testing and medical care for PrEP initiation, maintenance, adherence, and discontinuation.
3. Demonstrate culturally competent communication skills needed for PrEP screening and patient education.

Introduction

Up to two million new HIV infections occur each year worldwide.[1] In 2017, there were 38,739 new HIV infections in the U.S., and of those, 66.4% were among men who have sex with men (MSM).[2] While there are still no vaccines to prevent HIV infections, behavioral and biomedical prevention strategies can be used to prevent or to reduce new HIV infection. Pre-exposure prophylaxis (PrEP), which uses antiretroviral medications, is an evidence-based preventative strategy shown to prevent new infection among individuals at greatest risk, such as MSM.[3-5] Studies have shown that among those who are adherent, PrEP, which uses the drugs tenofovir disoproxil fumarate and emtricitabine, can reduce the risk of HIV transmission by more than 90%.[6-8] Yet even with such a dramatic reduction in risk, PrEP is not widely prescribed.[9] One of the many reasons for this is physicians' lack of awareness recognizing at-risk populations, as well as their lack of comfort and experience prescribing PrEP.[10] Physicians need to build awareness of at-risk populations who may benefit from PrEP and to feel comfortable prescribing PrEP.

PrEP is appropriate for many populations, including people who inject drugs and high-risk cisgender/heterosexual people.[4]

Citation:
Perucho J, Alzate-Duque L, Bhuiyan A, Sánchez JP, Sánchez NF. Prep (pre-exposure prophylaxis) education for clinicians: caring for an msm patient. *MedEdPORTAL*. 2020;16:10908.
https://doi.org/10.15766/mep_2374-8265.10908

Copyright © 2020 Perucho et al. This is an open-access publication distributed under the terms of the Creative Commons Attribution license.

Additionally, not all MSM engage in high-risk sexual behaviors. Currently, the Centers for Disease Control and Prevention (CDC) has a very detailed 77-page reference document with clinical guidelines for prescribing PrEP for at-risk populations.[11] Although a plethora of clinical practice guidelines for prescribing PrEP exist, there is limited information on how to discuss sexual behaviors and PrEP with MSM patients. Furthermore, there is a paucity of educational materials modeling real-time clinical encounters to help clinicians gain comfort having these difficult conversations with their patients. Given that physicians' familiarity, competence in culturally appropriate open communication skills, and awareness of at-risk populations are hugely important in the success of PrEP as a preventative measure in MSM populations, an educational practice guideline designed for MSM populations that is succinct and easy to follow is needed.

MedEdPORTAL has one publication specific to PrEP education.[12] Its curriculum includes a PowerPoint (PPT) presentation and case scenarios. Our innovation is different in that it includes an audio-recorded PPT presentation teaching best practices in prescribing PrEP. The audio-recorded PPT provides presenters who have limited or no experience prescribing PrEP with a resource for didactic instruction. It also offers learners a way to review the material on their own time, which allows them to engage with the material at their own pace. We also added a videotaped scripted clinical encounter to model some clinical communication skills that can be used for PrEP counseling. This resource is particularly useful for participants who lack opportunities for observed scripted clinical encounters. Individuals can use the material presented in the video to inform the language they use with their unique patient populations when discussing PrEP. In a small-group discussion, participants can also review the communication skills demonstrated in the video and discuss communication modifications that will improve their PrEP counseling.

This publication focuses on describing the transformation in knowledge and comfort among attendees of the education presentation. The six-step Kern model[13] was applied as a framework for the design, implementation, and evaluation of this education innovation as indicated below:

1. *Problem identification and general needs assessment:* These were performed via literature review and input from medical students, residents, fellows, and medical school faculty. The lack of awareness, comfort, and familiarity prescribing PrEP due to inadequate educational instructional material was identified as an issue.
2. *Targeted needs assessment:* To better assess PrEP education needs across a subset of medical schools, we conducted a needs assessment to evaluate PrEP-related education. The 12-item needs assessment evaluated 167 participants' knowledge and comfort with PrEP and their recommendations for topic inclusions. The assessment was disseminated by email to students at Weill Cornell Medicine and Rutgers New Jersey Medical School. Almost 81% of the responders to our needs assessment self-reported knowledge about the risk factors for HIV transmission in sexually active individuals; however, only 25% self-reported knowledge about prescribing PrEP, and just 46% reported comfort assessing HIV risk behaviors. Additionally, almost half the respondents were not knowledgeable about tests needed prior to prescribing PrEP, and more than half were not knowledgeable about PrEP follow-up testing or PrEP side effects. Given these results, there is a clear need for PrEP education for clinicians.
3. *Goals and objectives:* Based on the literature review and results of our needs assessment, the goal of the educational workshop was to raise awareness and knowledge of PrEP and improve competence in caring for an MSM patient. Objectives included understanding the indications for PrEP prescription; describing appropriate testing and medical care for PrEP initiation, maintenance, adherence, and discontinuation; and learning culturally competent communication skills needed for PrEP screening and initiation.
4. *Educational strategies:* The material was presented via an in-person presentation. Attendees were asked to fill out a preworkshop survey. The presentation was then conducted. After the presentation, attendees were asked to complete a postworkshop survey.
5. *Implementation:* The 1-hour presentation was administered in the winter of 2019 and was offered to medical students, residents, fellows, and faculty.
6. *Evaluation and feedback:* Each participant was given the opportunity to complete a pre- and postworkshop survey to evaluate the workshop's design and content.

Methods

A team of faculty, fellows, and medical students with knowledge of PrEP and caring for members of the LGBTQ community developed, implemented, and/or evaluated the workshop. An associate professor of internal medicine with clinical PrEP-prescribing experience and research expertise, a fellow in academic medicine with teaching and research experience, and two medical students with an interest in LGBT health served as workshop facilitators.

Copyright © 2020 Perucho et al. This is an open-access publication distributed under the terms of the Creative Commons Attribution license.

The workshop featured two primary educational strategies: (1) a didactic component, offered as a PPT slide deck and an audio-recorded PPT slide presentation, instructing participants about HIV prevention methods and best practices in PrEP prescribing, and (2) a video-based scripted MSM patient encounter demonstrating appropriate communication skills for PrEP counseling. Workshops were not limited to a specific number of participants.

This workshop can serve as a stand-alone resource or be implemented in the context of a larger curriculum on LGBTQ health or HIV prevention. Participants experience transformative learning by acquiring knowledge about PrEP and appropriate clinical communication skills for its prescription among at-risk MSM.

Workshop Content by Appendix

Appendix A—PPT slide deck presentation: The content of the workshop was provided in the 38-slide PPT presentation, which outlined the core content for learners, including key terms, a brief history of HIV prevention, and best-practice guidelines for PrEP prescribing.

Appendix B—audio-guided PPT video presentation: The flow and content of the workshop were featured in this 38-minute video presentation. The presentation outlined the core content for learners, including key terms, a brief history of HIV prevention, and best-practice guidelines for PrEP prescribing. The presentation also included a scripted patient case scenario where learners could appreciate concepts learned from the lecture portion being applied in a clinical encounter. The scripted clinical encounter started at the 21:48 time mark.

Appendix C—discussion guide: The discussion guide was used to facilitate an in-person presentation of the provided PPT slides.

Appendix D—patient-physician video: This videotaped scripted clinical encounter allowd learners to view communication skills used in PrEP counseling.

Appendix E—preworkshop evaluation form: The preworkshop survey assessed participant PrEP attitudes and knowledge. Specifically, participants were asked to scale (1 = *strongly disagree,* 5 = *strongly agree*) their comfort discussing sexual behaviors, identifying at-risk MSM, and prescribing PrEP, as well as their knowledge of indications for initiating for PrEP, side effects, screening tests, and indications for discontinuation of PrEP. In addition, participants were asked specific knowledge-based questions relating to PrEP.

Appendix F—postworkshop evaluation form: After the presentation, participants were asked to complete the postworkshop evaluation, which contained the same set of questions as the pretest, plus additional questions asking participants to offer their feedback on the presentation.

In preparation for this workshop, facilitators should review the video presentation, and the pre- and postpresentation surveys. Reviewing the material should take approximately 2-3 hours. Should the facilitators choose, they can give an oral presentation using the PPT slides provided. The content in the slides is an exact replica of the audio-recorded PPT presentation.

This workshop was designed for clinicians and physicians-in-training and therefore can be presented to medical students, residents, and/or faculty. The ideal facilitators are clinicians with an MD, DO, PA, or NP degree and with experience in HIV prevention and LGBTQ health care. The entire presentation can be completed within a 1-hour block.

Materials

Additional materials needed to administer this workshop included pens, audiovisual equipment to show the PPT or video presentation, and printed copies of the pre- and postworkshop surveys.

Time Line

The suggested length of the workshop is approximately 60 minutes, with a time line suggested below:

- Preworkshop evaluation (5 minutes).
- Slides 1-38: in-person PPT presentation (25 minutes) or audio-guided PPT presentation (17 minutes).
- Videotaped scripted clinical encounter (15 minutes).
- Postworkshop evaluation (5 minutes).
- Question-and-answer period (10 minutes).

Results

This workshop was implemented at two sites: Rutgers New Jersey School of Medicine and Memorial Sloan Kettering Cancer Center (MSKCC). A total of 43 trainees completed the workshop evaluations: 12 from Rutgers and 31 from MSKCC. All 43 respondents were second- through fourth-year medical students. The attendees were from academic health centers in two different states. The workshop was facilitated by two presenters, an associate professor of internal medicine and an internal medicine resident.

Copyright © 2020 Perucho et al. This is an open-access publication distributed under the terms of the Creative Commons Attribution license.

A paired-sample *t* test ($p < .05$) and McNemar's test ($p < .05$) were used to compare pre- and postpresentation survey responses.

Changes in Comfort With Understanding When to Prescribe PrEP (Items 1-7)

Comfort with understanding when to prescribe PrEP in at-risk MSM was assessed via a 5-point Likert scale (1 = *strongly disagree,* 5 = *strongly agree*; Table 1). A paired-sample *t* test was used to analyze questions assessing level of comfort. Pre- and postworkshop survey analysis demonstrated statistically significant increases in comfort discussing sexual behaviors with MSM ($p = .001$), in comfort identifying at-risk MSM who may benefit from PrEP ($p = .023$), and in comfort prescribing PrEP to MSM ($p < .001$), as well as improved knowledge of the indications for prescribing PrEP to MSM ($p < .001$), of the side effects of PrEP ($p < .001$), and of recommended screening tests for PrEP ($p < .001$), and increased comfort in knowledge of indications for PrEP discontinuation for MSM ($p < .001$).

Changes in Knowledge About PrEP (Items 8-14)

Knowledge about PrEP for at-risk MSM was assessed via multiple-choice or true-or-false questions using pre- and postworkshop evaluations. McNemar's test was used to analyze questions assessing knowledge (Table 2). This specific test was chosen over chi-square analysis because it allowed for further analysis of the distribution of matched paired data (pre/post and correct/incorrect) for marginal homogeneity. Pre- and postworkshop survey analysis demonstrated a statistically significant increase in the proportion of correct responses for the following knowledge domains: lifetime risk of HIV diagnosis among black MSM ($p < .001$), eligibility for PrEP ($p < .001$), recommended PrEP prescription ($p = .037$), recommended PrEP follow-up ($p < .001$), and common PrEP side effects ($p < .001$). We did identify a significant change in knowledge for the following knowledge domains: recommended testing prior to prescribing PrEP and recommended clinical documentation when discontinuing PrEP.

We collected 32 narrative comments from our workshop participants. We organized the comments based on our module's learning objectives and quality of the presentation materials.

- Theme 1: curriculum describes the indications for PrEP prescription:
  - "This information was very clear. I had heard a bit about PrEP in a medical school lecture about HIV, but it was more clearly defined here who would benefit from it."
  - "It's helpful to have it spelled out who is a candidate for PrEP."
  - "This session has helped me learn more about at high risk populations for HIV infection."
- Theme 2: curriculum describes appropriate testing and medical care for PrEP candidates:
  - "Very informative. Covered all points necessary for me as a clinician so I know what tests to order when prescribing PrEP."
  - "I heard about this medication but didn't know that certain patients couldn't use it—like patients with sick kidneys."
  - "I didn't know what PrEP follow-up would look like and this was helpful to learn about. Also didn't know if and when someone could come off PrEP."
- Theme 3: curriculum describes culturally competent communication skills needed for PrEP counseling:
  - "The clinical scenario video was useful for reviewing the information presented in the PowerPoint presentation and then using it in practice."
  - "Hearing a run though of what the counseling would sound like helps me think about what I would say."
  - "I've been too nervous about discussing sex with patients. I think this video will motivate me to give it a try."

Table 1. Perceived Comfort and Knowledge Prescribing PrEP for MSM ($N = 43$)

Item	Mean Score[a]		*p*
	Preworkshop	Postworkshop	
I feel comfortable discussing sexual behaviors with MSM.	3.9	4.4	.001
I feel comfortable identifying at risk MSM who may benefit from PrEP.	3.7	4.2	.023
I feel comfortable prescribing PrEP to MSM.	3.1	4.2	<.001
I am knowledgeable about the indications for prescribing PrEP to MSM.	2.5	4.2	<.001
I am knowledgeable about the side effects of PrEP.	1.5	4.5	<.001
I am knowledgeable about the recommended screening tests for MSM interested in starting PrEP.	1.8	3.9	<.001
I am knowledgeable about indications for PrEP discontinuation for MSM.	1.7	4.3	<.001

Abbreviations: MSM, men who have sex with men; PrEP, pre-exposure prophylaxis.
[a]Rated on a 5-point Likert scale (1 = *strongly disagree,* 5 = *strongly agree*).

Copyright © 2020 Perucho et al. This is an open-access publication distributed under the terms of the Creative Commons Attribution license.

Table 2. Pre- and Postworkshop Knowledge Assessment ($N = 43$)

Question and Correct Answer	Mean Correct Responses: Preworkshop	Mean Correct Responses: Postworkshop	*p*
Which of the following tests is unnecessary prior to prescribing PrEP?... CBC.	30%	30%	NS
The lifetime risk of HIV diagnosis among black MSM is?... 1 in 2.	30%	100%	<.001
Who is NOT eligible for PrEP?... Patient with a history of pathologic fractures.	0%	53%	<.001
How is PrEP prescribed?... One tablet daily.	77%	100%	.037
After initiating PrEP, when should a patient see their doctor for repeat blood work?... 3 months.	46%	92%	<.001
What is the most common side effect of PrEP?... Headaches.	38%	84%	<.001
When discontinuing PrEP, a clinician should document all the following EXCEPT?... Recent travel outside of the United States.	84%	100%	NS

Abbreviations: CBC, complete blood count; MSM, men who have sex with men; NS, not significant; PrEP, pre-exposure prophylaxis.

- "The video was good. It would be great to see future videos with a transgender patient and see how that would play out differently. How are the questions different?"
- Theme 4: quality of presentation materials:
  - "Very clear learning objectives/presentation. Relevant facts and studies for analysis."
  - "The slides were broken down in a comprehensive and informational way that was pretty easy to follow."
  - "Good length, clarity, level of information."
  - "My sexual health club should have access to these materials for more learning."

Discussion

This workshop to educate future clinicians on best practices in prescribing PrEP to high-risk populations is a successful education innovation. The 1-hour workshop positively impacted participants' comfort with understanding when to prescribe PrEP and their knowledge of PrEP indications, testing, side effects, and maintenance.

We learned many lessons in the development of this workshop. Completing a needs assessment prior to developing the workshop informed the majority of the content of our presentation. We learned that when educating clinicians about HIV prevention, they need better education about high-risk communities and the clinical information patients require to make informed decisions about their health. We also learned that in addition to teaching medical facts and terms, clinicians also need real-time demonstrations of clinical communication skills used to assess HIV risk and preventive care. In our first iteration of the PrEP presentation, we did not include a videotaped scripted clinical encounter demonstrating clinical communication skills used for the assessment of risk factors associated with HIV transmission and PrEP counseling. In qualitative feedback, participants suggested that we add a real-time demonstration or videotaped demonstration of clinical communication skills used for HIV risk assessment and PrEP counseling. Based on qualitative feedback, we also added a section describing barriers to PrEP uptake and adherence.

The data from our evaluation forms revealed significant improvement in participants' comfort and knowledge pertaining to prescribing PrEP to MSM populations. Two of our knowledge questions did not reveal a significant improvement in correct responses postpresentation. One question asked, "Which of the following tests is unnecessary prior to prescribing PrEP? [Basic metabolic panel, CBC (complete blood count), hepatitis B serologies, HIV antibody.]" Approximately one-third of learners did not know that a CBC is unnecessary prior to prescribing PrEP. We hypothesize that clinicians may routinely order a CBC test when ordering any blood work and do not regularly question if it is an appropriate test for each clinical visit. They may possess a bias towards ordering a CBC test even if it is not clinically warranted. Additionally, in our early iterations of our presentations, we did not state on our slides that a CBC test was not recommended. In our final slide deck (slide 24), we now specifically state that a CBC test is not recommended prior to starting PrEP. The second knowledge question, "When discontinuing PrEP the clinician should document all of the following EXCEPT [HIV status at the time of discontinuation, Reason for PrEP discontinuation, Recent travel outside the United States, Reported sexual risk behavior]," did not yield a significant change in knowledge. Eighty-four percent of learners knew the correct documentation for PrEP discontinuation prior to workshop participation; thus, there was little room for significant improvement. Fortunately, by the end of the workshop, all learners knew the correct documentation for PrEP discontinuation.

In qualitative feedback, participants commented on their satisfaction with the content presented and the utility of the

Copyright © 2020 Perucho et al. This is an open-access publication distributed under the terms of the Creative Commons Attribution license.

videotaped scripted clinical encounter reviewing clinical communication skills used when prescribing PrEP to MSM populations. The videotaped encounter is unique in that it provides trainees with an opportunity to learn clinical language that is respectful and inclusive of sexual minorities.

While this presentation focuses on the MSM population, it is important to remember that people of all sexual and gender identities can benefit from PrEP. High-risk populations for HIV transmission include individuals with multiple sexual partners and/or intravenous drug users. Recommendations for testing before prescribing PrEP and testing during PrEP maintenance are the same for all populations, with the addition of hepatitis C screening for intravenous drug users. The CDC website maintains up-to-date information on best practices in prescribing PrEP for all at-risk populations.[14] Specific resources for other at-risk populations include websites catering to cisgender women,[15] intravenous drug users,[16] and transwomen.[17]

This module can be altered for different presenters and learners:

- Option 1: Presenter plays audio-guided PPT presentation and scripted clinical encounter. This option is recommended for presenters with no or limited experience prescribing PrEP to MSM populations.
- Option 2: Presenter performs in-person presentation using supplied discussion guide and PPT slides. The session can then conclude by viewing the videotaped scripted clinical encounter. This option is recommended for presenters with experience prescribing PrEP to MSM populations.
- Option 3: Trainees are emailed the audio-guided PPT video presentation and postworkshop evaluation form. We recommend that the instructor follow up with trainees who perform poorly on the knowledge section of the postworkshop evaluation form or those who report discomfort prescribing PrEP to MSM populations. An in-person small-group discussion would be an appropriate follow-up to improve trainees' comfort and knowledge pertaining to PrEP prescribing.

Limitations

Evaluation of this project was limited to two academic medical centers, and the majority of participants were medical students. The effectiveness of this education may vary with audience. For example, senior health professionals who are uncomfortable with discussing sexual orientation or LGBTQ health may be more uncomfortable prescribing PrEP and need additional training. Data collection from a more diverse sample would also inform enhanced PrEP education. For example, in regions of the country where there are large populations of Spanish-speaking MSM and Spanish-speaking clinicians, education can be tailored to address the social determinants of health for Spanish-speaking MSM. This innovation also does not address skills acquisition. However, future iterations could potentially do so if role-plays with learner feedback were included. Additionally, specific PrEP-prescribing practices will be subject to change as new and improved medications become available. To ensure clinicians are up to date with PrEP-prescribing guidelines, the CDC's PrEP webpage[18] can be referenced for regular updates on new studies and clinical recommendations. Finally, the impact of this workshop is limited in that we assessed its immediate influence on PrEP knowledge and comfort. Sustained comfort and knowledge may require additional training and monitoring via observed clinical encounters and continuing medical education activities.

Conclusion

We developed a 1-hour workshop that educates clinicians on best practices in prescribing PrEP for MSM populations. This workshop utilizes a PPT slide deck presentation that can be offered live and in person or presented using an audio-guided video. The workshop also includes a videotaped scripted clinical encounter that demonstrates clinical communication skills used in PrEP counseling for MSM patients. This education innovation has positively impacted clinicians' comfort and knowledge with prescribing PrEP. Workshop evaluation data can be utilized to tailor PrEP education to the needs of unique subsets of MSM populations (e.g., Spanish-speaking MSM).

Appendices

A. Presentation.pptx
B. Presentation with Audio.pptx
C. Discussion Guide.docx
D. Patient-Physician Video.mp4
E. Preworkshop Evaluation.docx
F. Postworkshop Evaluation.docx

All appendices are peer reviewed as integral parts of the Original Publication.

James Perucho, MLS: Medical Student, Cooper Medical School of Rowan University

Luis Alzate-Duque, MD: Academic Medicine Fellow, Hispanic Center of Excellence, Rutgers New Jersey Medical School

Copyright © 2020 Perucho et al. This is an open-access publication distributed under the terms of the Creative Commons Attribution license.

Amir Bhuiyan: Medical Student, Kansas City University College of Osteopathic Medicine

John Paul Sánchez, MD, MPH: Associate Professor, Emergency Medicine, Rutgers New Jersey Medical School; Associate Dean, Diversity and Inclusion, Rutgers New Jersey Medical School

Nelson Felix Sánchez, MD: Associate Professor of Medicine, Weill Cornell Medicine; Associate Attending, Memorial Sloan Kettering Cancer Center; Chair, WCM LGBTQ+ Steering Committee

Acknowledgments
We would like to thank the staff at Weill Cornell Medicine's Information Technologies and Services for their assistance filming the videotaped scripted clinical encounter.

Disclosures
None to report.

Funding/Support
None to report.

Ethical Approval
The Institutional Review Boards of the Memorial Sloan Kettering Cancer Center and Rutgers New Jersey Medical School approved this study.

References

1 Global statistics. HIV.gov website. Updated July 11, 2018. https://www.hiv.gov/hiv-basics/overview/data-and-trends/global-statistics

2. U.S. statistics. HIV.gov website. Updated July 11, 2018. https://www.hiv.gov/hiv-basics/overview/data-and-trends/statistics

3. Petroll A, Bogart LM, Horvath KJ, Mcauliffe T. PrEP knowledge, attitudes, awareness, and experience among a national sample of US primary care and HIV providers. *Presented at: 10th International Conference on HIV Treatment and Prevention Adherence*; June 28-30, 2015; Miami, FL.

4. Maartens G, Celum C, Lewin SR. HIV infection: epidemiology, pathogenesis, treatment, and prevention. *Lancet*. 2014; 384(9939):258-271. https://doi.org/10.1016/S0140-6736(14)60164-1

5. Jones A, Cremin I, Abdullah F, et al. Transformation of HIV from pandemic to low-endemic levels: a public health approach to combination prevention. *Lancet*. 2014; 384(9939):272-279. https://doi.org/10.1016/S0140-6736(13)62230-8

6. Grossman H, Anderson P, Grant R, et al. Newly acquired HIV-1 infection with multi-drug resistant (MDR) HIV-1 in a patient on TDF/FTC-based PrEP. *Presented at: HIV Research for Prevention Conference*; October 17-21, 2016; Chicago, IL.

7. Hoornenborg E, de Bree GJ. Acute infection with a wild-type HIV-1 virus in a PrEP user with high TDF levels. *Presented at: Conference on Retroviruses and Opportunistic Infections*; February 13-16, 2017; Seattle, WA.

8. Knox DC, Anderson PL, Harrigan PR, Tan DHS. Multidrug-resistant HIV-1 infection despite preexposure prophylaxis. *N Engl J Med*. 2017;376(5):501-502. https://doi.org/10.1056/NEJMc1611639

9. PrEP Mapping: first ever data on PrEP users across the U.S. *AIDSVu website*. Updated March 6, 2018. https://aidsvu.org/prep/

10. Krakower DS, Mayer KH. The role of healthcare providers in the roll out of preexposure prophylaxis. *Curr Opin HIV AIDS*. 2016; 11(1):41-48. https://doi.org/10.1097/COH.0000000000000206

11. Public Health Service US. Preexposure prophylaxis for the prevention of HIV infection in the United States—2017 update: a clinical practice guideline. *Centers for Disease Control and Prevention website. Published* March 2018. https://www.cdc.gov/hiv/pdf/risk/prep/cdc-hiv-prep-guidelines-2017.pdf

12. Bunting SR, Saqueton R, Batteson TJ. A guide for designing student-led, interprofessional community education initiatives about HIV risk and pre-exposure prophylaxis. *MedEdPORTAL*. 2019;15:10818. https://doi.org/10.15766/mep_2374-8265.10818

13. Thomas PA, Kern DE, Hughes MT, Chen BY, eds. *Curriculum Development for Medical Education: A Six-Step Approach*. 3rd ed. Johns Hopkins University Press; 2016.

14. Pre-exposure prophylaxis (PrEP). Centers for Disease Control and Prevention website. *Updated December* 13, 2019. https://www.cdc.gov/hiv/risk/prep/index.html

15. PrEP for women. The Well Project website. Updated May 23, 2019. https://www.thewellproject.org/hiv-information/prep-women

16. Injection drug use and HIV risk. Centers for Disease Control and Prevention website. *Updated February* 6, 2020. https://www.cdc.gov/hiv/risk/idu.html

17. HIV prevention and care for the transgender population. Centers for Disease Control and Prevention website. *Updated April* 1, 2020. https://www.cdc.gov/hiv/clinicians/transforming-health/transgender-patients/prep-pep.html

18. PrEP. Centers for Disease Control and Prevention website. *Updated December* 3, 2019. https://www.cdc.gov/hiv/basics/prep.html

Received: June 4, 2019
Accepted: November 21, 2019
Published: May 29, 2020

Copyright © 2020 Perucho et al. This is an open-access publication distributed under the terms of the Creative Commons Attribution license.
